# Comparative Genomics of *Bacillus amyloliquefaciens* Strains Reveals a Core Genome with Traits for Habitat Adaptation and a Secondary Metabolites Rich Accessory Genome

**DOI:** 10.3389/fmicb.2017.01438

**Published:** 2017-08-03

**Authors:** Lassaad Belbahri, Ali Chenari Bouket, Imen Rekik, Faizah N. Alenezi, Armelle Vallat, Lenka Luptakova, Eva Petrovova, Tomasz Oszako, Semcheddine Cherrad, Sébastien Vacher, Mostafa E. Rateb

**Affiliations:** ^1^Laboratory of Soil Biology, University of Neuchatel Neuchatel, Switzerland; ^2^NextBiotech Agareb, Tunisia; ^3^Graduate School of Life and Environmental Sciences, Osaka Prefecture University Sakai, Japan; ^4^Young Researchers and Elite Club, Tabriz Branch, Islamic Azad University Tabriz, Iran; ^5^Neuchâtel Platform of Analytical Chemistry, Institute of Chemistry, University of Neuchâtel Neuchâtel, Switzerland; ^6^Department of Biology and Genetics, Institute of Biology, Zoology and Radiobiology, University of Veterinary Medicine and Pharmacy Kosice, Slovakia; ^7^Institute of Anatomy, University of Veterinary Medicine and Pharmacy Kosice, Slovakia; ^8^Forest Research Institute Raszyn, Poland; ^9^CONIPHY, Parc d'activités en Chuel Quincieux, France; ^10^CONIDIA, Parc d'activités en Chuel Quincieux, France; ^11^School of Science and Sport, University of the West of Scotland Paisley, United Kingdom

**Keywords:** bioinformatics, genome mining, *Bacillus amyloliquefaciens*, biocontrol bacteria, secondary metabolism

## Abstract

The Gram positive, non-pathogenic endospore-forming soil inhabiting prokaryote *Bacillus amyloliquefaciens* is a plant growth-promoting rhizobacterium. *Bacillus amyloliquefaciens* processes wide biocontrol abilities and numerous strains have been reported to suppress diverse bacterial, fungal and fungal-like pathogens. Knowledge about strain level biocontrol abilities is warranted to translate this knowledge into developing more efficient biocontrol agents and bio-fertilizers. Ever-expanding genome studies of *B. amyloliquefaciens* are showing tremendous increase in strain-specific new secondary metabolite clusters which play key roles in the suppression of pathogens and plant growth promotion. In this report, we have used genome mining of all sequenced *B. amyloliquefaciens* genomes to highlight species boundaries, the diverse strategies used by different strains to promote plant growth and the diversity of their secondary metabolites. Genome composition of the targeted strains suggest regions of genomic plasticity that shape the structure and function of these genomes and govern strain adaptation to different niches. Our results indicated that *B. amyloliquefaciens*: (i) suffer taxonomic imprecision that blurs the debate over inter-strain genome diversity and dynamics, (ii) have diverse strategies to promote plant growth and development, (iii) have an unlocked, yet to be delimited impressive arsenal of secondary metabolites and products, (iv) have large number of so-called orphan gene clusters, i.e., biosynthetic clusters for which the corresponding metabolites are yet unknown, and (v) have a dynamic pan genome with a secondary metabolite rich accessory genome.

## Introduction

As public pressure mounts to protect the environment, biological control strategies of phytopathogens including viruses, bacteria, fungi, and oomycetes (Lara and Belbahri, 2011; Olson et al., 2012; Luchi et al., 2013; Prospero et al., 2013; Abad et al., 2014) are more considered as ecologically sound and economically viable alternatives to pesticide usage strategies (Gurr and You, 2016; Mefteh et al., 2017). Plant-associated *B. amyloliquefaciens* strains colonize plant rhizosphere, promote plant growth and suppress competing phytopathogens. Therefore, they have been widely used as biofertilizers and biopesticides (Wu et al., 2015). Abilities to compete with pathogens are linked to the production of secondary metabolites (Chen et al., 2007; Boottanun et al., 2017) that possess antimicrobial activity (Alenezi et al., 2015a,b; Belbahri et al., 2015; Alenezi et al., 2016a,b, 2017; Mefteh et al., 2017) or host plant immune system stimulation (Chowdhury et al., 2015). Secondary metabolites have been widely documented in the fields of food processing (Chang et al., 2015; Chaves-Lopez et al., 2015), pharmaceuticals (Prazdnova et al., 2015) and environmental engineering (Alvarez et al., 2015; Mlaik et al., 2015; Sellami et al., 2016).

*Bacillus amyloliquefaciens* promotes plant growth using diverse mechanisms including indole-3-acetic acid (IAA) synthesis (Shao et al., 2015; Liu et al., 2016), phosphorus solubilisation (Ravari and Heidarzadeh, 2014) and potassium solubilisation (Shakeel et al., 2015). Extracellular phytase, for instance, is considered as a plant growth promoting factor for improvement of phosphorus-use efficiency by plants (Shao et al., 2015). *Bacillus amyloliquefaciens* has also been used as biocontrol of numerous plant diseases caused by soil-borne microorganisms (Islam et al., 2016; Tan et al., 2016), post-harvest pathogens (Chen et al., 2016), insects (Aziz et al., 2016), nematodes (Castaneda-Alvarez et al., 2016), and aphids (Gadhave and Gange, 2016). Moreover, *B. amyloliquefaciens* has been reported to directly antagonize plant pathogens by competing for essential nutrients (Wu et al., 2016), producing antibiotic compounds (Srivastava et al., 2016) and inducing systemic acquired resistance (Ng et al., 2016). Volatile components such as acetoin have shown to be a potent inducer of systemic acquired resistance in plants (Magno-Perez-Bryan et al., 2015). Cyclic dipeptide such as cyclo(L-leucyl-L-prolyl) mitigates virulence in pathogenic bacteria (Gowrishankar et al., 2016). Additionally, biofilm-producing bacteria on the plant-root surfaces show promise for the use in the control of soil-borne pathogens (Tan et al., 2016). Therefore, it is currently regarded as promising environmental friendly means for crop protection (Wei et al., 2015). Recently, using comparable concentrations of *B. amyloliquefaciens* to those expected when the bacteria are used as Plant growth-promoting rhizobacteria (PGPR) and biocontrol agent (10^7^ cells ml^−^^1^) proved non harmful to the non-target soil dwelling earthworms (Lagerlof et al., 2015). Therefore, *B. amyloliquefaciens* could be safely used to optimize ecosystem services and resilience toward the development of sustainable agricultural systems.

Besides, being used as PGPR bacteria with wide metabolic capabilities, *B. amyloliquefaciens* is used for new applications such as degradation of crude oil from oil-contaminated soils (Zhang, J. H. et al., 2016), feather degradation (Yang et al., 2016), production of proteases (Wang et al., 2016), feruloyl esterases (Wang et al., 2017), and phytases (Verma et al., 2016) for industrial and food applications. Moreover, it is widely used for extraction of lipases for biodiesel production (Saengsanga et al., 2016), biosorbent for the removal of pollutants (Sun et al., 2016) and their degradation (Zuhlke et al., 2016), production of biosurfactants and antimicrobial lipopeptides (Perez et al., 2017; Zhi et al., 2017), probiotics (Gowrishankar et al., 2016), and food preservation (Eom and Choi, 2016; Calvo et al., 2017).

Comparative genomic analysis in *B. amylioliquefaciens* is made possible by the recent sequencing of multiple strains of the species. Similar to other bacterial groups the conserved “core” genome is defined as the shared genetic material among nearly all the strains of the species. The core genome contains majority of housekeeping genes and is interspersed with “accessory” genomic parts. It is believed that accessory genome is present in some strains while being absent in the rest of the species strains (Ozer et al., 2014).

In the current study, genomes of 48 strains of *B. amylioliquefaciens* available in GenBank (genomes submitted until December, 2016) have been mined for genes contributing to plant-beneficial functions and therefore, plant growth promotion potential and secondary metabolite arsenal. The contribution of core and accessory genome to plant growth promotion and secondary metabolite biosynthesis are also discussed.

## Materials and methods

### Selection of genomes and genome phylogeny

Genomes of *B. amyloliquefaciens* used in the study were selected among those submitted until December, 2016 in GenBank DNA database. They all have been deposited under the nomination *B. amyloliquefaciens*. The genomes and their corresponding strains have been described in Table 1. Nucleotide as well as the amino acid sequences of the whole genomes and the deduced coding sequences were retrieved from the GenBank DNA database for all strains (Table 1). Whole genome alignments have been conducted using REALPHY (The Reference sequence alignment based phylogeny builder, available at http://realphy.unibas.ch; Bertels et al., 2014). A Maximum Likelihood (ML) algorithm (Felsenstein, 1981) as implemented in MEGA v. 6 (Tamura et al., 2013) with evolutionary distances computed using the Kimura 2-parameter model (Kimura, 1980) was used to build the phylogenetic tree. Validity of branches in the resulting tree was evaluated by bootstrap re-sampling support of the data sets with 1,000 replications. Average nucleotide identity (ANI) values of *B. amyloliquefaciens* strains were estimated using the algorithm developed by Goris et al. (2007) combined with the 95~96% cut-off for species boundary proposed by Richter and Rosselló-Móra (2009), as implemented in the server EzBioCloud available at http://www.ezbiocloud.net/tools/ani (Yoon et al., 2017). *In silico* genome-to-genome distance values were calculated using the web-based DSMZ service available at http://ggdc.dsmz.de (Meier-Kolthoff et al., 2013). Species and sub-species cut-off were those suggested by default analysis (70%).

**Table 1:** List and description of the strains used in the study.

**Species**	**Strain**	**Genome size (Mb)**	**Plasmid**	**Description**	**References**
*Bacillus amyloliquefaciens*	DSM 7	3.9802	N	Originally described as a potent producer of liquefying amylase and other extracellular enzymes of industrial importance and isolated from infested soil in Germany; Unable to colonize *Arabidopsis* roots	FN597644.1
	TA208	3.93751	N	A strain for industrial production of guanosine and synthesis of ribavirin by assimilation of formamide	CP002627.1
	LL3	4.00199	Y	Isolated from fermented food and presents the glutamic acid-independent production of poly-γ-glutamic acid	CP002634.1
	XH7	3.9392	N	Is used to produce purine nucleosides in industry	CP002927.1
	IT-45	3.93687	Y	A commercial strain used in horticulture as plant growth promoting rhizobacteria	CP004065.1
	Y2	4.23862	N	Plant growth promoting strain	CP003332.1
				Isolated from wheat rhizosphere	
				Suppresses a broad spectrum of pathogenic fungi, such as *Ophthora capsici, Colletotrichum orbiculare, Fusarium moniliform*, and *Magnaporthe griseus*	
	CC178	3.91683	N	Isolated from the phyllosphere of cucumber; suppresses a broad spectrum of pathogenic fungi, including *Fusarium oxysporum, Phytophthora capsici, Rhizoctonia solani*, and *Sclerotinia sclerotiorum*	CP006845.1
	LFB112	3.94275	N	Formerly labeled as *Bacillus subtilis* LFB112	CP006952.1
				Isolated from Chinese herbs	
				Displayed a broad inhibitory activity against an array of pathogens involved in domestic animal diseases.	
	L-H15	3.90597	N	A plant growth promoting rhizobacteria (PGPR)	CP010556.1
				Isolated from the cucumber seedling substrate collected in Beijing, China	
				An important producer of a new bioactive lipopeptide iturin A via non-ribosomal peptide synthetases (NRPSs) with the structure of a cyclic heptapeptide linked to a 15 carbons b-amino fatty acid chain Strong inhibition ability against *Fusarium oxysporum*, a broad-host pathogen causing wilt disease in plants and other plant pathogens like *Rhizoctonia solani* and *Phytophthora capsici*	
				Containing genes related to the plant growth promotion hormone such as indole-3-acetic acid (IAA) and acetoin secretion.	
	KHG19	3.95336	N	Isolated from Korean traditional doenjang as a starter in the production of functional soya bean paste	CP007242.1
	L-S60	3.90302	N	A Gram-positive plant-associated bacterium, stimulated plant growth and showed strong antifungal function,	CP011278.1
				Isolated from the turfy soil in Beijing, China	CP011278.1
	MBE1283	3.97993	Y	Isolated from Korean traditional alcoholic beverage	CP013727.1
	CECT 8237	4.03464	N	Contributed to plant health by facing microbial pathogens or inducing the plant's defense mechanisms	CP006960.1
	CECT 8238	4.00514	N	Contributed to plant health by facing microbial pathogens or inducing the plant's defense mechanisms	CP006058.1
	B15	4.00675	N	Strong antifungal activity, isolated from grape skin in Xinjiang, China	CP014783.1
	DC-12	4.01656	N	Isolated from fermented soya beans, China (Guangzhou city)	AMQI01000001.1
	CMW1	3.90857	N	An ionic liquid-tolerant bacterium	BBLH01000001.1
	CMW1	3.90857	N	Isolated from a Japanese fermented soybean paste.	
	TF28	3.98764	N	Isolated from soybean root	JUDU01000001.1
				Strong antifungal activity *in vitro*	
				Highest antifungal activity against the rice bakanae fungus *Fusarium moniliforme*	
				Extracted lipopeptides also inhibited the growth of other phytopathogens such as *Botrytis cinerea, Fusarium oxysporum*	
				The crude lipopetides were very stable to heat and insensitive to pH.	
	RHNK22	3.97818	N	Isolated from groundnut rhizosphere	LMAG01000001.1
				Direct and indirect plant growth-promoting traits Biosurfactant activity	
				Reduction in surface tension of water	
				Biosurfactants were identified as lipopeptides (surfactin, iturin, and fengycin)	
	EGD-AQ14	4.22259	N	Isolated from saline desert plant rhizosphere of Kachchh, Gujarat (India)	AVQH01000001.1
	UASWS BA1	3.94409	N	Isolated from inner wood tissues of a decaying Platanus × acerifolia tree (Geneva, Switzerland)	AWQY01000001.1
				Antagonistic to several plant pathogenic fungi and oomycetes	
	EBL11	3.92932	N	Promoted plant growth by inhibiting the growth of fungi on plant surfaces	JCOC01000001.1
				Providing nutrients as a non-chemical biofertilizer	
	X1	3.9211	N	Isolated from Wuhan, Hubei (China)	JQNZ01000001.1
	HB-26	3.98936	N	Isolated from soil in China	AUWK01000001.1
				Secreted bioactive metabolites	
				Specific activity against *Plasmodiophora brassicae* and nematode	
	JJC33M	3.96166	N	Produces α-amylase (EC 3.2.1.1) not dependent on calcium	JTJG01000001.1
				Isolated from sugarcane soil, Papaloapan region (Mexico)	
				Capability of being stable at 40°C, indicated its possible application in the baking industry	
	LPL-K103	3.87327	N	Isolated from lemon samples (China)	JXAT01000001.1
	Lx-11	3.88689	N	Isolated from soil, Jiangsu (China)	AUNG01000001.1
				Biocontrol activity against *Xanthomonas oryzae*	
	629	3.90337	N	Colonizes different host and plant tissues under both sterile and non-sterile conditions and promotes plant growth, Isolated from healthy *Theobroma cacao* L.	LGYP01000001.1
	Bs006	4.17309	N	An important plant growth-promoting rhizobacterium (PGPR)	LJAU01000001.1
				Evaluated in Colombian banana plants	
				Genes involved in plant growth and defense, including bacteriocins, ribosomally synthesized antibacterial peptides, in addition to genes that provide resistance to toxic compounds	
	XK-4-1	3.94181	N	A bacterial plant-growth-promoting endophyte	LJDI01000001.1
	Jxnuwx-1	4.08932	N	Fibrinolytic enzyme producing *Bacillus amyloliquefaciens* JXNUWX-1 from lobster sauces	LMAT01000001.1
	H57	3.95883	N	Isolated from lucerne leaves (Australia)	LMUC01000001.1
	M49	3.88665	N	Isolated from Ulu Slim Hot Spring (Malaysia)	LQQW01000001.1
	11B91	4.02366	N	Isolated from marine environments (China)	LPUP01000001.1
	B4140	4.01425	N	Isolated from pizza	LQYO01000001.1
	B425	3.9682	N	Isolated from sterilized milk	LQYP01000001.1
	B1895	4.10728	N	Originally identified as *B. subtilis*	JMEG01000001.1
	B1895	4.10728	N	Isolated from Russia	
	12B	7.59676	N	Isolated from industrial and agricultural soil across Serbia Screened for laccase activity	JZDI01000001.1
	JRS5	4.03148	N	–	CYHL01000001.1
	JRS8	4.0909	N	–	CYHP01000001.1
	S499	3.93593	Y	Induction of systemic resistance (ISR) in tomato and bean	CP014700.1
	RD7-7	3.68821	N	Isolated from rice doenjang (Korean fermented soybean paste), a traditional Korean fermented soybean food, showed antimicrobial activity against *B. cereus* and regulated its toxin gene expression	CP016913.1
	SRCM101266	3.76536	N	Isolated from kochujang (hot red pepper paste) (South Korea)	LYUG01000001.1
	SRCM101294	3.96275	N	Isolated from kochujang (hot red pepper paste) (South Korea)	LZZO01000001.1
	K2	3.92677	N	Isolated from rhizophere soil of mangrove (Thailand)	MOEA01000001.1
	WS-8	3.92979	N	Isolated from rhizophere soil of grove (China)	CP018200.1
	Y14	3.95716	N	Isolated from rhizophere soil of peanut (China)	CP017953.1
	LM2303	3.98939	N	Isolated from alpine steppe (China)	CP018152.1

### Homology based mining of genes contributing to plant-beneficial functions

#### Nutrient acquisition

The nitrogenase-encoding genes *nifHDK, nifS*, and *nifU* responsible for nitrogen fixation in proteobacterial PGPR from *Azospirillum, Burkholderia*, and *Bacillus* were used as bait to search for similar sequences (Bruto et al., 2014). The pyrroloquinoline quinone-encoding genes *pqqBCDEFG* in the PGPR *Pseudomonas fluorescens* F113, *Erwinia herbicola*, and *Enterobacter intermedium* (Liu et al., 1992 and Kim et al., 2003; Miller et al., 2010) were used to mine the studied genomes. The gene encoding the *B. velezensis* SQR9 3-phytase was selected to mine *B. amyloliquefaciens* genomes for phytase production (Shao et al., 2015). Genes encoding *ureABC* of *Bacillus subtilis* (strain 168) was used in blast searches to recover urease genes in *B. amyloliquefaciens* studied genomes (Niazi et al., 2014). Exoenzyme genome mining was carried out using either keyword search in the different genomes followed by checking of secretion using SignalP 4.1 (Petersen et al., 2011) or by blasting exoenzyme sequences described in closely related species (Niazi et al., 2014). Enzymes targeted were proteases, lipases, cellulases, pectinases, amylases, laccases, xylanases, and lichenases. Heat-shock protein genes *dnaJ, dnaK*, and *groE*, cold shock protein genes *cspA, cspC, cspD*, and *cspE* (Gupta et al., 2014), osmoprotectant glycine betaine synthesis genes *gbsAB* (Boch et al., 1996). Genes encoding phenazine (*phzADEFG*) were also mined since phenazine aid in long term survival and ability to compete with the resident microflora (Mazzola et al., 1992).

#### Root colonization and growth promotion factors

The presence of gene clusters (*flgBCDEGKLMN, flhABFOP*) and the *swrABC* gene cluster have been searched in the genomes of the different *B. amyloliquefaciens* targeted genomes (Ghelardi et al., 2012). *che*/*fla*/*fli/tlp/mcp* operons involved in the regulation of *B. subtilis* chemotactic response and their relatives in the genome of *Bacillus velezensis* UCMB5113, *motABPS* cluster responsible for cell-envelope and cellular processes motility and chemotaxis, have been mined in the different genomes studied (Niazi et al., 2014). The *xerCD* genes, site recombinase, are critical for the PGPRs to be effective rhizosphere colonizers (Shen et al., 2013) have been mined. Annotation and homology-based searches were conducted in the *Bacillus* genomes for genes encoding exopolysaccharide using *B. subtilis epsA-O* operon genes, *tapA, tasA, sipW, pgsB*, and *bslA* (Vlamakis et al., 2013).

#### Plant growth-promoting traits: hormones

The genes involved in the tryptophan-dependent pathways for synthesis of the auxinic phytohormone indole acetic acid (IAA) in the closely related *B. velezensis* FZB42 and *B. velezensis* SQR9 (Idris et al., 2007; Shao et al., 2015) were selected. The different pathways mined were: (i) indole-3-pyruvate (IPyA) pathway involving the tryptophan transaminase (*patB*), indole-3-pyruvate decarboxylase (*YclC* and *YclB*) and indole-3-acetaldehyde dehydrogenase (*DhaS*) genes, (ii) indole-3-acetonitrile (IAN) involving the nitrilase gene (*yhcX*), (iii) uncharacterized IAA biosynthesis pathway involving tryptophan acetyltransferase gene (ysnE) and (Zimmer et al., 1991; Idris et al., 2007; Shao et al., 2015). Additionally, the *ywkB* gene involved in the transport of auxin out of the bacterial cell, its redistribution to the plant roots, and encoding a putative auxin efflux carrier protein was also mined in the different genomes (Niazi et al., 2014).

The *Agrobacterium tumefaciens* trans-zeatin synthase, *tzs* gene and the *miaA* gene encoding tRNA dimethylallyl transferase that removes zeatin precursor from tRNA were used to query the collected genomes (Vacheron et al., 2013).

The *IpdC* gene directs the production of phenylacetic acid (PAA), having weak auxin activity and antimicrobial against both bacteria and fungi in *Azospirillum brasilense* (Somers et al., 2005). As in *Azospirillum*, the *B. simplex* genome has the *paa* operon (data not shown), which is important for the degradation of PAA.

Genes encoding ACC deaminase structural genes (*acdS*) and leucine responsive regulatory protein (LRP) gene (*acdR*) of *Pseudomonas putida* GR12-2 were selected to mine *B. amyloliquefaciens* analyzed genomes (Glick et al., 1994).

The gene of *A. brasilense* Sp245 *nirK* copper nitrite reductase and *Bacillus* nitric oxide synthase (*nos*) genes leading to formation of NO and hence root branching was used to mine the *B. amyloliquefaciens* genomes (Bruto et al., 2014).

In *B. subtilis* OKB105 polyamines such as spermine, spermidine, and putrescine have PGP properties (Xie et al., 2014). Genes involved in polyamine synthesis such as *speA* (agmatine synthesis), *speB* (putrescine synthesis); *speD* and *speE* (spermidine synthesis) and *metK*, responsible for the conversion of methionine to S-adenosyl-methionine were mined. Genes for various binding proteins, permeases, and transporters for polyamines have also been mined by keyword searches in the different genomes.

#### Plant protection from oxidative stress (antioxidant enzymes)

The battery of enzymes produced by *Bacillus* spp. in response to oxidative stress has been fetched in the different *B. amyloliquefaciens* genomes. In *B. velezensis* UCMB5113 superoxide dismutases (*SodA, SodC*, and *SodF*), three hydrogen peroxide decomposing catalases (*KatA, KatE*, and *KatX*), manganese catalase (*YdbD*), three alkyl hydroperoxide reductases (*AhpC, AhpF*, and BASU_0830), thiol peroxidase (*tpx*), glutathione peroxidase (*gpo*), bacillopeptidase F (*bpr*), gamma-glutamyl transpeptidase (*ggt*), and an operon (*ohrARB*) for resistance to organic peroxides have been described by Niazi et al. (2014) and included in our genome mining efforts. The flavohemoprotein nitric oxide dioxygenase encoded by the *B. velezensis* UCMB5113 genes *hmp* and BASU_2738, that protect the bacterium from nitrosative stress have also been included in our study. The genes *gacS, soxS, soxR*, and *oxyR* involved in plant protection against oxidative stress were also mined (Whistler et al., 1998; Ochsner et al., 2000).

#### Plant induction of disease resistance

The *P. aeruginosa* genes have been mined in the different genomes. Genes selected for homology-based searches involved the *B. velezensis* SQR9 genes encoding acetoin biosynthesis: acetolactate synthase *alsS* (E.C. 2.2.1.6), acetolactate decarboxylase *alsD* (E.C. 4.1.1.5) and the regulatory gene *alsR* as well as the gene *bdhA* encoding 2,3-butanediol dehydrogenase encoding 2,3-butandiol biosynthesis (Shao et al., 2015).

#### Antibiotics and related compounds

*hcn*ABC genes directing production of HCN in *Pseudomonas* spp. have been used to mine *B. amyloliquefaciens* genomes (Bruto et al., 2014). *phl*ACBD genes were used in blast searches to discover similar sequences in the genomes of the mined *B. amyloliquefaciens* strains (Bruto et al., 2014). *gabD* and *gabT* involved in production of pest/disease suppressing γ-aminobutyric acid (GABA) (Loper et al., 2012) have been used as baits in genome mining.

#### Resistance to drugs

Homologues of the *tetB* protein that contributes to tetracycline resistance and the tetR tetracycline operon transcriptional regulator *tetR* in *B. subtilis* have been searched in the different genomes (Sakaguchi et al., 1988). Multifunctional tetracycline-metal/H^+^ antiporter (*tetA*) have also been mined (Someya et al., 1995). The operon *yyaACDEHJKLRST* encoding a streptothricin acetyltransferase (Jacob et al., 1994) was used as a bait in the screening of homologs in the different genomes. Fosfomycin resistance gene *fosB* from *B. cereus* was used to search for homologs in the *B. amyloliquefaciens* genomes (Fu et al., 2016). *The homolog of the B. licheniformis glyoxalase/bleomycin resistance gene ykcA have been used as a bait in the blast search against mined genomes* (Rey et al., 2004)*. The homolog of the B. subtilis (strain 168)* β-lactamase *gene penP have been used as a bait in the blast search against mined genomes* (Barbe et al., 2009). Quinolone resistance *norA* homology have been searched in the different *B. amyloliquefaciens* genomes (Neyfakh et al., 1993). The *E. coli* gene floR have been mined in the *B. amyloliquefaciens* genome collection (Doublet et al., 2005). *Bacillus subtilis* 168 *aadK* gene, which encodes aminoglycoside 6-adenylyltransferase, a streptomycin-modifying enzyme, was mined in the different strains (Noguchi et al., 1993). *Bacillus subtilis ycbJ* gene encoding an aminoglycoside phosphotransferase has been used to search homologs in the genomes of the mined strains (Hosoya et al., 2002). *Bacillus subtilis* vmlR encoding antibiotic efflux ATP-binding transport protein has been used to mine the different genomes (Ohki et al., 2005). Genes encoding putative multidrug exporters have been mined from the different genomes according to Niazi et al. (2014).

#### Resistance to heavy metals

The genes *arsABC* and *ywrK* were used as a bait to detect any putative arsenic detoxification ability (Duan et al., 2013). We have mined the *copYZAB* operon formed by four genes: *copA* and *copB* that encode ATPases for influx and efflux of copper, respectively; *copZ* that encodes a copper chaperone; and *copY*, a copper responsive repressor. *CopA* encodes a major copper resistance mechanism. One-component regulators *CueR, CopY*, and *CsoR*, identified in *E. coli, E. hirae*, and *M. tuberculosis*, respectively, have also been mined (Rademacher and Masepohl, 2012). *CtpAB* and *ycnJ* genes encoding copper resistance proteins (Zhang et al., 2015) were also mined. Homologs of the *B. subtilis (strain 168) ynbB gene have been mined in the different genomes* (Barbe et al., 2009). Homology of *crcA, cspE, crcB*,*yhdV* has been mined in all the genomes (Hu et al., 1996). Homologs of the *yceGH* and *yaaN* have been searched in all genomes (Franks et al., 2014). *CzcD* encodes a cadmium, cobalt and zinc/H(+)-K(+) antiporter in *B. subtilis* and protects the cell against elevated levels of Zn(II), Cu, Co(II), and Ni(II) (Moore et al., 2005). Gene*ndoA* (*ydcE*) and antitoxin gene, *ndoAI* (*ydcD*) have been mined (Wu et al., 2011). Sensors for metals; *Fur, ArsR, MerR, NikR, DtxR, mtnR*, and *yfmP* family of metalloregulators of the *B. subtilis* genome were mined from the different *B. amyloliquefaciens* genomes (Osman and Cavet, 2010).

#### Degradation of aromatic compounds

Vanillate, 4-hydroxybenzoate, salicylic, ferulic, *p*-coumaric acids are considered as natural toxins and cause specific stress responses in microorganisms that have developed resistance against phenolic acids. Both phenolic acid decarboxylases *padC* and *bsdBCD* (*yclBCD*) of *B. subtilis* were mined. The putative LysR-type regulator encoded by *bsdA* (*yclA*) gene upstream of the *bsdBCD* operon revealed is the transcriptional activator of *bsdBCD* expression in response to phenolic acids were also mined (Graf et al., 2016). Dibenzothiophene (DBT) is the model compound for this class of molecules. The operon *dszABC* of *Rhodococcus* sp. (Piddington et al., 1995) was used to mine the genomes. Genes encoding homologs of the *B. velezenzis* FZB42 *azoR2, mhqADNOPE* have been mined in the genome of the different strains (Nguyen et al., 2007).

#### Secondary metabolite clusters identification using antismash, prism, napdos, NP.search, and bagel3

The annotated draft genome sequence files, which included information for both contigs and ORFs (Table 1) were subjected to secondary metabolite gene cluster analysis using antiSMASH 3.0 (Weber et al., 2015), prediction informatics for secondary metabolomes (PRISM) (Skinnider et al., 2015), NapDos (Ziemert et al., 2012), NP.search (Li et al., 2009), and the bacteriocin specific software BAGEL3 (Van Heel et al., 2013).

#### Identification of core genome and accessory genomes of the strain collection

Spine, used to determine the core genome, defined as those sequences present in nearly all genomes from bacteria of a given species, from the sequences of all *B. amyloliquefaciens* isolates collection (Ozer et al., 2014). Identification of accessory genomic sequences in the different *B. amyloliquefaciens* isolates genomes was performed using Agent (Ozer et al., 2014).

## Results

### Species status of *B. amyloliquefaciens*

**Figure 1:**
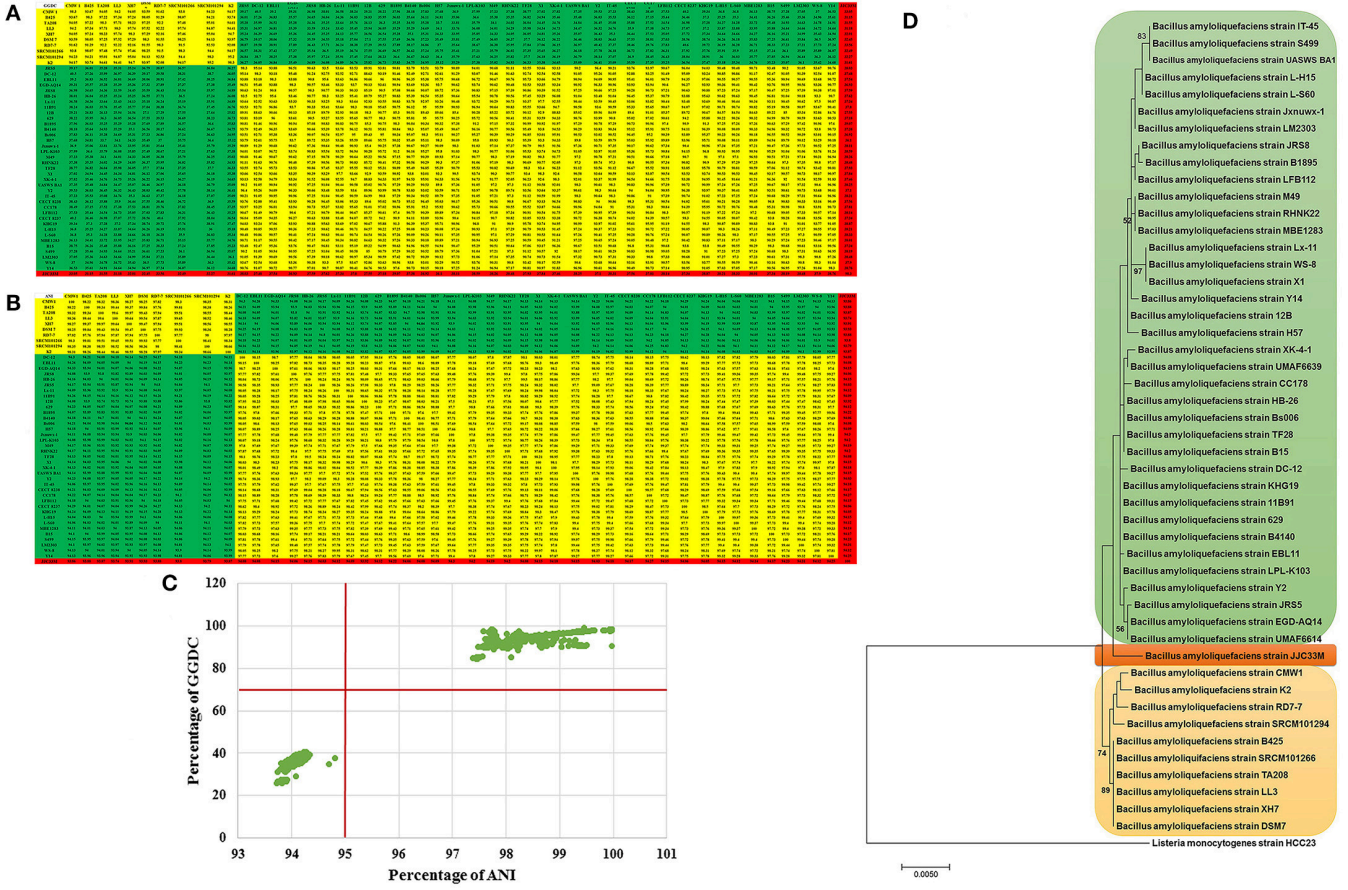
**(A,B)** Genome-to-Genome Distance Calculation (GGDC) and Average nucleotide identity (ANI) values between each indicated strains were calculated with GGDC 2 and EzBiocloud web-based programs showed 3 species candidates based on 70% and 95% similarity thresholds. **(C)** Scatter plot of ANI and GGDC values of *B. amyloliquefaciens* strains. **(D)** Maximum Likelihood phylogenomic tree of G-positive bacteria *B. amyloliquefaciens* strains. *L. monocytogenes* strain HCC23 was used as outgroup. Supports for branches were assessed by bootstrap resampling of the data set with 1,000 replications.

In total, 48 strains of the species (submitted until December 2016) have been selected for genome mining. Their genome size varied between 3.60 and 7.60 mega base pairs (MB) (Table 1). GGDC analysis revealed the presence of three species lumped together in the strains collection *sensu* Meier-Kolthoff et al. (2013), where 70 % similarity between two genomes was established as the gold standard threshold for species boundaries (Figure 1A), ANI analysis revealed also three putative species *sensu* Richter and Rosselló-Móra (2009), where 95–96% cut-off was set up to delimit species boundaries. In both analysis, a set of 10 strains represented probably the “true” *B. amyloliquefaciens* species termed “*B. amyloliquefaciens sensu stricto*” while a set of 37 strains matched *B. velezensis* and a single isolate represented new species, yet to be described (Figures 1A,B). The proposed threshold for species discrimination (70%) clearly delimit species boundaries because strain pairs were found to be between 50 and 70% GGDC distance. GGDC values plotted against ANI values (Figure 1C) showed agreement between the two technologies for species discrimination and no discontinuity in the graph could be observed. Finally, whole genome phylogeny confirmed results using GGDC and ANI values, with three sister branches representing the three species (Figure 1D).

### Bioinformatic evaluation of plant growth promotion potential of *B. amyloliquefaciens* strains

**Figure 2:**
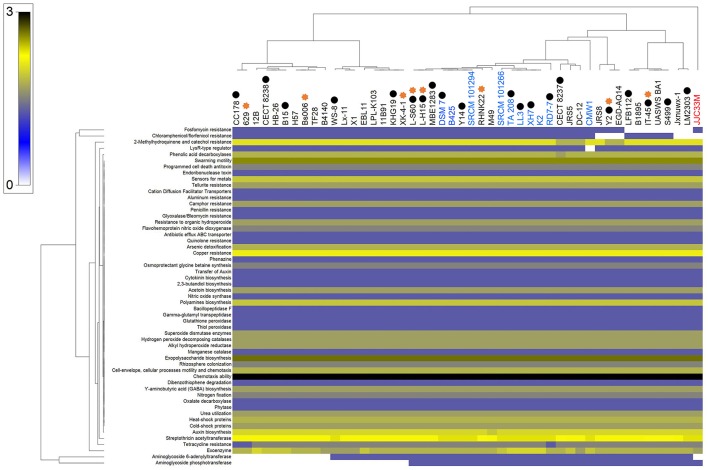
Heat map of mining of genes contributing to plant-beneficial functions in *B. amyloliquefaciens* strains. Bacterial strains belonging to the same species are highlighted with the same colors. Bacterial strains indicated with asterisk sign are related to strains that are emphasized in the literatures as plant growth promoting (PGP) bacteria. Black circles show completely sequenced strains.

Bioinformatic evaluation of plant growth promotion potential of *B. amyloliquefaciens* strains collection has been performed through homology-based mining of genes contributing to plant-beneficial functions. As unambiguously shown in Figure 2, large majority of *B. amyloliquefaciens* strains show presence of mined genes independently of whether these strains are represented by a complete coverage of the genome or their association to plant rhizosphere.

### Secondary metabolites from *B. amyloliquefaciens*

**Figure 3:**
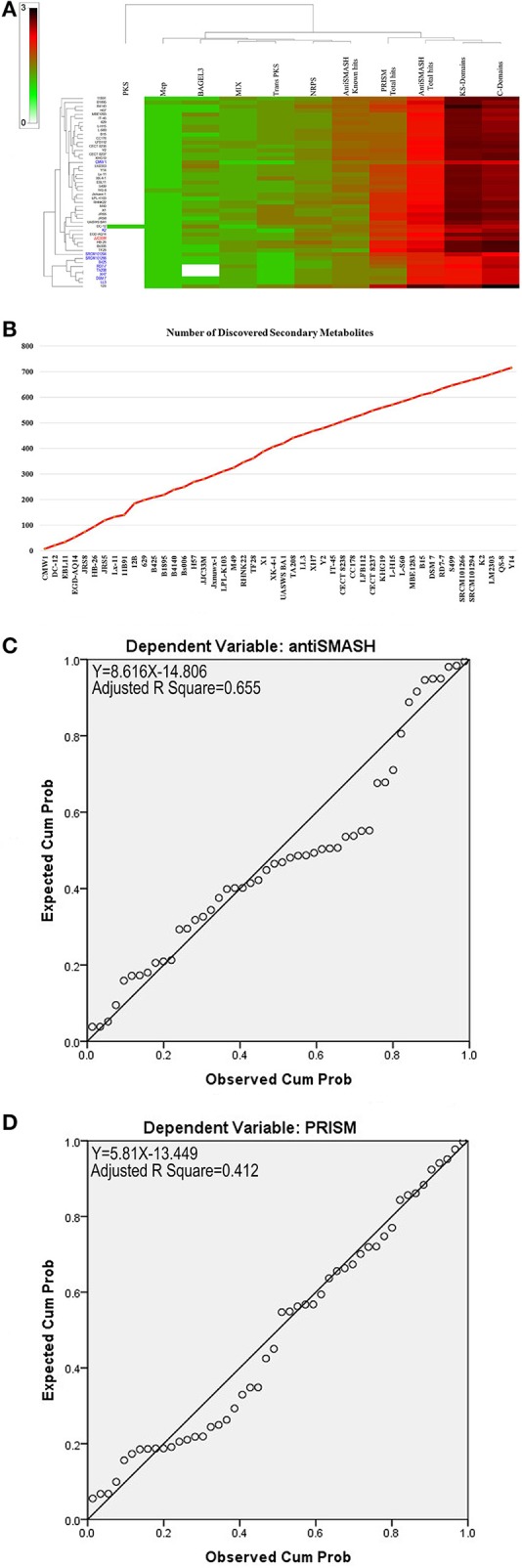
**(A)** Heat map of mining of genes contributing to secondary metabolite clusters. **(B)** Number of discovered secondary metabolites. **(C)** Statistically significant linear relationship between genome sizes and antiSmash total hits (*p* < 0.05). **(D)** Statistically significant linear relationship between genome sizes and PRISM total hits.

Secondary metabolite clusters present in the genome of the *B. amyloliquefaciens* collection have been evaluated using antiSMASH 3.0 (Weber et al., 2015), prediction informatics for secondary metabolomes (PRISM) (Skinnider et al., 2015), NapDos (Ziemert et al., 2012), NP.search (Li et al., 2009), and the bacteriocin specific software BAGEL3 (Van Heel et al., 2013). As shown in Figure 3 and Supplementary Table S1, different strains showed high levels of diverse secondary metabolite clusters using all implied programs. Rarefaction analysis of secondary metabolite clusters from the results of genome sequencing progress clearly attested that saturation could not be reached using all genome collection analyzed (Figure 3B). A very clear correlation between genome size and number of gene clusters known to be involved in secondary metabolite biosynthesis and mined by antiSMASH was found. Approximately 65% of the variance in the number of secondary metabolite clusters can be explained by genome size (Figure 3C). However, for PRISM only 41% of the variance in the number of secondary metabolite clusters can be explained by genome size (Figure 3D).

### Genomes to natural products prediction in *B. amyloliquefaciens*

**Figure 4:**
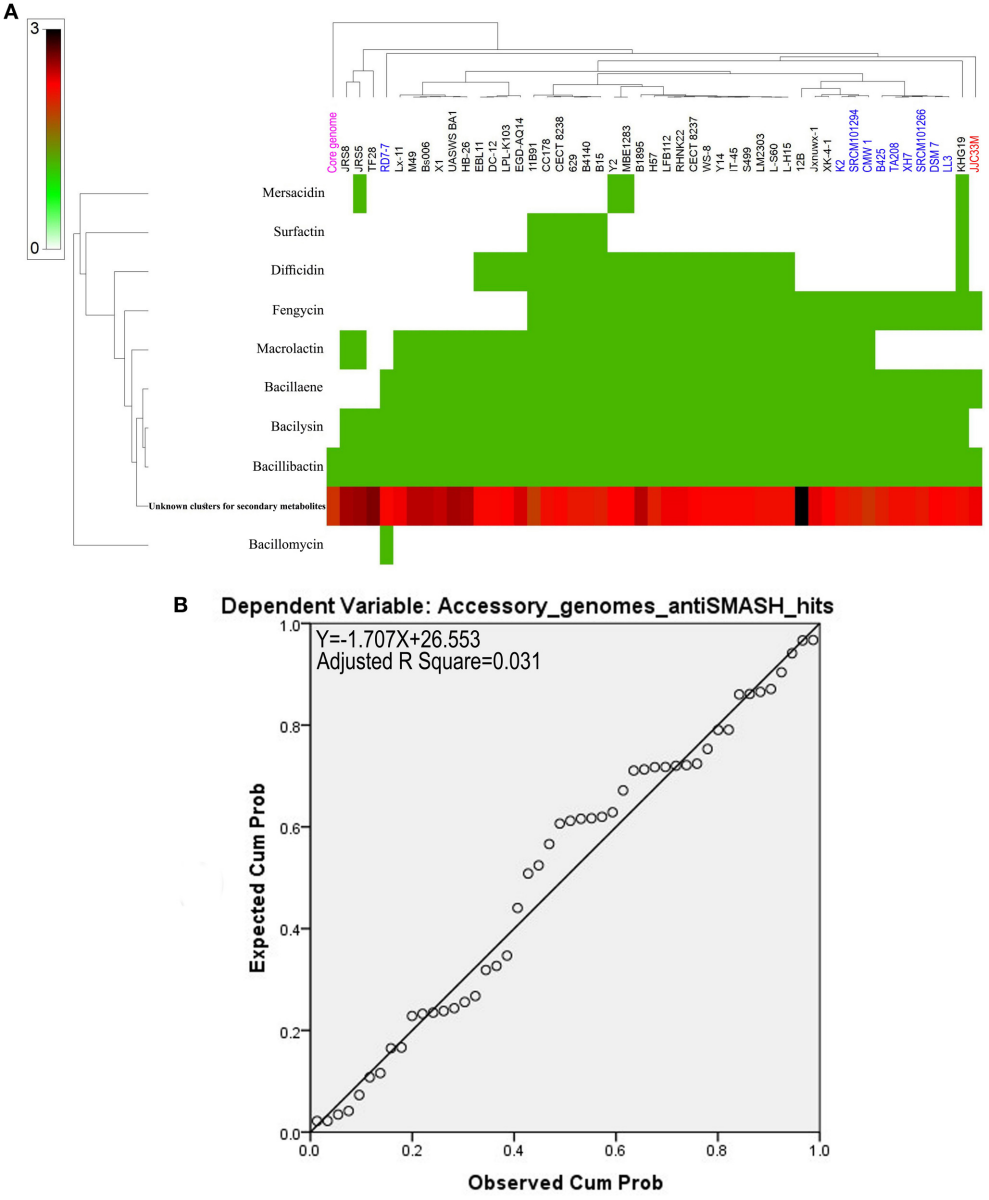
**(A)** Heat map of *B. amyloliquefaciens* accessory genome secondary metabolites. **(B)** Non-significant linear relationship between genome sizes and accessory genome antiSmash total hits (*p* > 0.1).

Natural products prediction in the core genome and the accessory genomes of the *B. amyloliquefaciens* collection revealed high numbers of unknown secondary metabolites across the strains analyzed (Figure 4A and Supplementary Table S1). Only bacillibactin could be found in all the strains and in the core genome of *B. amyloliquefaciens* (Figure 4A). All remaining known secondary metabolites such as surfactin, difficidin, fengycin, macrolactin, bacillaene, bacilysin, and mersacidin are harbored by the accessory genome of the different strains. Only 3% of the variance in the number of secondary metabolite clusters can be explained by accessory genome size (Figure 4B).

## Discussion

### Species status of *B. amyloliquefaciens*

Given the high phenotypic similarity of *B. amyloliquefaciens* to *B. subtilis* and other closely related *Bacillus* spp. such as *B. velezensis*, it is not possible to distinguish these organisms solely on the basis of conventional assays (Dunlap et al., 2015 and 2016). Sequencing of 16S rRNA gene, while has historically been used in defining bacterial taxonomy and phylogeny, proved difficult and controversial that lead to well-documented misidentifications (Hahnke et al., 2016). Therefore, considerable taxonomic confusion blurs biotechnological applications of this highly relevant group. Recently, genome based approaches such as Average Nucleotide Identity (ANI) and digital DNA-DNA hybridization (DDH) calculated using the Genome-to-Genome Distance Calculation (GGDC) complemented with genome comparisons, alignments and phylogenetic reconstructions have been suggested as alternative methods for species discrimination (Goris et al., 2007; Richter and Rosselló-Móra, 2009; Meier-Kolthoff et al., 2013). Using these accurate tools, several later heterotypic synonyms were documented in this group such as *B. methyltrophicus, B. amyloliquefaciens* subsp. *plantarum*, and *B. oryzicola* that have been shown, using phylogenomics, later heterotypic synonyms of *B. velezensis* (Dunlap et al., 2016). Therefore, phylogenomic approaches are urgently required to resolve outstanding problems in the phylogenetic systematics of the *B. subtilis* group (Dunlap et al., 2016). Phylogenomic analysis of all sequenced genomes of *B. amyloliquefaciens* strains available in GenBank, the National Centre for Biotechnology Information (NCBI) database (Table 1), allowed us to check taxonomic validity of these isolates, determine the extent of inter-species genome variability within *B. amyloliquefaciens* and reconstruct their phylogenetic relationships. Figures 1A–D clearly showed that at least three *Bacillus* spp. were lumped under the name *B. amyloliquefaciens* along with *B. amyloliquefaciens sensu stricto*. While isolates DC12, EBL11, EGD-AQ14, JRS8, HB26, JRS5, LX-11, 11B91, 12B, 629, B1895, B4140, Bs006, H57, Jxnuwx-1, LPL-K103, M49, RHNK22, TF28, UASWS BA1, Y2, IT-45, CECT 8238, CC178, LFB112, CECT 8237, KHG 19, L-H15, L-S60, MBE 1283, B15, S499, LM2303, WS-8, and Y14 matched *B. velezensis* in ANI and GGDC analysis (data not shown), JJC33M failed to match known species and should be described as a new species. *Bacillus* isolates CMW1, B425, TA208, LL3, XH7, DSM7, RD7-7, SRCM101266, SRCM101294, and K2, should therefore be regarded as *B. amyloliquefaciens sensu stricto*. Phylogenomic tree based on the core genome of all isolates of *B. amyloliquefaciens* showed consistent results with earlier observations using either ANI or GGDC values. Our findings suggest that despite the pivotal role of microbial taxonomy in industrial exploitation of microbes and their products, classification and accurate identification have often been a neglected task. We recommend inclusion of phylogenomic studies as a prerequisite gold standard to the use of the name *B. amyloliquefaciens* in new reports.

### Bioinformatic evaluation of plant growth promoting potential of *B. amyloliquefaciens* strains

Genome mining of the different strains of *B. amyloliquefaciens* allowed the discovery of numerous features documented in earlier studies as efficient factors of the interaction between host plants and the associated *B. amyloliquefaciens* strains (Niazi et al., 2014; Zhang, N. et al., 2016). These features allow nutrient acquisition, PGPR fitness, root colonization and growth promotion factors, plant growth promoting traits (hormones), plant protection from oxidative stress, plant induction of disease resistance, antibiotics and related compounds, resistance to drugs and heavy metals and degradation of aromatic compounds (Bruto et al., 2014; Niazi et al., 2014; Chen et al., 2016; Zhang, N. et al., 2016; Rekik et al., 2017). All these features were present in approximately all the genomes analyzed independently of whether these strains are represented by a complete coverage of the genome or their association to the plant rhizosphere. All these features could be also found in the core genome of the *B. amyloliquefaciens sensu-stricto* or the three-conserved species core genome. We speculate that plant growth promoting features could be considered as evolutional traits for adaptation to plant-associated habitats as suggested by Zhang, N. et al. (2016).

### Secondary metabolites from *B. amyloliquefaciens*

*Bacillus amyloliquefaciens* strains proved a prolific source of diverse secondary metabolite classes including polyketides (PKs) such as macrolactins and difficidins, peptides such as bacteriocins, lanthipeptides such as cerecidins, and lipopeptides (LPs) such as surfactins and iturins (Cimermancic et al., 2014; Wang et al., 2014; Aleti et al., 2015). PKs and LPs are the key inhibitors of plant pathogens and strains bearing these metabolites have been widely used in agriculture (Cochrane and Vederas, 2014). Despite the exponential increase of the number of *B. amyliquefaciens* genomes sequenced and the description of efficient analysis tools for secondary metabolite prediction, cursory investigation of these genome's wealth is available for describing the novelties and predicting uncharacterized metabolites (Aleti et al., 2015). In our study using recently described bioinformatic tools designed for the identification of clusters involved in secondary metabolism such as PRISM (Skinnider et al., 2015), antiSMASH 3.0 (Weber et al., 2015), NapDos (Ziemert et al., 2012), NP.searcher (Li et al., 2009), and the bacteriocin specific software BAGEL3 (Van Heel et al., 2013) and the *B. amyloliquefaciens* genomes available in databases, we documented high structural and functional diversity of secondary products in the species and their underlying gene clusters. Our data clearly showed high variety of secondary metabolites suggested by the high number of matches using five different programs for their prediction.

Rarefaction analysis of secondary metabolite clusters from the results of genome sequencing progress demonstrated clearly that saturation could not be reached using all genomes available and more sequencing effort of new strains is necessary to tackle the wide diversity of secondary metabolites potentially harbored by the species. This result confirmed the observations of Alenezi et al. (2016b) using the genus *Aneurinibacillus*. A very clear correlation between genome size and number of gene clusters known to be involved in secondary metabolite biosynthesis and mined by antiSMASH and PRISM was found. About 65% of the variance in the number of secondary metabolite clusters can be explained by genome size for antiSMASH for instance. This confirmed the results established by Jeske et al. (2013) while contrasted those conducted by Machado et al. (2015) and Alenezi et al. (2016b).

### Genomes to natural products prediction in *B. amyloliquefaciens*

Genome mining was also used to predict uncharacterized gene clusters and evaluate their potential to produce new yet to be characterized secondary metabolites. We found that while few known secondary metabolites such as surfactin, difficidin, bacilysin, fengycin, macrolactin, bacuillaene, and bacillibactin were identified, hundreds of secondary products still await for accurate molecular identification and the assignment of subsequent biological function. Similar finding has been reported by Jeske et al. (2013), Machado et al. (2015), and Alenezi et al. (2016b). Dynamics of evolution of the clusters was also investigated using comparative genomics across all known core and accessory genomes of *B. amyloliquefaciens* strains. Our findings unambiguously suggested that except bacillomycin, all remaining known or unknown secondary metabolites were harbored by the strains specific accessory genomes. This finding highlights the extraordinary potential offered by these plants associated *Bacillus* spp.

## Summary and outlook

Our findings clearly suggest plant growth promoting features as evolutional traits for adaptation of *B. amyloliquefaciens sensu lato* to plant-associated habitats. They also document large repertoire of secondary metabolites harbored by a dynamic accessory genome that warrants more genome sequencing efforts of *B. amyloliquefaciens sensu lato* in order to shed the light on the wealth of these natural products offered by these bacteria.

## Ethics statement

This research did not involve any work with human participants or animals by any of the authors.

## Author contributions

Conceived and designed the experiments: LB and AC. Performed the experiments: LB, FA, LL, IR, and AC. Analyzed the data: LB and AC. Contributed reagents/materials/analysis tools: LB. Wrote the manuscript and enriched the literature: LB. Corrected the manuscript: LB, MR, TO, LL, EP, FA, AV, SC, SV, and AC.

### Conflict of interest statement

The authors declare that the research was conducted in the absence of any commercial or financial relationships that could be construed as a potential conflict of interest.

## Supplementary material

Supplementary Table S1 Predicted secondary metabolites (antiSmash cluster hits) of Bacillus amyloliquefaciens strains.
