# Supplementary material for: Comparative Genomics of Bacillus amyloliquefaciens Strains Reveals a Core Genome with Traits for Habitat Adaptation and a Secondary Metabolites Rich Accessory Genome
Source: Front Microbiol. 2017 Aug 3;8:1438. doi: 10.3389/fmicb.2017.01438 (PMC5541019; doi:10.3389/fmicb.2017.01438)
Supplement: Supplementary Table S1 — Predicted secondary metabolites (antiSmash cluster hits) of Bacillus amyloliquefaciens strains. [file Table1.DOCX]

Table S1.

| Strain clusters | Type | Length (bp) | Most similar known clusters | Predicted core clusters |
| --- | --- | --- | --- | --- |
| **12B** | | | | |
| Cluster 1 | [Transatpks](http://antismash.secondarymetabolites.org/help#transatpks) | 85887 | Macrolactin biosynthetic gene cluster (100% of genes show similarity) | 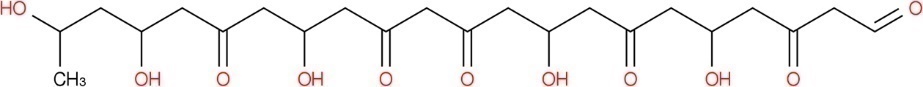 |
| Cluster 2 | Nrps-Transatpks | 102692 | Bacillaene biosynthetic gene cluster (100% of genes show similarity) | 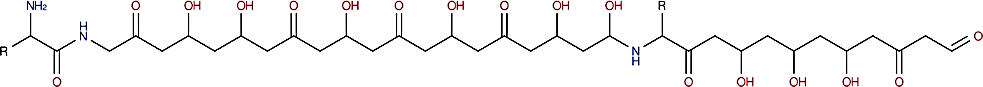 |
| Cluster 3 | Otherks | 41244 | Butirosin biosynthetic gene cluster (7% of genes show similarity) | - |
| Cluster 4 | Microcin | 20148 | - | - |
| Cluster 5 | [Nrps](http://antismash.secondarymetabolites.org/help#nrps)-[Bacteriocin](http://antismash.secondarymetabolites.org/help#bacteriocin) | 66793 | Bacillibactin biosynthetic gene cluster (100% of genes show similarity) | 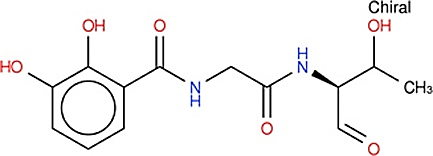 |
| Cluster 6 | Transatpks | 46683 | Difficidin biosynthetic gene cluster (46% of genes show similarity) | - |
| Cluster 7 | T3pks | 41151 | - | - |
| Cluster 8 | Terpene | 21883 | - | - |
| Cluster 9 | Nrps | 21863 | Plipastatin biosynthetic gene cluster (30% of genes show similarity) | - |
| Cluster 10 | Other | 41418 | Bacilysin biosynthetic gene cluster (85% of genes show similarity) | - |
| Cluster 11 | Transatpks | 29091 | Difficidin biosynthetic gene cluster (33% of genes show similarity) | 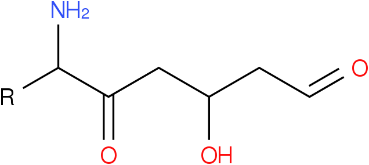 |
| Cluster 12 | Nrps | 25831 | Surfactin biosynthetic gene cluster (39% of genes show similarity) | - |
| Cluster 13 | Lantipeptide | 22609 | Locillomycin biosynthetic gene cluster (35% of genes show similarity) | - |

Table S1. Continued.

| Strain clusters | Type | Length (bp) | Most similar known clusters | Predicted core clusters |
| --- | --- | --- | --- | --- |
| **12B** | | | | |
| Cluster 14 | Nrps | 28824 | Surfactin biosynthetic gene cluster (52% of genes show similarity) | 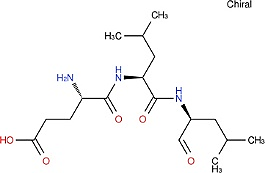 |
| Cluster 15 | Terpene | 20740 | - | - |
| Cluster 16 | Nrps-Transatpks | 77182 | Fengycin biosynthetic gene cluster (86% of genes show similarity) | 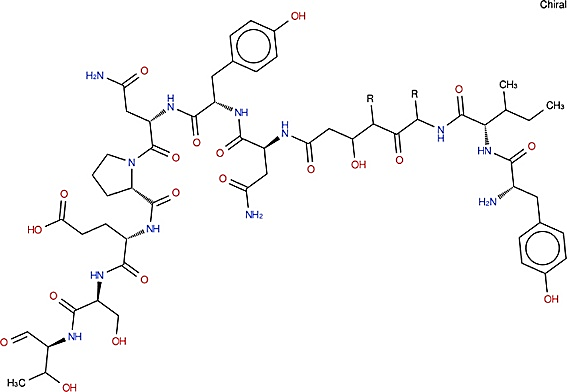 |
| Cluster 17 | Transatpks | 24376 | Difficidin biosynthetic gene cluster (26% of genes show similarity) | 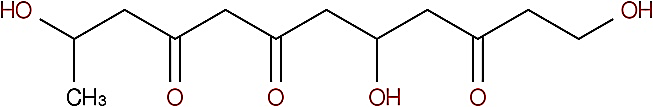 |
| Cluster 18 | Nrps | 14533 | Bacillibactin biosynthetic gene cluster (38% of genes show similarity) | 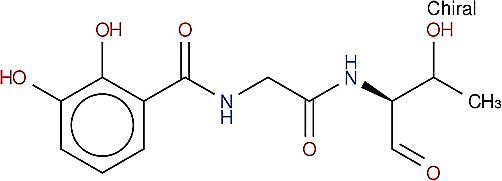 |
| Cluster 19 | Nrps | 13974 | Fengycin biosynthetic gene cluster (26% of genes show similarity) | 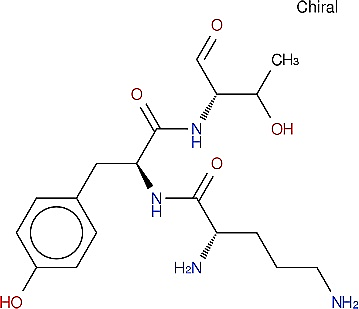 |

Table S1. Continued.

| Strain clusters | Type | Length (bp) | Most similar known clusters | Predicted core clusters |
| --- | --- | --- | --- | --- |
| **12B** | | | | |
| Cluster 20 | Nrps | 11772 | Fengycin biosynthetic gene cluster (20% of genes show similarity) | 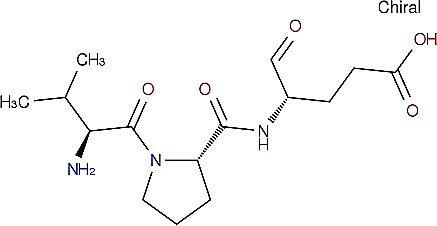 |
| Cluster 21 | Bacteriocin | 11445 | - | - |
| Cluster 22 | Siderophore | 11014 | - | - |
| Cluster 23 | Lantipeptide | 10806 | - | - |
| Cluster 24 | Nrps | 9962 | Surfactin biosynthetic gene cluster (8% of genes show similarity) | 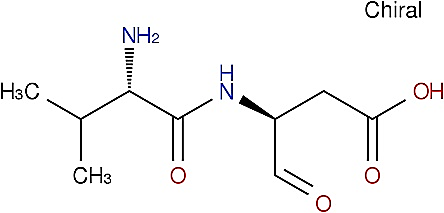 |
| Cluster 25 | Siderophore | 8277 | - | - |
| Cluster 26 | Terpene | 8021 | - | - |
| Cluster 27 | Other | 7340 | Surfactin biosynthetic gene cluster (30% of genes show similarity) | - |
| Cluster 28 | Nrps | 4669 | - | - |
| Cluster 29 | Nrps | 4634 | Fengycin biosynthetic gene cluster (13% of genes show similarity) | - |
| Cluster 30 | Nrps | 4288 | - | - |
| Cluster 31 | Other | 4165 | - | - |
| Cluster 32 | T3pks | 4095 | - | - |
| Cluster 33 | Nrps | 3986 | Bacitracin biosynthetic gene cluster (44% of genes show similarity) | - |
| Cluster 34 | Nrps | 3908 | - | - |
| Cluster 35 | Nrps | 3553 | Plipastatin biosynthetic gene cluster (23% of genes show similarity) | - |
| Cluster 36 | Nrps | 3314 | - | - |
| Cluster 37 | Nrps | 3257 | - | - |

Table S1. Continued.

| Strain clusters | Type | Length (bp) | Most similar known clusters | Predicted core clusters |
| --- | --- | --- | --- | --- |
| **12B** | | | | |
| Cluster 38 | Nrps | 2994 | - | - |
| Cluster 39 | Nrps | 2942 | Bacitracin biosynthetic gene cluster (33% of genes show similarity) | 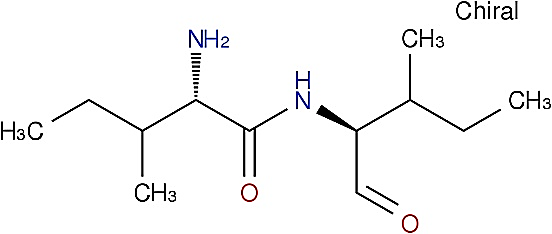 |
| Cluster 40 | Nrps | 2924 | - | - |
| Cluster 41 | Nrps | 2890 | Lichenysin biosynthetic gene cluster (14% of genes show similarity) | - |
| Cluster 42 | Nrps | 2344 | - | - |
| Cluster 43 | Nrps | 2320 | - | - |
| Cluster 44 | Nrps | 2289 | - | - |
| Cluster 45 | Nrps | 2168 | - | - |
| Cluster 46 | Nrps | 2089 | - | - |
| Cluster 47 | Nrps | 1796 | - | - |
| Cluster 48 | Nrps | 1550 | - | - |
| Cluster 49 | Nrps | 1452 | - | - |
| Cluster 50 | Nrps | 1261 | - | - |

Table S1. Continued.

| Strain clusters | Type | Length (bp) | Most similar known clusters | Predicted core clusters |
| --- | --- | --- | --- | --- |
| **MBE1283** | | | | |
| Cluster 1 | Transatpks | 85890 | Macrolactin biosynthetic gene cluster (100% of genes show similarity) | - |
| Cluster 2 | Nrps-Transatpks | 102698 | Bacillaene biosynthetic gene cluster (100% of genes show similarity) | 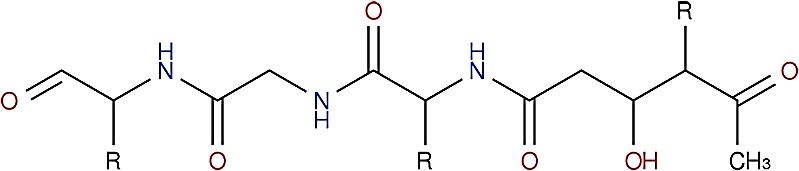 |
| Cluster 3 | Nrps-Transatpks | 137778 | Fengycin biosynthetic gene cluster (100% of genes show similarity) | 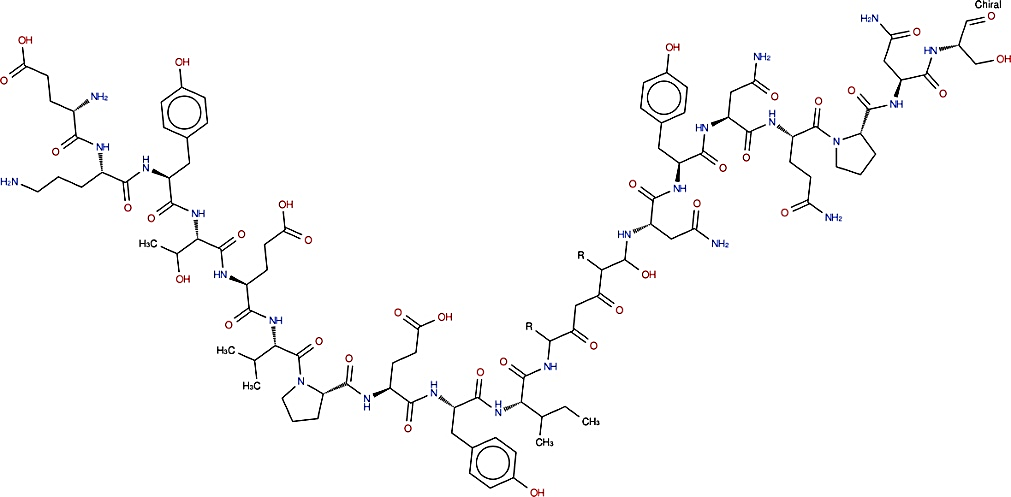 |
| Cluster 4 | Bacteriocin | 10824 | - | - |
| Cluster 5 | Terpene | 21883 | - | - |
| Cluster 6 | T3pks | 41109 | - | - |
| Cluster 7 | Transatpks | 100429 | Difficidin biosynthetic gene cluster (100% of genes show similarity) | 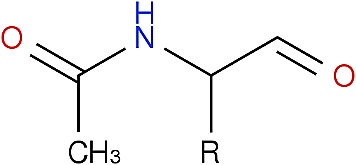 |
| Cluster 8 | Microcin | 20148 | - | - |
| Cluster 9 | Nrps-Bacteriocin | 66793 | Bacillibactin biosynthetic gene cluster (100% of genes show similarity) | 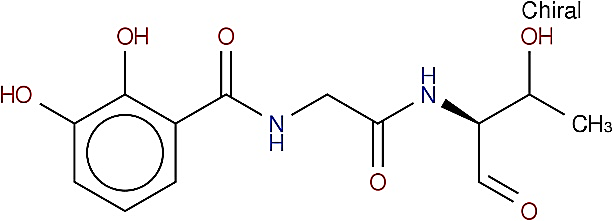 |

Table S1. Continued.

| Strain clusters | Type | Length (bp) | Most similar known clusters | Predicted core clusters |
| --- | --- | --- | --- | --- |
| **MBE1283** | | | | |
| Cluster 10 | Other | 41418 | Bacilysin biosynthetic gene cluster (100% of genes show similarity) | - |
| Cluster 11 | Lantipeptide | 23983 | Mersacidin biosynthetic gene cluster (90% of genes show similarity) | - |
| Cluster 12 | Microcin | 20148 | - | - |
| Cluster 13 | Microcin | 20148 | - | - |
| Cluster 14 | Microcin-Lantipeptide | 38412 | - | - |
| Cluster 15 | [Microcin](http://antismash.secondarymetabolites.org/help#microcin) | 26039 | - | - |
| Cluster 16 | [Nrps](http://antismash.secondarymetabolites.org/help#nrps) | 65407 | Surfactin biosynthetic gene cluster (82% of genes show similarity) | 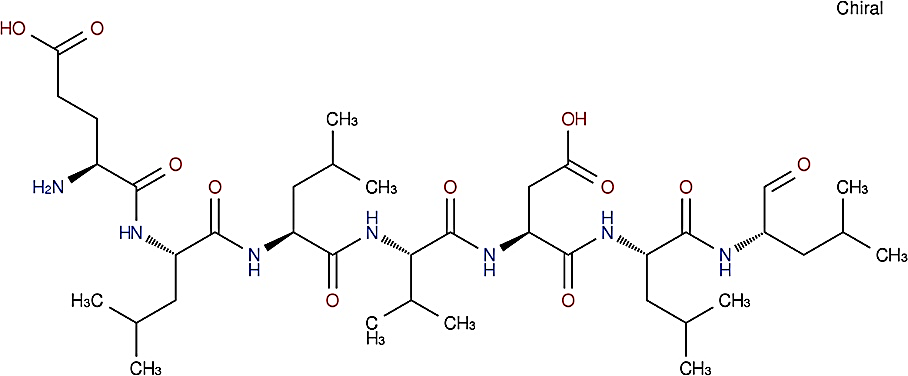 |
| Cluster 17 | [Microcin](http://antismash.secondarymetabolites.org/help#microcin) | 20148 | - | - |
| Cluster 18 | [Ladderane](http://antismash.secondarymetabolites.org/help#ladderane) | 41217 | - | - |
| Cluster 19 | [Microcin](http://antismash.secondarymetabolites.org/help#microcin) | 20148 | - | - |
| Cluster 20 | Otherks | 41244 | - | - |
| Cluster 21 | [Terpene](http://antismash.secondarymetabolites.org/help#terpene) | 20740 | - | - |

Table S1. Continued.

| Strain clusters | Type | Length (bp) | Most similar known clusters | Predicted core clusters |
| --- | --- | --- | --- | --- |
| **LFB112** | | | | |
| Cluster 1 | [Microcin](http://antismash.secondarymetabolites.org/help#microcin) | 20148 | - | - |
| Cluster 2 | [Microcin](http://antismash.secondarymetabolites.org/help#microcin) | 20148 | - | - |
| Cluster 3 | [Microcin](http://antismash.secondarymetabolites.org/help#microcin) | 25639 | - | - |
| Cluster 4 | [Microcin](http://antismash.secondarymetabolites.org/help#microcin) | 31866 | - | - |
| Cluster 5 | [Nrps](http://antismash.secondarymetabolites.org/help#nrps)-[Transatpks](http://antismash.secondarymetabolites.org/help#transatpks) | 70120 | Locillomycin biosynthetic gene cluster (35% of genes show similarity) | 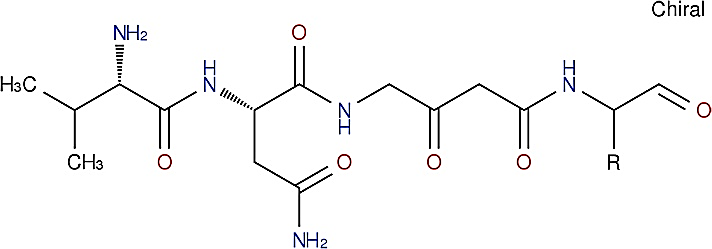 |
| Cluster 6 | [Nrps](http://antismash.secondarymetabolites.org/help#nrps) | 65405 | Surfactin biosynthetic gene cluster (78% of genes show similarity) | 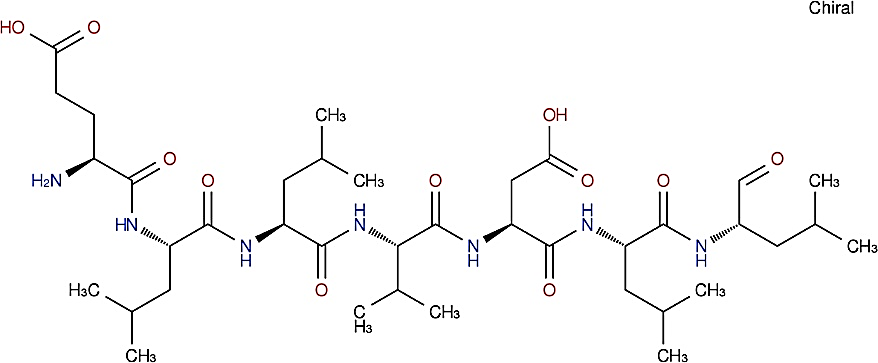 |
| Cluster 7 | [Microcin](http://antismash.secondarymetabolites.org/help#microcin) | 20148 | - | - |
| Cluster 8 | [Microcin](http://antismash.secondarymetabolites.org/help#microcin) | 20148 | - | - |
| Cluster 9 | [Otherks](http://antismash.secondarymetabolites.org/help#otherks) | 41244 | - | - |
| Cluster 10 | Terpene | 20740 | - | - |
| Cluster 11 | [Bacteriocin](http://antismash.secondarymetabolites.org/help#bacteriocin) | 10151 | - | - |
| Cluster 12 | Lantipeptide | 28889 | - | - |
| Cluster 13 | Transatpks | 85887 | Macrolactin biosynthetic gene cluster (100% of genes show similarity) | - |
| Cluster 14 | [Nrps](http://antismash.secondarymetabolites.org/help#nrps)-[Transatpks](http://antismash.secondarymetabolites.org/help#transatpks) | 102704 | Bacillaene biosynthetic gene cluster (100% of genes show similarity) | 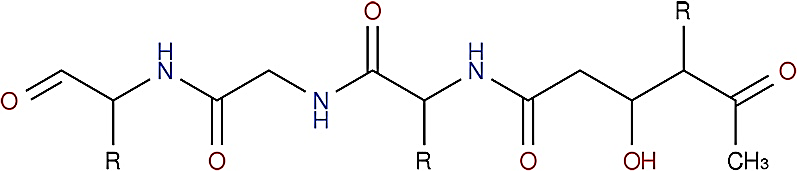 |

Table S1. Continued.

| Strain clusters | Type | Length (bp) | Most similar known clusters | Predicted core clusters |
| --- | --- | --- | --- | --- |
| **LFB112** | | | | |
| Cluster 15 | Nrps-Transatpks | 141738 | Fengycin biosynthetic gene cluster (100% of genes show similarity) | 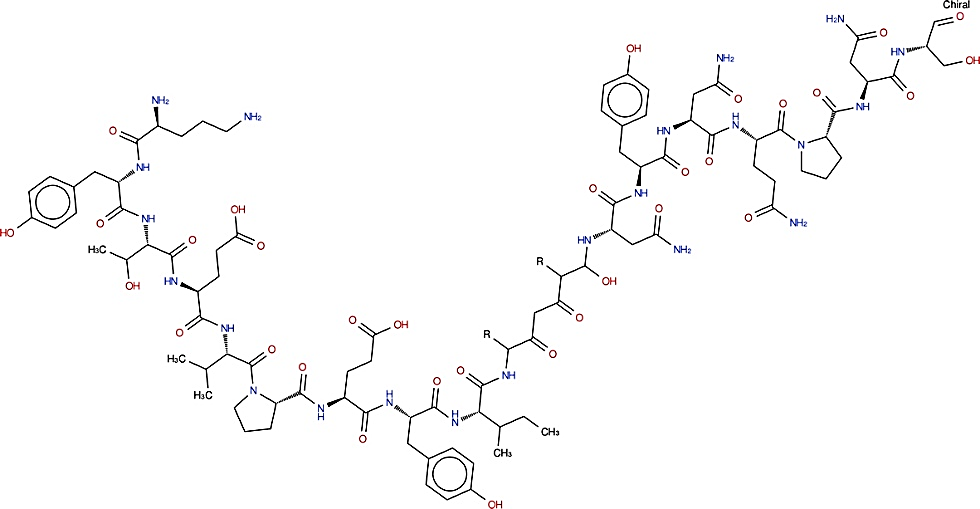 |
| Cluster 16 | [Terpene](http://antismash.secondarymetabolites.org/help#terpene) | 21883 | - | - |
| Cluster 17 | T3pks | 41109 | - | - |
| Cluster 18 | Transatpks | 100438 | Difficidin biosynthetic gene cluster (100% of genes show similarity) | 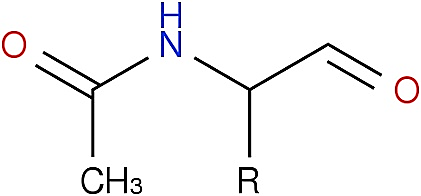 |
| Cluster 19 | Microcin | 20148 | - | - |
| Cluster 20 | Nrps-Bacteriocin | 66792 | Bacillibactin biosynthetic gene cluster (100% of genes show similarity) | 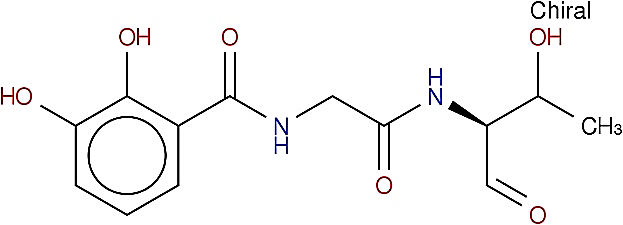 |
| Cluster 21 | Other | 41418 | Bacilysin biosynthetic gene cluster (100% of genes show similarity) | - |

Table S1. Continued.

| Strain clusters | Type | Length (bp) | Most similar known clusters | Predicted core clusters |
| --- | --- | --- | --- | --- |
| **B15** | | | | |
| Cluster 1 | Microcin | 20148 | - | - |
| Cluster 2 | Microcin | 20148 | - | - |
| Cluster 3 | Microcin | 26032 | - | - |
| Cluster 4 | Microcin | 30414 | - | - |
| Cluster 5 | Nrps | 65772 | Surfactin biosynthetic gene cluster (91% of genes show similarity) | 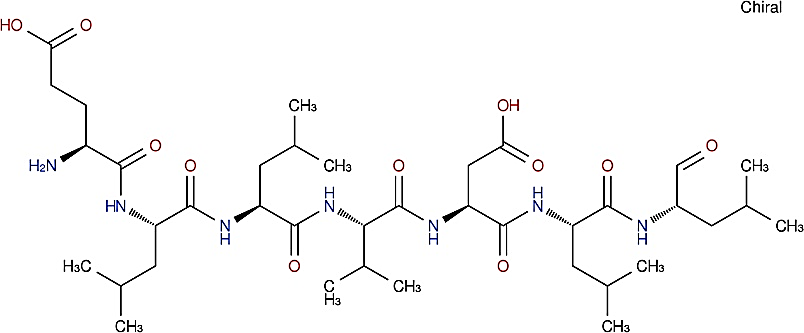 |
| Cluster 6 | Otherks | 41244 | - | - |
| Cluster 7 | Terpene | 20740 | - | - |
| Cluster 8 | Transatpks | 85893 | Macrolactin biosynthetic gene cluster (100% of genes show similarity) | - |
| Cluster 9 | Nrps-Transatpks | 102671 | Bacillaene biosynthetic gene cluster (100% of genes show similarity) | 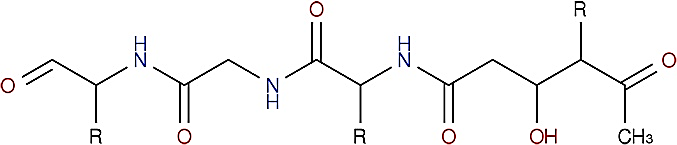 |
| Cluster 10 | Bacteriocin-Nrps-Transatpks | 136016 | Fengycin biosynthetic gene cluster (100% of genes show similarity) | 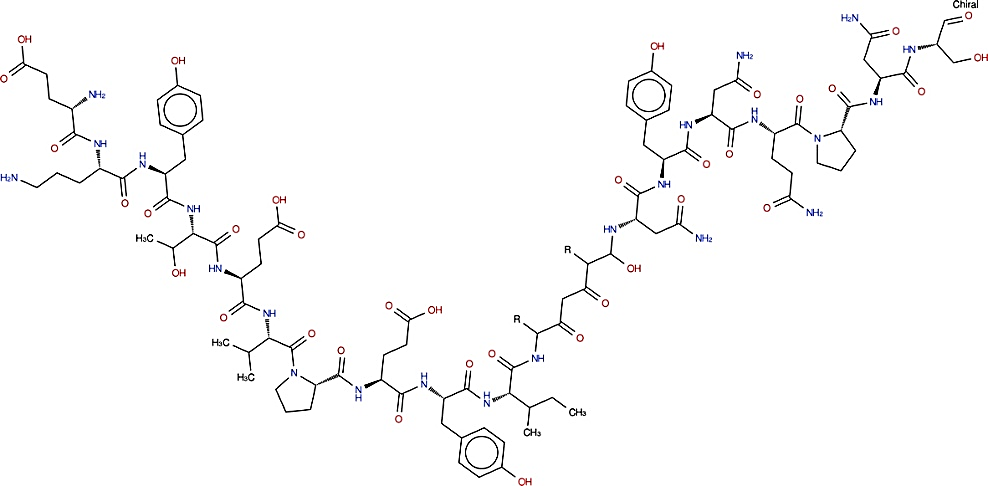 |

Table S1. Continued.

| Strain clusters | Type | Length (bp) | Most similar known clusters | Predicted core clusters |
| --- | --- | --- | --- | --- |
| **B15** | | | | |
| Cluster 11 | Terpene | 21883 | - | - |
| Cluster 12 | T3pks | 41109 | - | - |
| Cluster 13 | Transatpks | 100644 | Difficidin biosynthetic gene cluster (100% of genes show similarity) | 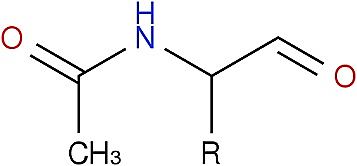 |
| Cluster 14 | Microcin | 20148 | - | - |
| Cluster 15 | Nrps-Bacteriocin | 66791 | Bacillibactin biosynthetic gene cluster (100% of genes show similarity) | 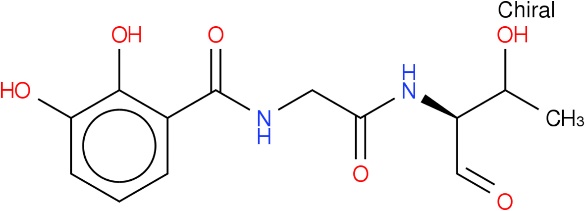 |
| Cluster 16 | Nrps | 57393 | - | - |
| Cluster 17 | Other | 41418 | Bacilysin biosynthetic gene cluster (100% of genes show similarity) | - |

Table S1. Continued.

| Strain clusters | Type | Length (bp) | Most similar known clusters | Predicted core clusters |
| --- | --- | --- | --- | --- |
| **L-H15** | | | | |
| Cluster 1 | [Microcin](http://antismash.secondarymetabolites.org/help#microcin) | 20148 | - | - |
| Cluster 2 | [Microcin](http://antismash.secondarymetabolites.org/help#microcin) | 26033 | - | - |
| Cluster 3 | [Microcin](http://antismash.secondarymetabolites.org/help#microcin) | 30420 | - | - |
| Cluster 4 | Nrps | 65407 | Surfactin biosynthetic gene cluster (82% of genes show similarity) | 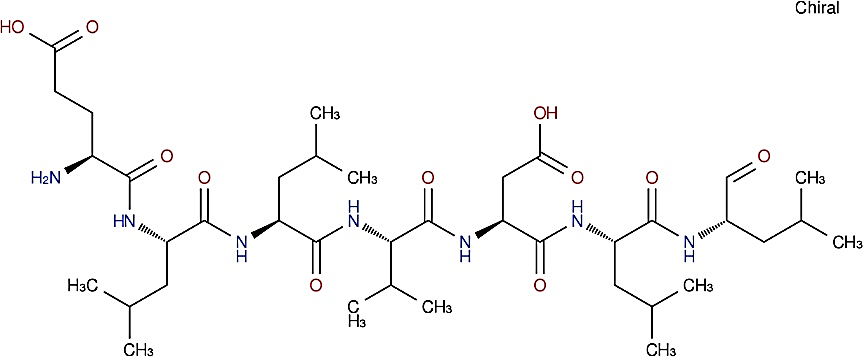 |
| Cluster 5 | [Microcin](http://antismash.secondarymetabolites.org/help#microcin) | 20148 | - | - |
| Cluster 6 | [Microcin](http://antismash.secondarymetabolites.org/help#microcin) | 20148 | - | - |
| Cluster 7 | Otherks | 41244 | - | - |
| Cluster 8 | Terpene | 20740 | - | - |
| Cluster 9 | Lantipeptide | 28889 | - | - |
| Cluster 10 | Transatpks | 85884 | Macrolactin biosynthetic gene cluster (100% of genes show similarity) | - |
| Cluster 11 | Nrps-Transatpks | 102704 | Bacillaene biosynthetic gene cluster (100% of genes show similarity) | 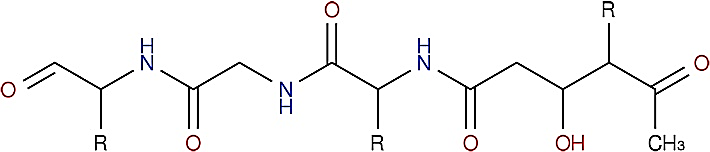 |
| Cluster 12 | Nrps-Transatpks | 137811 | Fengycin biosynthetic gene cluster (100% of genes show similarity) | 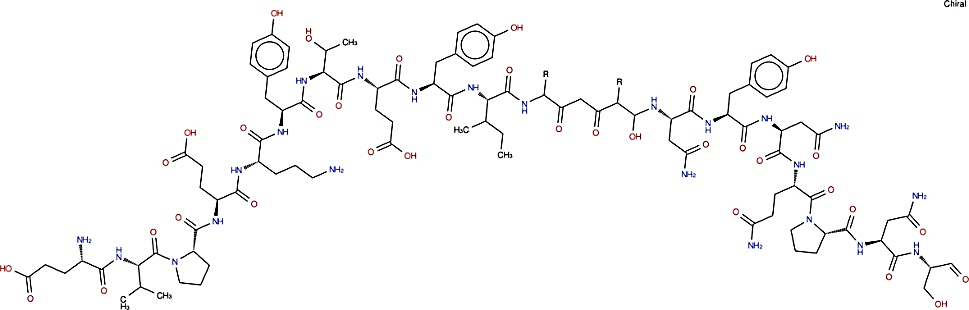 |
| Cluster 13 | Terpene | 21883 | - | - |
| Cluster 14 | T3pks | 41109 | - | - |

Table S1. Continued.

| Strain clusters | Type | Length (bp) | Most similar known clusters | Predicted core clusters |
| --- | --- | --- | --- | --- |
| **L-H15** | | | | |
| Cluster 15 | Transatpks | 100459 | Difficidin biosynthetic gene cluster (100% of genes show similarity) | 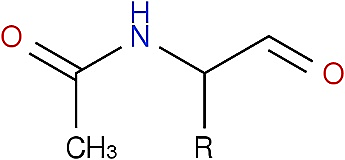 |
| Cluster 16 | Microcin | 20148 | - | - |
| Cluster 17 | [Nrps](http://antismash.secondarymetabolites.org/help#nrps)-[Bacteriocin](http://antismash.secondarymetabolites.org/help#bacteriocin) | 66792 | Bacillibactin biosynthetic gene cluster (100% of genes show similarity) | 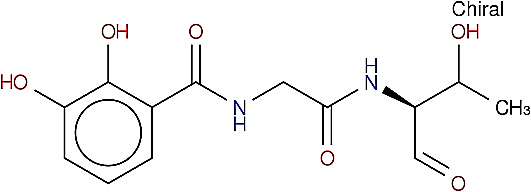 |
| Cluster 18 | [Other](http://antismash.secondarymetabolites.org/help#other) | 41418 | Bacilysin biosynthetic gene cluster (100% of genes show similarity) | - |

Table S1. Continued.

| Strain clusters | Type | Length (bp) | Most similar known clusters | Predicted core clusters |
| --- | --- | --- | --- | --- |
| **DSM7** | | | | |
| Cluster 1 | [Microcin](http://antismash.secondarymetabolites.org/help#microcin) | 20148 | - | - |
| Cluster 2 | [Microcin](http://antismash.secondarymetabolites.org/help#microcin) | 20148 | - | - |
| Cluster 3 | [Microcin](http://antismash.secondarymetabolites.org/help#microcin) | 26035 | - | - |
| Cluster 4 | [Microcin](http://antismash.secondarymetabolites.org/help#microcin) | 31413 | - | - |
| Cluster 5 | Nrps | 65410 | Surfactin biosynthetic gene cluster (82% of genes show similarity) | 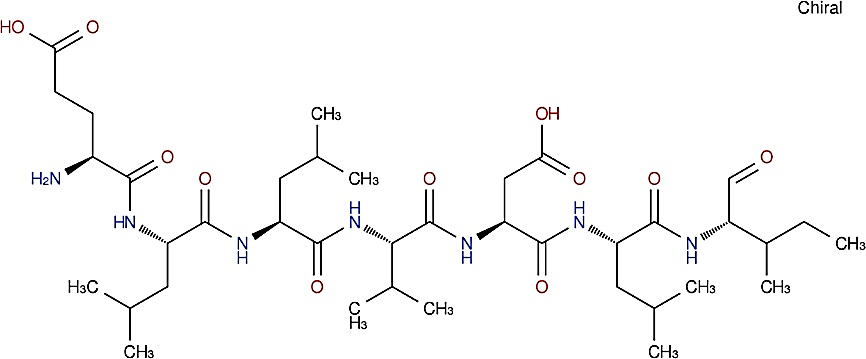 |
| Cluster 6 | [Microcin](http://antismash.secondarymetabolites.org/help#microcin) | 20148 | - | - |
| Cluster 7 | [Lantipeptide](http://antismash.secondarymetabolites.org/help#lantipeptide) | 22597 | - | - |
| Cluster 8 | [Microcin](http://antismash.secondarymetabolites.org/help#microcin) | 20148 | - | - |
| Cluster 9 | [Otherks](http://antismash.secondarymetabolites.org/help#otherks) | 41244 | - | - |
| Cluster 10 | [Terpene](http://antismash.secondarymetabolites.org/help#terpene) | 20743 | - | - |
| Cluster 11 | [Nrps](http://antismash.secondarymetabolites.org/help#nrps)-[Transatpks](http://antismash.secondarymetabolites.org/help#transatpks) | 102704 | Bacillaene biosynthetic gene cluster (100% of genes show similarity) | 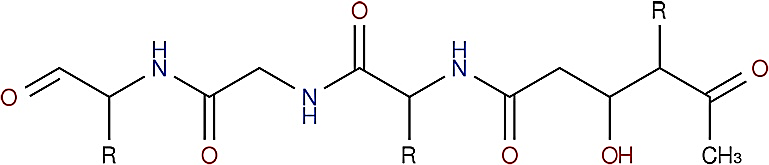 |
| Cluster 12 | [Nrps](http://antismash.secondarymetabolites.org/help#nrps)-[Transatpks](http://antismash.secondarymetabolites.org/help#transatpks) | 110421 | Fengycin biosynthetic gene cluster (93% of genes show similarity) | 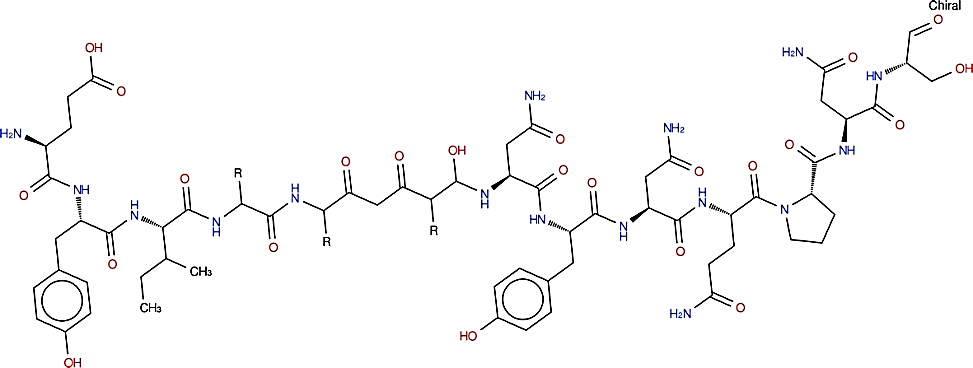 |
| Cluster 13 | Terpene | 21883 | - | - |

Table S1. Continued.

| Strain clusters | Type | Length (bp) | Most similar known clusters | Predicted core clusters |
| --- | --- | --- | --- | --- |
| **DSM7** | | | | |
| Cluster 14 | [T3pks](http://antismash.secondarymetabolites.org/help#t3pks) | 41109 | - | - |
| Cluster 15 | [Nrps](http://antismash.secondarymetabolites.org/help#nrps) | 51633 | - | - |
| Cluster 16 | [Microcin](http://antismash.secondarymetabolites.org/help#microcin) | 20148 | - | - |
| Cluster 17 | [Nrps](http://antismash.secondarymetabolites.org/help#nrps)-[Bacteriocin](http://antismash.secondarymetabolites.org/help#bacteriocin) | 66735 | Bacillibactin biosynthetic gene cluster (100% of genes show similarity) | 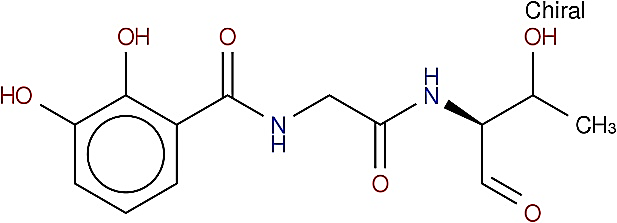 |
| Cluster 18 | [Other](http://antismash.secondarymetabolites.org/help#other) | 41454 | Bacilysin biosynthetic gene cluster (100% of genes show similarity) | - |

Table S1. Continued.

| Strain clusters | Type | Length (bp) | Most similar known clusters | Predicted core clusters |
| --- | --- | --- | --- | --- |
| **CECT 8238** | | | | |
| Cluster 1 | Nrps | 68420 | - | - |
| Cluster 2 | [Other](http://antismash.secondarymetabolites.org/help#other) | 41418 | Bacilysin biosynthetic gene cluster (100% of genes show similarity) | - |
| Cluster 3 | [Microcin](http://antismash.secondarymetabolites.org/help#microcin) | 20148 | - | - |
| Cluster 4 | [Microcin](http://antismash.secondarymetabolites.org/help#microcin) | 20148 | - | - |
| Cluster 5 | [Microcin](http://antismash.secondarymetabolites.org/help#microcin) | 25974 | - | - |
| Cluster 6 | [Microcin](http://antismash.secondarymetabolites.org/help#microcin) | 26091 | - | - |
| Cluster 7 | [Nrps](http://antismash.secondarymetabolites.org/help#nrps) | 65407 | Surfactin biosynthetic gene cluster (82% of genes show similarity) | 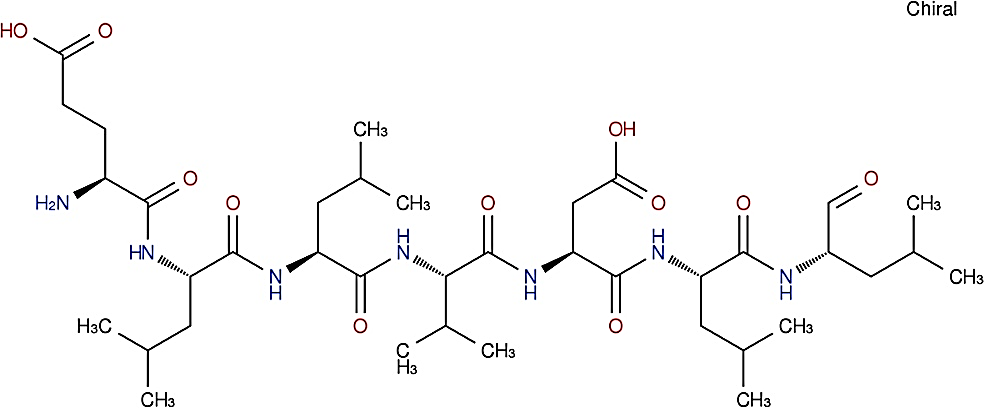 |
| Cluster 8 | [Microcin](http://antismash.secondarymetabolites.org/help#microcin) | 20148 | - | - |
| Cluster 9 | [Phosphonate](http://antismash.secondarymetabolites.org/help#phosphonate) | 40902 | Pactamycin biosynthetic gene cluster (3% of genes show similarity) |  |
| Cluster 10 | Microcin | 20148 | - | - |
| Cluster 11 | [Otherks](http://antismash.secondarymetabolites.org/help#otherks) | 41244 | - | - |
| Cluster 12 | Terpene | 20773 | - | - |
| Cluster 13 | [Transatpks](http://antismash.secondarymetabolites.org/help#transatpks) | 85893 | Macrolactin biosynthetic gene cluster (100% of genes show similarity) | - |
| Cluster 14 | [Nrps](http://antismash.secondarymetabolites.org/help#nrps)-[Transatpks](http://antismash.secondarymetabolites.org/help#transatpks) | 102676 | Bacillaene biosynthetic gene cluster (100% of genes show similarity) | 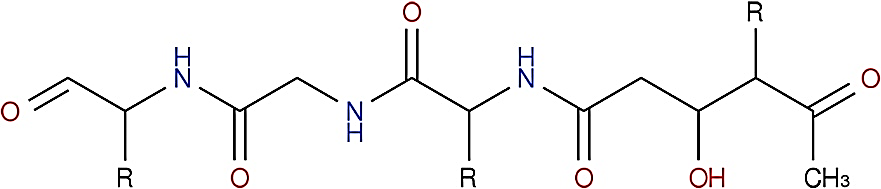 |

Table S1. Continued.

| Strain clusters | Type | Length (bp) | Most similar known clusters | Predicted core clusters |
| --- | --- | --- | --- | --- |
| **CECT 8238** | | | | |
| Cluster 15 | [Nrps](http://antismash.secondarymetabolites.org/help#nrps)-[Transatpks](http://antismash.secondarymetabolites.org/help#transatpks) | 137820 | Fengycin biosynthetic gene cluster (100% of genes show similarity) | 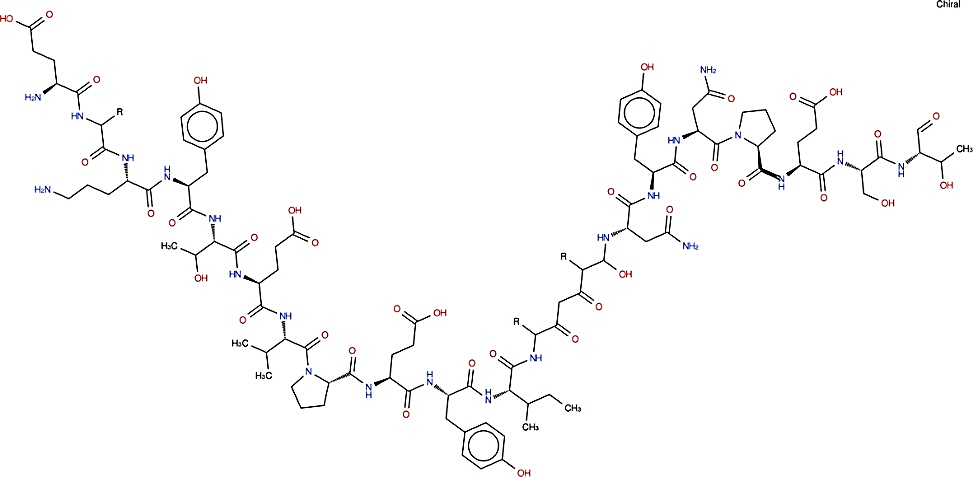 |
| Cluster 16 | [Terpene](http://antismash.secondarymetabolites.org/help#terpene) | 21883 | - | - |
| Cluster 17 | [T3pks](http://antismash.secondarymetabolites.org/help#t3pks) | 41109 | - | - |
| Cluster 18 | [Transatpks](http://antismash.secondarymetabolites.org/help#transatpks) | 100441 | Difficidin biosynthetic gene cluster (100% of genes show similarity) | 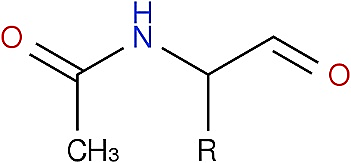 |
| Cluster 19 | [Microcin](http://antismash.secondarymetabolites.org/help#microcin) | 20148 | - | - |
| Cluster 20 | [Nrps](http://antismash.secondarymetabolites.org/help#nrps)-[Bacteriocin](http://antismash.secondarymetabolites.org/help#bacteriocin) | 66796 | Bacillibactin biosynthetic gene cluster (100% of genes show similarity) | 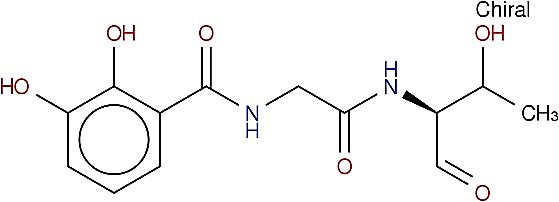 |

Table S1. Continued.

| Strain clusters | Type | Length (bp) | Most similar known clusters | Predicted core clusters |
| --- | --- | --- | --- | --- |
| **L-S60** | | | | |
| Cluster 1 | [Microcin](http://antismash.secondarymetabolites.org/help#microcin) | 20148 | - | - |
| Cluster 2 | [Microcin](http://antismash.secondarymetabolites.org/help#microcin) | 26033 | - | - |
| Cluster 3 | [Microcin](http://antismash.secondarymetabolites.org/help#microcin) | 137661 | - | - |
| Cluster 4 | [Nrps](http://antismash.secondarymetabolites.org/help#nrps) | 65407 | Surfactin biosynthetic gene cluster (82% of genes show similarity) | 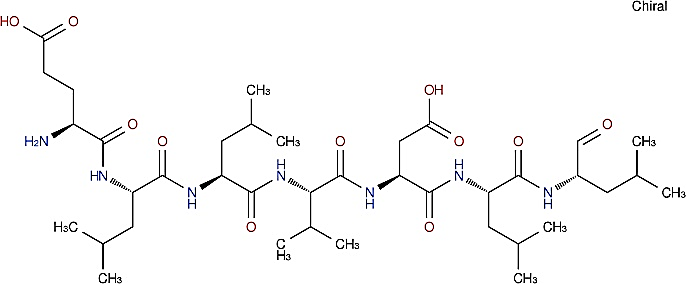 |
| Cluster 5 | [Microcin](http://antismash.secondarymetabolites.org/help#microcin) | 20148 | - | - |
| Cluster 6 | [Microcin](http://antismash.secondarymetabolites.org/help#microcin) | 20148 | - | - |
| Cluster 7 | [Otherks](http://antismash.secondarymetabolites.org/help#otherks) | 41244 | - | - |
| Cluster 8 | [Terpene](http://antismash.secondarymetabolites.org/help#terpene) | 20740 | - | - |
| Cluster 9 | [Lantipeptide](http://antismash.secondarymetabolites.org/help#lantipeptide) | 28889 | - | - |
| Cluster 10 | [Transatpks](http://antismash.secondarymetabolites.org/help#transatpks) | 85884 | Macrolactin biosynthetic gene cluster (100% of genes show similarity) | - |
| Cluster 11 | Nrps-[Transatpks](http://antismash.secondarymetabolites.org/help#transatpks) | 102704 | Bacillaene biosynthetic gene cluster (100% of genes show similarity) | 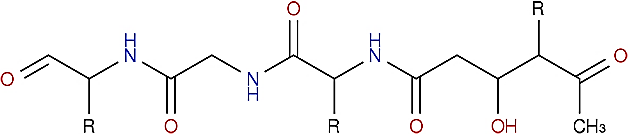 |
| Cluster 12 | Nrps-[Transatpks](http://antismash.secondarymetabolites.org/help#transatpks) | 155426 | Fengycin biosynthetic gene cluster (100% of genes show similarity) | 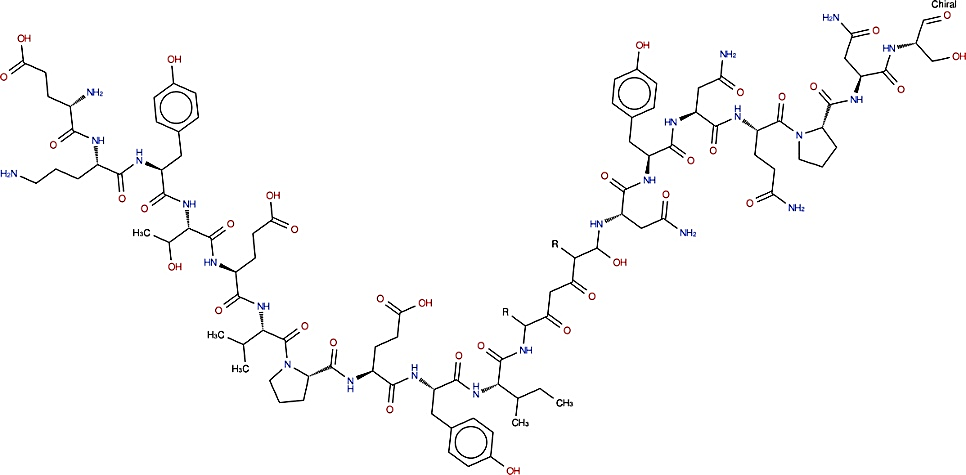 |

Table S1. Continued.

| Strain clusters | Type | Length (bp) | Most similar known clusters | Predicted core clusters |
| --- | --- | --- | --- | --- |
| **L-S60** | | | | |
| Cluster 13 | [Terpene](http://antismash.secondarymetabolites.org/help#terpene) | 21883 | - | - |
| Cluster 14 | [T3pks](http://antismash.secondarymetabolites.org/help#t3pks) | 41109 | - | - |
| Cluster 15 | [Transatpks](http://antismash.secondarymetabolites.org/help#transatpks) | 100459 | Difficidin biosynthetic gene cluster (100% of genes show similarity) | 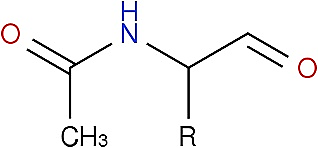 |
| Cluster 16 | [Microcin](http://antismash.secondarymetabolites.org/help#microcin) | 20148 | - | - |
| Cluster 17 | [Nrps](http://antismash.secondarymetabolites.org/help#nrps)-[Bacteriocin](http://antismash.secondarymetabolites.org/help#bacteriocin) | 66792 | Bacillibactin biosynthetic gene cluster (100% of genes show similarity) | 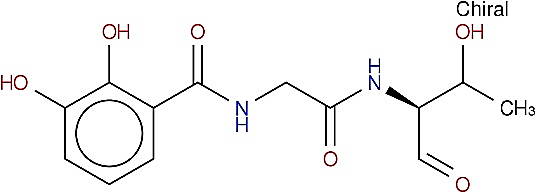 |
| Cluster 18 | Others | 41418 | Bacilysin biosynthetic gene cluster (100% of genes show similarity) |  |

Table S1. Continued.

| Strain clusters | Type | Length (bp) | Most similar known clusters | Predicted core clusters |
| --- | --- | --- | --- | --- |
| **IT-45** |  |  |  |  |
| Cluster 1 | Others | 41418 | Bacilysin biosynthetic gene cluster (100% of genes show similarity) | - |
| Cluster 2 | [Bacteriocin](http://antismash.secondarymetabolites.org/help#bacteriocin)-[Nrps](http://antismash.secondarymetabolites.org/help#nrps) | 51793 | Bacillibactin biosynthetic gene cluster (92% of genes show similarity) | 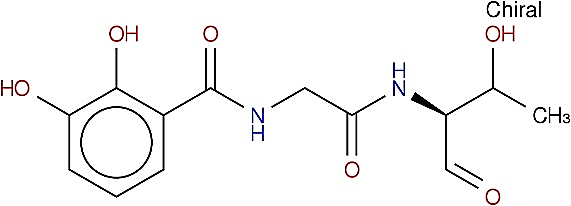 |
| Cluster 3 | [Microcin](http://antismash.secondarymetabolites.org/help#microcin) | 20148 | - | - |
| Cluster 4 | [Transatpks](http://antismash.secondarymetabolites.org/help#transatpks) | 100438 | Difficidin biosynthetic gene cluster (93% of genes show similarity) | 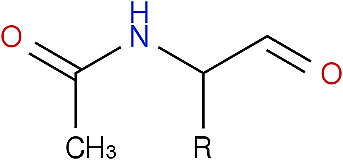 |
| Cluster 5 | T3pks | 41109 | - | - |
| Cluster 6 | Terpene | 21883 | - | - |
| Cluster 7 | Nrps-Transatpks | 137829 | Fengycin biosynthetic gene cluster (100% of genes show similarity) | 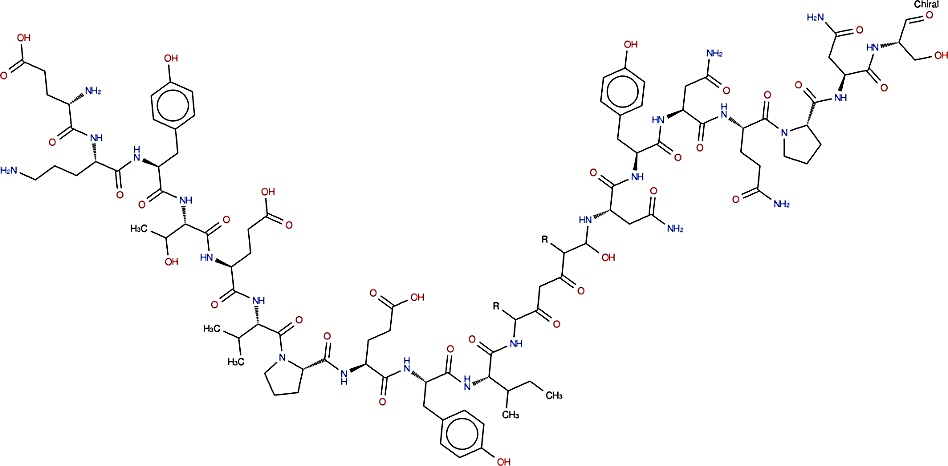 |
| Cluster 8 | [Transatpks](http://antismash.secondarymetabolites.org/help#transatpks)-[Nrps](http://antismash.secondarymetabolites.org/help#nrps) | 102701 | Bacillaene biosynthetic gene cluster (92% of genes show similarity) | 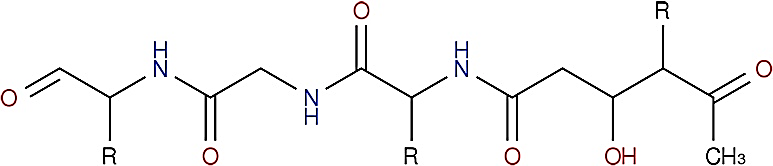 |

Table S1. Continued.

| Strain clusters | Type | Length (bp) | Most similar known clusters | Predicted core clusters |
| --- | --- | --- | --- | --- |
| **IT-45** | | | | |
| Cluster 9 | Transatpks | 85881 | Macrolactin biosynthetic gene cluster (90% of genes show similarity) | - |
| Cluster 10 | Lantipeptide | 28889 | - | - |
| Cluster 11 | Terpene | 20740 | - | - |
| Cluster 12 | Otherks | 41244 | Butirosin biosynthetic gene cluster (7% of genes show similarity) | - |
| Cluster 13 | [Microcin](http://antismash.secondarymetabolites.org/help#microcin) | 20148 | - | - |
| Cluster 14 | [Microcin](http://antismash.secondarymetabolites.org/help#microcin) | 20148 | - | - |
| Cluster 15 | Nrps | 54640 | Surfactin biosynthetic gene cluster (78% of genes show similarity) | 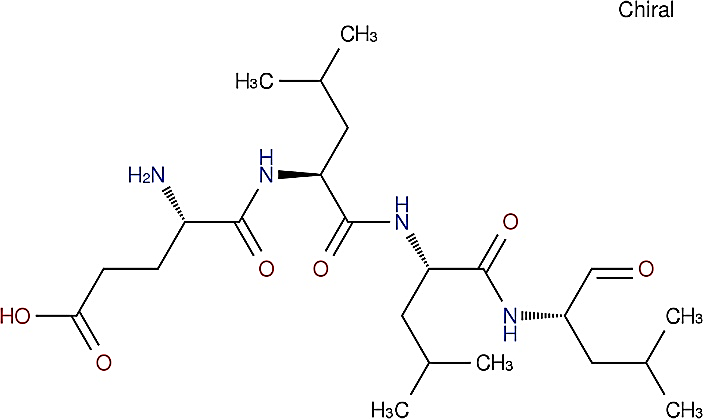 |
| Cluster 16 | [Microcin](http://antismash.secondarymetabolites.org/help#microcin) | 31866 | - | - |
| Cluster 17 | [Microcin](http://antismash.secondarymetabolites.org/help#microcin) | 25975 | - | - |
| Cluster 18 | [Microcin](http://antismash.secondarymetabolites.org/help#microcin) | 20148 | - | - |
| Cluster 19 | [Microcin](http://antismash.secondarymetabolites.org/help#microcin) | 20515 | - | - |

Table S1. Continued.

| Strain clusters | Type | Length (bp) | Most similar known clusters | Predicted core clusters |
| --- | --- | --- | --- | --- |
| **KHG19** | | | | |
| Cluster 1 | [Nrps](http://antismash.secondarymetabolites.org/help#nrps) | 65407 | Surfactin biosynthetic gene cluster (91% of genes show similarity) | 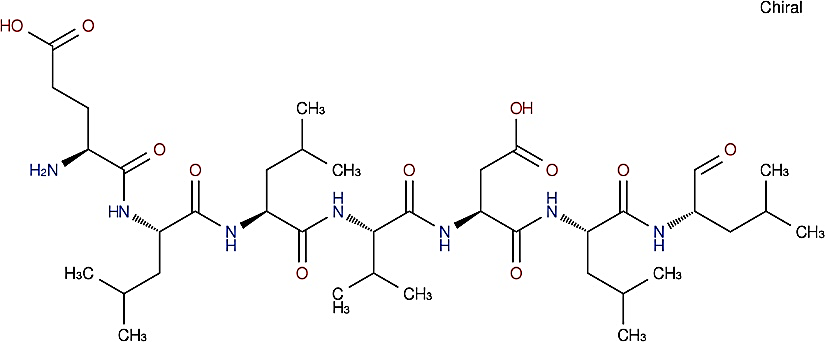 |
| Cluster 2 | [Microcin](http://antismash.secondarymetabolites.org/help#microcin) | 20148 | - | - |
| Cluster 3 | [Otherks](http://antismash.secondarymetabolites.org/help#otherks) | 41244 | - | - |
| Cluster 4 | [Terpene](http://antismash.secondarymetabolites.org/help#terpene) | 20740 | - | - |
| Cluster 5 | [Transatpks](http://antismash.secondarymetabolites.org/help#transatpks) | 85893 | - | - |
| Cluster 6 | Nrps-Transatpks | 102686 | Bacillaene biosynthetic gene cluster (100% of genes show similarity) | 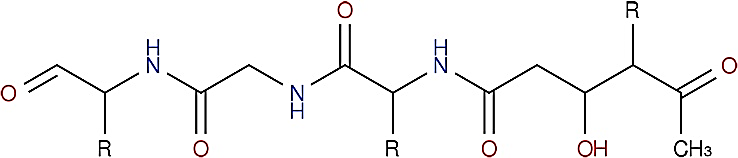 |
| Cluster 7 | Nrps-Transatpks | 137840 | Fengycin biosynthetic gene cluster (100% of genes show similarity) | 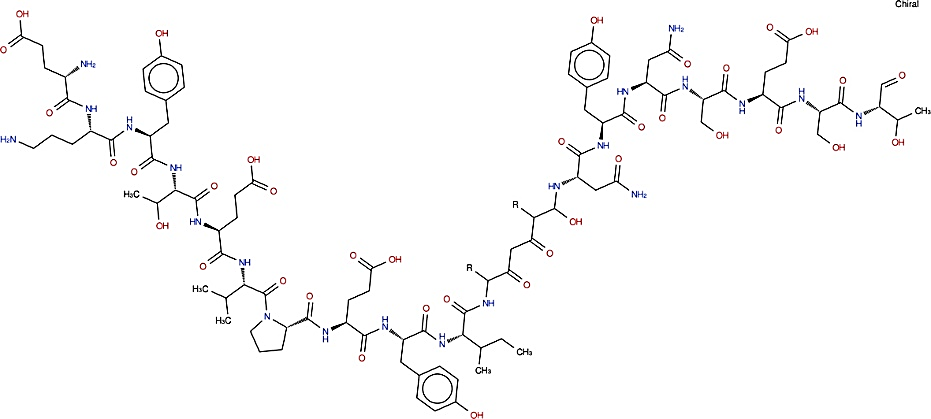 |
| Cluster 8 | [Terpene](http://antismash.secondarymetabolites.org/help#terpene) | 21883 | - | - |
| Cluster 9 | [T3pks](http://antismash.secondarymetabolites.org/help#t3pks) | 41109 | - | - |
| Cluster 10 | Transatpks | 98200 | Difficidin biosynthetic gene cluster (100% of genes show similarity) | 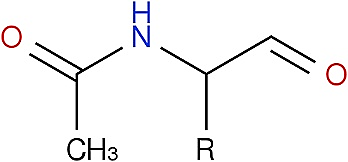 |

Table S1. Continued.

| Strain clusters | Type | Length (bp) | Most similar known clusters | Predicted core clusters |
| --- | --- | --- | --- | --- |
| **KHG19** | | | | |
| Cluster 11 | Microcin | 20148 | - | - |
| Cluster 12 | Nrps-Bacteriocin | 68082 | Bacillibactin biosynthetic gene cluster (100% of genes show similarity) | 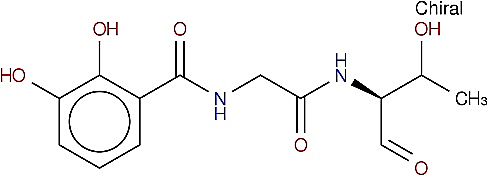 |
| Cluster 13 | [Nrps](http://antismash.secondarymetabolites.org/help#nrps) | 68420 | - | - |
| Cluster 14 | [Other](http://antismash.secondarymetabolites.org/help#other) | 41418 | Bacilysin biosynthetic gene cluster (100% of genes show similarity) | - |
| Cluster 15 | [Lantipeptide](http://antismash.secondarymetabolites.org/help#lantipeptide) | 23984 | Mersacidin biosynthetic gene cluster (90% of genes show similarity) | - |
| Cluster 16 | Microcin | 20148 | - | - |
| Cluster 17 | Microcin | 20148 | - | - |
| Cluster 18 | Microcin | 26034 | - | - |
| Cluster 19 | Microcin | 24483 | - | - |

Table S1. Continued.

| Strain clusters | Type | Length (bp) | Most similar known clusters | Predicted core clusters |
| --- | --- | --- | --- | --- |
| **Y2** | | | | |
| Cluster 1 | [Microcin](http://antismash.secondarymetabolites.org/help#microcin) | 20148 | - | - |
| Cluster 2 | [Microcin](http://antismash.secondarymetabolites.org/help#microcin) | 20148 | - | - |
| Cluster 3 | [Microcin](http://antismash.secondarymetabolites.org/help#microcin) | 25975 | - | - |
| Cluster 4 | [Microcin](http://antismash.secondarymetabolites.org/help#microcin) | 30420 | - | - |
| Cluster 5 | Nrps | 54640 | Surfactin biosynthetic gene cluster (82% of genes show similarity) | 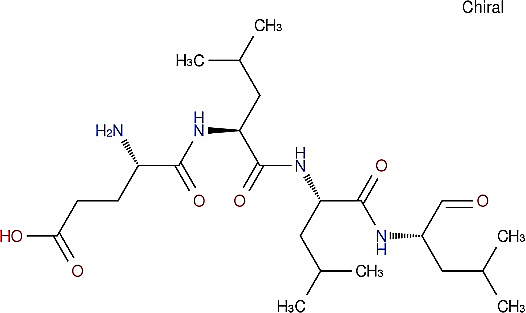 |
| Cluster 6 | [Microcin](http://antismash.secondarymetabolites.org/help#microcin) | 20148 | - | - |
| Cluster 7 | [Phosphonate](http://antismash.secondarymetabolites.org/help#phosphonate) | 40902 | Pactamycin biosynthetic gene cluster (3% of genes show similarity) | - |
| Cluster 8 | [Microcin](http://antismash.secondarymetabolites.org/help#microcin) | 20148 | - | - |
| Cluster 9 | [Otherks](http://antismash.secondarymetabolites.org/help#otherks) | 41244 | - | - |
| Cluster 10 | [Terpene](http://antismash.secondarymetabolites.org/help#terpene) | 20740 | - | - |
| Cluster 11 | [Transatpks](http://antismash.secondarymetabolites.org/help#transatpks) | 85902 | Macrolactin biosynthetic gene cluster (100% of genes show similarity) | - |
| Cluster 12 | [Nrps](http://antismash.secondarymetabolites.org/help#nrps)-[Transatpks](http://antismash.secondarymetabolites.org/help#transatpks) | 102680 | Bacillaene biosynthetic gene cluster (100% of genes show similarity) | 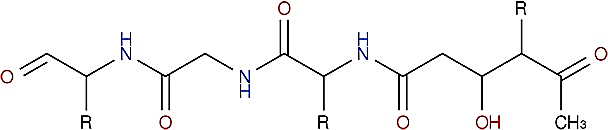 |
| Cluster 13 | [Nrps](http://antismash.secondarymetabolites.org/help#nrps)-[Transatpks](http://antismash.secondarymetabolites.org/help#transatpks) | 137833 | Fengycin biosynthetic gene cluster (100% of genes show similarity) | 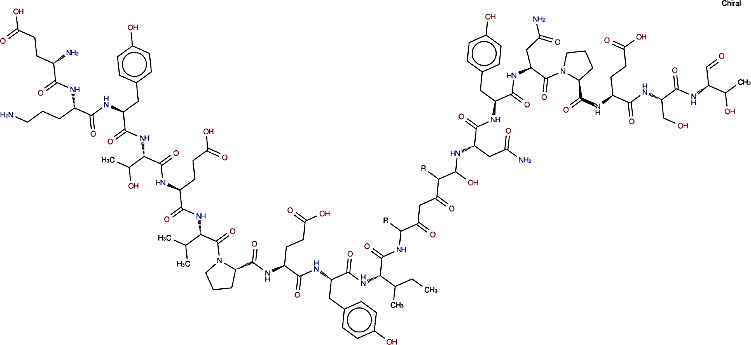 |

Table S1. Continued.

| Strain clusters | Type | Length (bp) | Most similar known clusters | Predicted core clusters |
| --- | --- | --- | --- | --- |
| **Y2** | | | | |
| Cluster 14 | [Terpene](http://antismash.secondarymetabolites.org/help#terpene) | 21883 | - | - |
| Cluster 15 | T3pks | 41109 | - | - |
| Cluster 16 | [Transatpks](http://antismash.secondarymetabolites.org/help#transatpks) | 100442 | Difficidin biosynthetic gene cluster (100% of genes show similarity) |  |
| Cluster 17 | [Microcin](http://antismash.secondarymetabolites.org/help#microcin) | 20148 | - | - |
| Cluster 18 | [Nrps](http://antismash.secondarymetabolites.org/help#nrps)-[Bacteriocin](http://antismash.secondarymetabolites.org/help#bacteriocin) | 66796 | Bacillibactin biosynthetic gene cluster (100% of genes show similarity) | 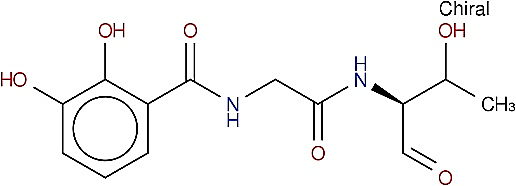 |
| Cluster 19 | [Nrps](http://antismash.secondarymetabolites.org/help#nrps) | 68420 | - | - |
| Cluster 20 | [Other](http://antismash.secondarymetabolites.org/help#other) | 41418 | Bacilysin biosynthetic gene cluster (100% of genes show similarity) | - |
| Cluster 21 | Lantipeptide | 23984 | Mersacidin biosynthetic gene cluster (90% of genes show similarity) | - |

Table S1. Continued.

| Strain clusters | Type | Length (bp) | Most similar known clusters | Predicted core clusters |
| --- | --- | --- | --- | --- |
| **CECT 8237** | | | | |
| Cluster 1 | [Transatpks](http://antismash.secondarymetabolites.org/help#transatpks) | 23738 | Bacillaene biosynthetic gene cluster (14% of genes show similarity) | - |
| Cluster 2 | Bacteriocin-Nrps-Transatpks | 136285 | Fengycin biosynthetic gene cluster (100% of genes show similarity) | 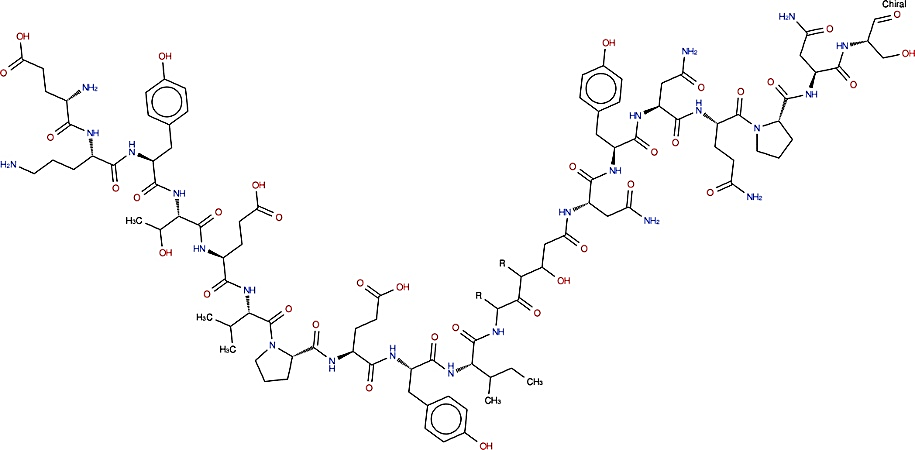 |
| Cluster 3 | [Terpene](http://antismash.secondarymetabolites.org/help#terpene) | 21883 | - | - |
| Cluster 4 | [T3pks](http://antismash.secondarymetabolites.org/help#t3pks) | 41109 | - | - |
| Cluster 5 | [Transatpks](http://antismash.secondarymetabolites.org/help#transatpks) | 100495 | Difficidin biosynthetic gene cluster (100% of genes show similarity) | 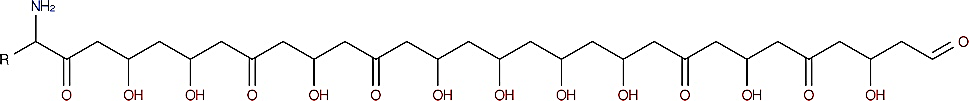 |
| Cluster 6 | Microcin | 20148 | - | - |
| Cluster 7 | [Nrps](http://antismash.secondarymetabolites.org/help#nrps)-[Bacteriocin](http://antismash.secondarymetabolites.org/help#bacteriocin) | 66791 | Bacillibactin biosynthetic gene cluster (100% of genes show similarity) | 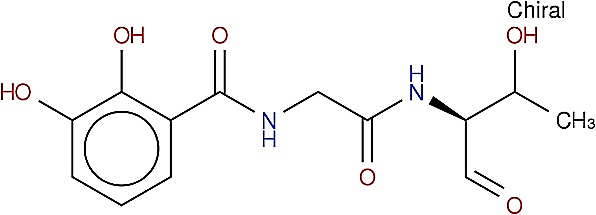 |
| Cluster 8 | [Other](http://antismash.secondarymetabolites.org/help#other) | 41418 | Bacilysin biosynthetic gene cluster (100% of genes show similarity) | - |
| Cluster 9 | Microcin | 20148 | - | - |
| Cluster 10 | Microcin | 20148 | - | - |
| Cluster 11 | Microcin | 25974 | - | - |
| Cluster 12 | Microcin | 26086 | - | - |

Table S1. Continued.

| Strain clusters | Type | Length (bp) | Most similar known clusters | Predicted core clusters |
| --- | --- | --- | --- | --- |
| **CECT 8237** | | | | |
| Cluster 13 | [Nrps](http://antismash.secondarymetabolites.org/help#nrps)-[Transatpks](http://antismash.secondarymetabolites.org/help#transatpks) | 77762 | Locillomycin biosynthetic gene cluster (35% of genes show similarity) | 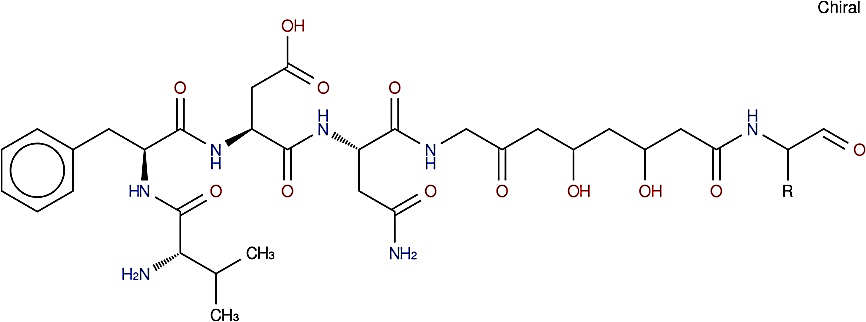 |
| Cluster 14 | [Nrps](http://antismash.secondarymetabolites.org/help#nrps) | 65406 | Surfactin biosynthetic gene cluster (91% of genes show similarity) | 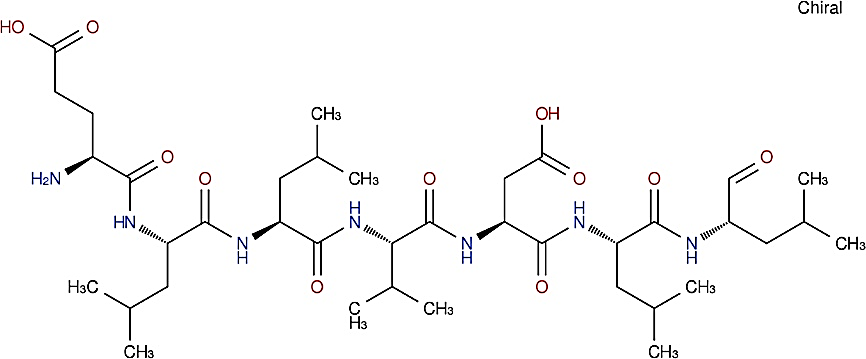 |
| Cluster 15 | [Microcin](http://antismash.secondarymetabolites.org/help#microcin)-[Lantipeptide](http://antismash.secondarymetabolites.org/help#lantipeptide) | 46063 | Haloduracin alpha / haloduracin beta biosynthetic gene cluster (40% of genes show similarity) | - |
| Cluster 16 | [Microcin](http://antismash.secondarymetabolites.org/help#microcin) | 20148 | - | - |
| Cluster 17 | [Otherks](http://antismash.secondarymetabolites.org/help#otherks) | 41244 | - | - |
| Cluster 18 | [Terpene](http://antismash.secondarymetabolites.org/help#terpene) | 20740 | - | - |
| Cluster 19 | [Transatpks](http://antismash.secondarymetabolites.org/help#transatpks) | 85893 | Macrolactin biosynthetic gene cluster (100% of genes show similarity) | 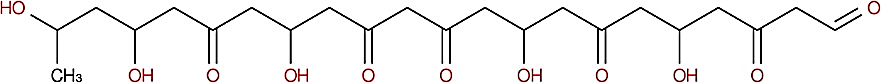 |
| Cluster 20 | Nrps-Transatpks | 79219 | Bacillaene biosynthetic gene cluster (92% of genes show similarity) | 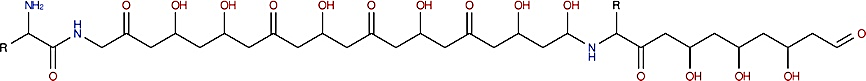 |

Table S1. Continued.

| Strain clusters | Type | Length (bp) | Most similar known clusters | Predicted core clusters |
| --- | --- | --- | --- | --- |
| **CC178** | | | | |
| Cluster 1 | [Microcin](http://antismash.secondarymetabolites.org/help#microcin) | 20148 | - | - |
| Cluster 2 | [Microcin](http://antismash.secondarymetabolites.org/help#microcin) | 20148 | - | - |
| Cluster 3 | [Microcin](http://antismash.secondarymetabolites.org/help#microcin) | 25975 | - | - |
| Cluster 4 | [Microcin](http://antismash.secondarymetabolites.org/help#microcin) | 26086 | - | - |
| Cluster 5 | [Nrps](http://antismash.secondarymetabolites.org/help#nrps) | 65407 | Surfactin biosynthetic gene cluster (91% of genes show similarity) | 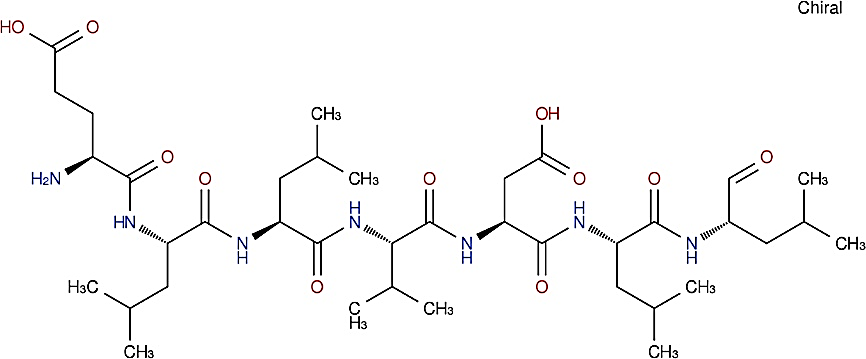 |
| Cluster 6 | [Microcin](http://antismash.secondarymetabolites.org/help#microcin) | 20148 | - | - |
| Cluster 7 | [Microcin](http://antismash.secondarymetabolites.org/help#microcin) | 20148 | - | - |
| Cluster 8 | [Otherks](http://antismash.secondarymetabolites.org/help#otherks) | 41244 | - | - |
| Cluster 9 | [Terpene](http://antismash.secondarymetabolites.org/help#terpene) | 20740 | - | - |
| Cluster 10 | [Transatpks](http://antismash.secondarymetabolites.org/help#transatpks) | 85899 | Macrolactin biosynthetic gene cluster (100% of genes show similarity) | - |
| Cluster 11 | [Nrps](http://antismash.secondarymetabolites.org/help#nrps)-[Transatpks](http://antismash.secondarymetabolites.org/help#transatpks) | 102683 | Bacillaene biosynthetic gene cluster (100% of genes show similarity) | 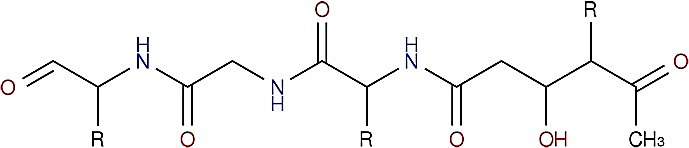 |
| Cluster 12 | [Nrps](http://antismash.secondarymetabolites.org/help#nrps)-[Transatpks](http://antismash.secondarymetabolites.org/help#transatpks) | 137825 | Fengycin biosynthetic gene cluster (100% of genes show similarity) | 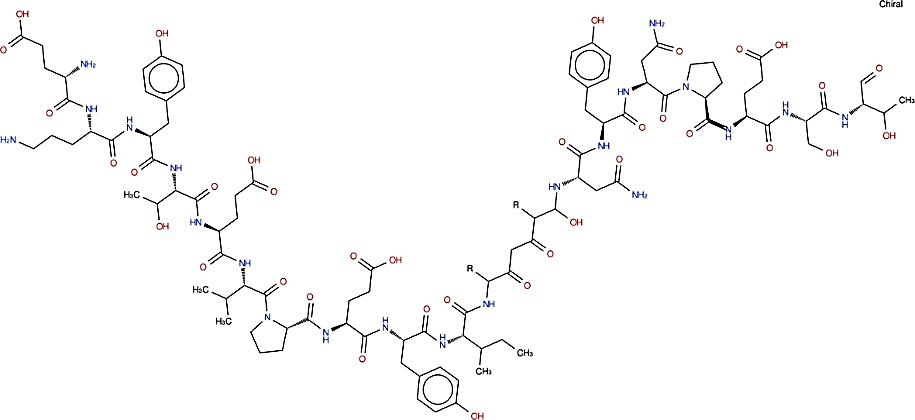 |

Table S1. Continued.

| Strain clusters | Type | Length (bp) | Most similar known clusters | Predicted core clusters |
| --- | --- | --- | --- | --- |
| **CC178** | | | | |
| Cluster 13 | [Terpene](http://antismash.secondarymetabolites.org/help#terpene) | 21883 | - | - |
| Cluster 14 | [T3pks](http://antismash.secondarymetabolites.org/help#t3pks) | 41109 | - | - |
| Cluster 15 | [Transatpks](http://antismash.secondarymetabolites.org/help#transatpks) | 100447 | Difficidin biosynthetic gene cluster (100% of genes show similarity) | 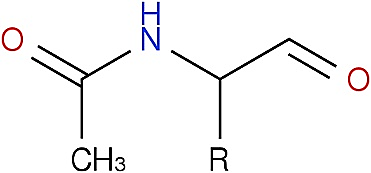 |
| Cluster 16 | [Nrps](http://antismash.secondarymetabolites.org/help#nrps) | 55810 | - | 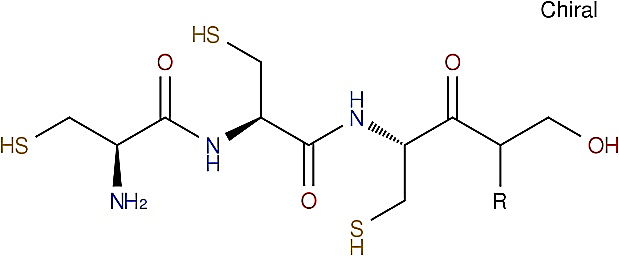 |
| Cluster 17 | [Microcin](http://antismash.secondarymetabolites.org/help#microcin) | 20148 | - | - |
| Cluster 18 | [Nrps](http://antismash.secondarymetabolites.org/help#nrps)-[Bacteriocin](http://antismash.secondarymetabolites.org/help#bacteriocin) | 66791 | Bacillibactin biosynthetic gene cluster (100% of genes show similarity) | 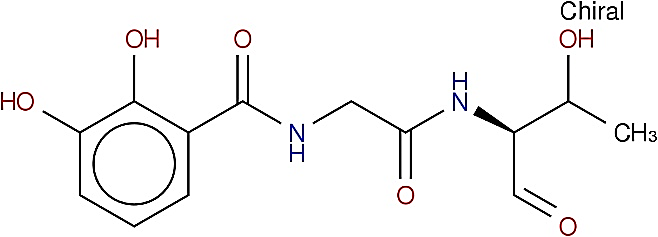 |
| Cluster 19 | Other | 41418 | Bacilysin biosynthetic gene cluster (100% of genes show similarity) | - |

Table S1. Continued.

| Strain clusters | Type | Length (bp) | Most similar known clusters | Predicted core clusters |
| --- | --- | --- | --- | --- |
| **UASWS BA1** | | | | |
| Cluster 1 | [Microcin](http://antismash.secondarymetabolites.org/help#microcin) | 10967 | - | - |
| Cluster 2 | [Other](http://antismash.secondarymetabolites.org/help#other) | 41418 | Bacilysin biosynthetic gene cluster (100% of genes show similarity) | - |
| Cluster 3 | [Bacteriocin](http://antismash.secondarymetabolites.org/help#bacteriocin)-[Nrps](http://antismash.secondarymetabolites.org/help#nrps) | 51793 | Bacillibactin biosynthetic gene cluster (92% of genes show similarity) | 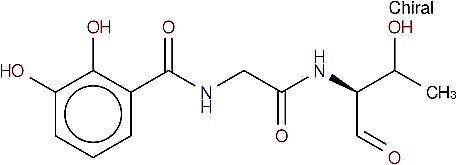 |
| Cluster 4 | Microcin | 10967 | - | - |
| Cluster 5 | Nrps-Transatpks | 87977 | Fengycin biosynthetic gene cluster (80% of genes show similarity) | 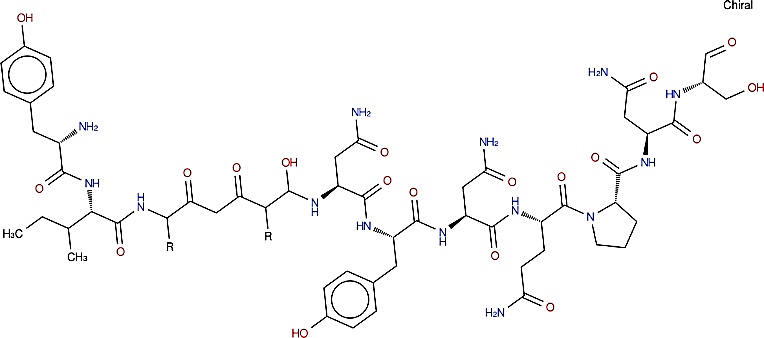 |
| Cluster 6 | Transatpks-Nrps | 103001 | Bacillaene biosynthetic gene cluster (92% of genes show similarity) | 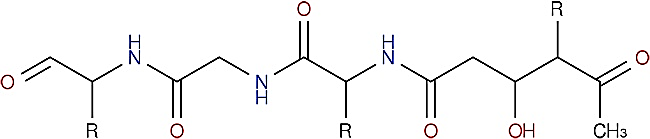 |
| Cluster 7 | Transatpks | 85881 | Macrolactin biosynthetic gene cluster (90% of genes show similarity) | - |
| Cluster 8 | Lantipeptide | 28889 | - | - |
| Cluster 9 | Nrps | 38881 | Plipastatin biosynthetic gene cluster (38% of genes show similarity) | 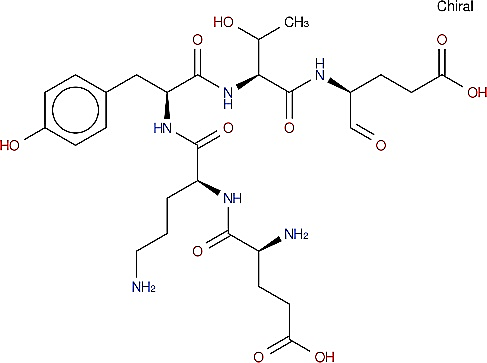 |

Table S1. Continued.

| Strain clusters | Type | Length (bp) | Most similar known clusters | Predicted core clusters |
| --- | --- | --- | --- | --- |
| **UASWS BA1** | | | | |
| Cluster 10 | Terpene | 21883 | - | - |
| Cluster 11 | [T3pks](http://antismash.secondarymetabolites.org/help#t3pks) | 41109 | - | - |
| Cluster 12 | Transatpks | 46304 | Difficidin biosynthetic gene cluster (53% of genes show similarity) | 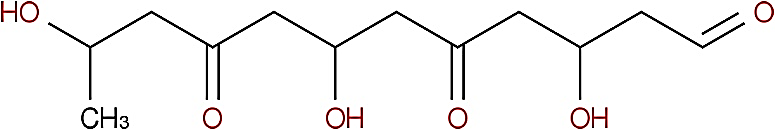 |
| Cluster 13 | Terpene | 20740 | - | - |
| Cluster 14 | Otherks | 41244 | Butirosin biosynthetic gene cluster (7% of genes show similarity) | - |
| Cluster 15 | Transatpks | 29304 | Difficidin biosynthetic gene cluster (40% of genes show similarity) | 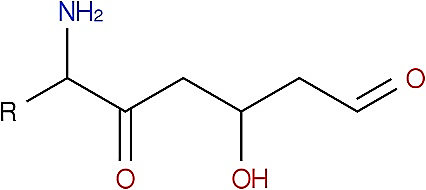 |
| Cluster 16 | Nrps | 27023 | Surfactin biosynthetic gene cluster (39% of genes show similarity) | 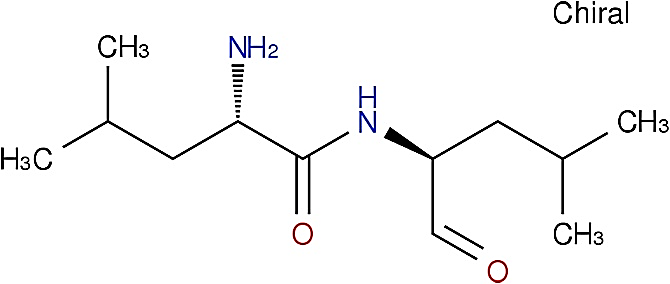 |
| Cluster 17 | [Microcin](http://antismash.secondarymetabolites.org/help#microcin) | 10967 | - | - |
| Cluster 18 | Nrps | 29481 | Surfactin biosynthetic gene cluster (47% of genes show similarity) | 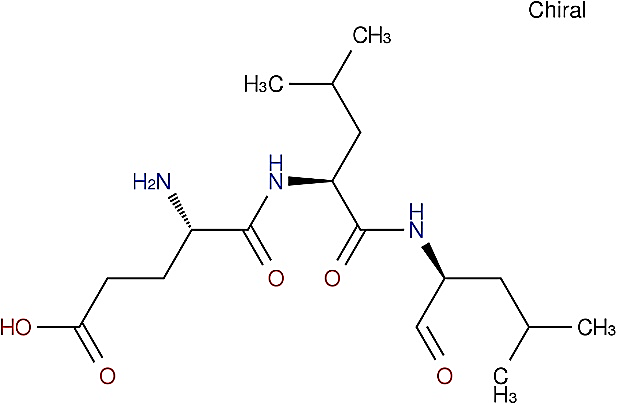 |
| Cluster 19 | Microcin | 10967 | - | - |
| Cluster 20 | Microcin | 10967 | - | - |

Table S1. Continued.

| Strain clusters | Type | Length (bp) | Most similar known clusters | Predicted core clusters |
| --- | --- | --- | --- | --- |
| **UASWS BA1** | | | | |
| Cluster 21 | [Transatpks](http://antismash.secondarymetabolites.org/help#transatpks) | 24511 | Difficidin biosynthetic gene cluster (26% of genes show similarity) | 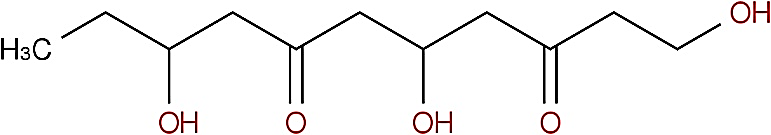 |
| Cluster 22 | [Microcin](http://antismash.secondarymetabolites.org/help#microcin) | 18574 | - | - |
| Cluster 23 | [Nrps](http://antismash.secondarymetabolites.org/help#nrps) | 12631 | Surfactin biosynthetic gene cluster (8% of genes show similarity) | 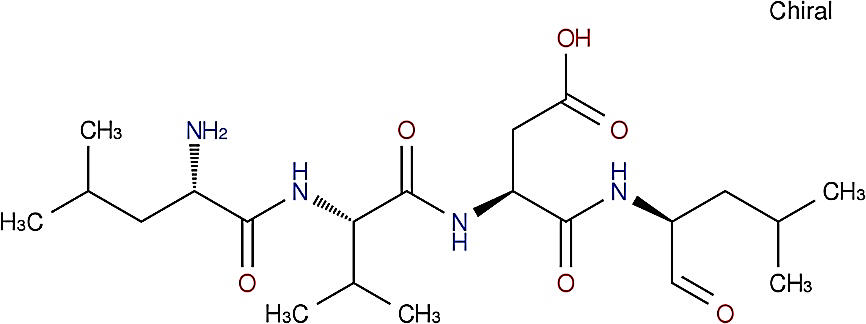 |
| Cluster 24 | [Nrps](http://antismash.secondarymetabolites.org/help#nrps) | 10547 | Fengycin biosynthetic gene cluster (20% of genes show similarity) | 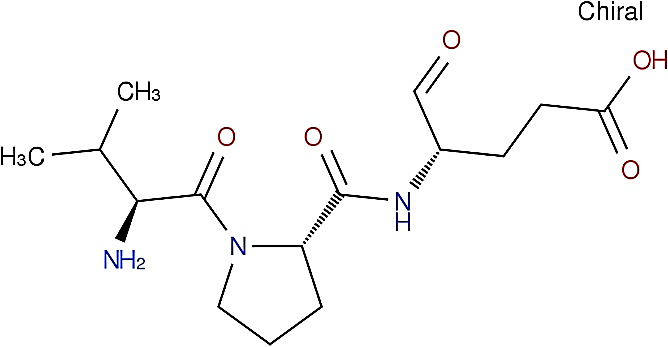 |
| Cluster 25 | [Microcin](http://antismash.secondarymetabolites.org/help#microcin) | 4715 | - | - |
| Cluster 26 | [Microcin](http://antismash.secondarymetabolites.org/help#microcin) | 2740 | - | - |

Table S1. Continued.

| Strain clusters | Type | Length (bp) | Most similar known clusters | Predicted core clusters |
| --- | --- | --- | --- | --- |
| **B1895** | | | | |
| Cluster 1 | [Transatpks](http://antismash.secondarymetabolites.org/help#transatpks) | 80429 | Macrolactin biosynthetic gene cluster (100% of genes show similarity) | 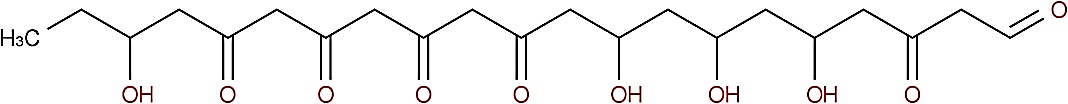 |
| Cluster 2 | [Microcin](http://antismash.secondarymetabolites.org/help#microcin) | 20148 | - | - |
| Cluster 3 | [T3pks](http://antismash.secondarymetabolites.org/help#t3pks) | 41109 | - | - |
| Cluster 4 | [Other](http://antismash.secondarymetabolites.org/help#other) | 41418 | Bacilysin biosynthetic gene cluster (100% of genes show similarity) | - |
| Cluster 5 | [Transatpks](http://antismash.secondarymetabolites.org/help#transatpks) | 100443 | Difficidin biosynthetic gene cluster (100% of genes show similarity) | 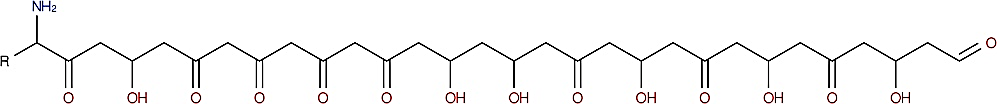 |
| Cluster 6 | [Transatpks](http://antismash.secondarymetabolites.org/help#transatpks) | 91353 | Bacillaene biosynthetic gene cluster (100% of genes show similarity) | 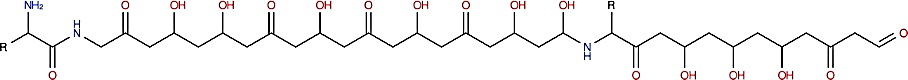 |
| Cluster 7 | [Nrps](http://antismash.secondarymetabolites.org/help#nrps)-[Bacteriocin](http://antismash.secondarymetabolites.org/help#bacteriocin) | 50393 | Bacillibactin biosynthetic gene cluster (100% of genes show similarity) | 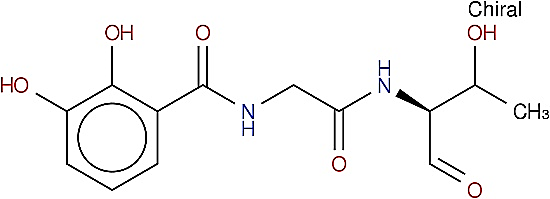 |
| Cluster 8 | [Nrps](http://antismash.secondarymetabolites.org/help#nrps) | 26012 | Surfactin biosynthetic gene cluster (39% of genes show similarity) | - |
| Cluster 9 | [Terpene](http://antismash.secondarymetabolites.org/help#terpene) | 20740 | - | - |
| Cluster 10 | [Nrps](http://antismash.secondarymetabolites.org/help#nrps) | 38532 | Surfactin biosynthetic gene cluster (52% of genes show similarity) | 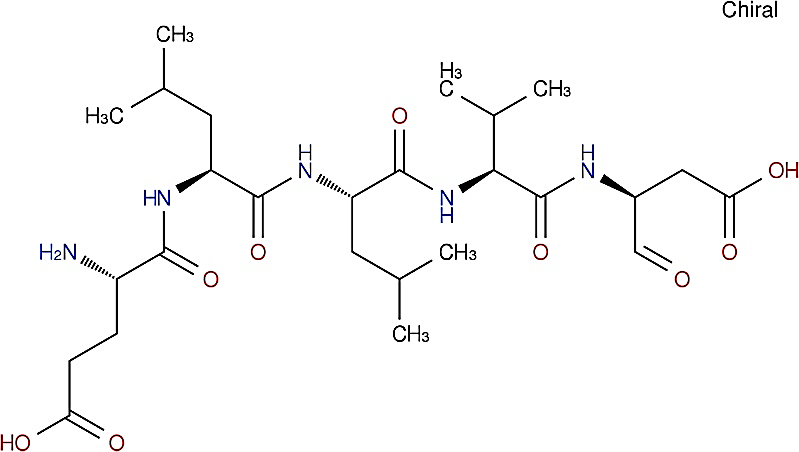 |
| Cluster 11 | [Terpene](http://antismash.secondarymetabolites.org/help#terpene) | 21883 | - | - |

Table S1. Continued.

| Strain clusters | Type | Length (bp) | Most similar known clusters | Predicted core clusters |
| --- | --- | --- | --- | --- |
| **B1895** | | | | |
| Cluster 12 | [Nrps](http://antismash.secondarymetabolites.org/help#nrps)-[Transatpks](http://antismash.secondarymetabolites.org/help#transatpks) | 87544 | Fengycin biosynthetic gene cluster (86% of genes show similarity) | 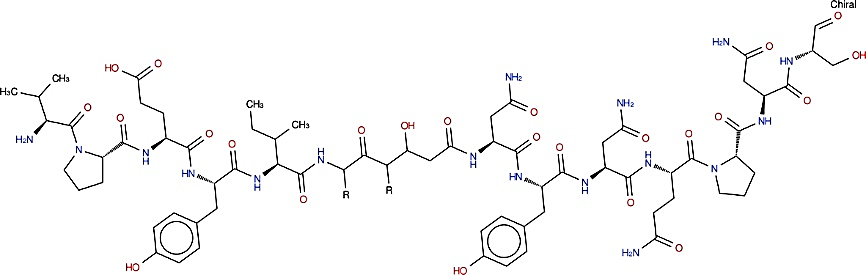 |
| Cluster 13 | [Otherks](http://antismash.secondarymetabolites.org/help#otherks) | 41244 | - | - |
| Cluster 14 | Microcin | 17643 | - | - |
| Cluster 15 | [Microcin](http://antismash.secondarymetabolites.org/help#microcin) | 12413 | - | - |
| Cluster 16 | [Microcin](http://antismash.secondarymetabolites.org/help#microcin) | 20148 | - | - |
| Cluster 17 | [Microcin](http://antismash.secondarymetabolites.org/help#microcin) | 11266 | - | - |
| Cluster 18 | [Nrps](http://antismash.secondarymetabolites.org/help#nrps) | 21636 | Plipastatin biosynthetic gene cluster (53% of genes show similarity) | 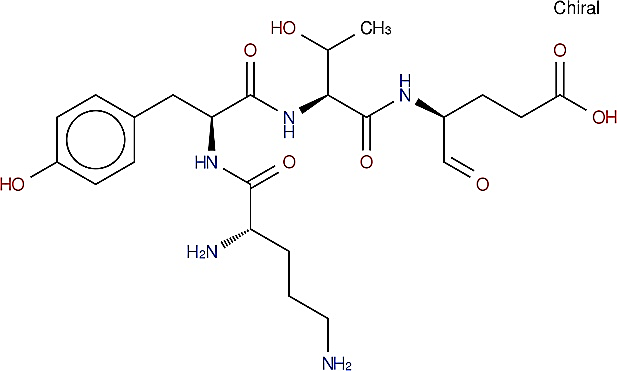 |
| Cluster 19 | [Bacteriocin](http://antismash.secondarymetabolites.org/help#bacteriocin) | 5939 | - | - |
| Cluster 20 | [Ladderane](http://antismash.secondarymetabolites.org/help#ladderane) | 3005 | - | - |
| Cluster 21 | [Lantipeptide](http://antismash.secondarymetabolites.org/help#lantipeptide) | 2899 | - | - |
| Cluster 22 | [Microcin](http://antismash.secondarymetabolites.org/help#microcin) | 2042 | - | - |
| Cluster 23 | [Microcin](http://antismash.secondarymetabolites.org/help#microcin) | 2037 | - | - |
| Cluster 24 | [Lantipeptide](http://antismash.secondarymetabolites.org/help#lantipeptide) | 1812 | - | - |
| Cluster 25 | [Microcin](http://antismash.secondarymetabolites.org/help#microcin) | 1673 | - | - |
| Cluster 26 | [Microcin](http://antismash.secondarymetabolites.org/help#microcin) | 1641 | - | - |

Table S1. Continued.

| Strain clusters | Type | Length (bp) | Most similar known clusters | Predicted core clusters |
| --- | --- | --- | --- | --- |
| **XK-4-1** | | | | |
| Cluster 1 | [Microcin](http://antismash.secondarymetabolites.org/help#microcin) | 2225 | - | - |
| Cluster 2 | [Microcin](http://antismash.secondarymetabolites.org/help#microcin) | 1282 | - | - |
| Cluster 3 | [Other](http://antismash.secondarymetabolites.org/help#other) | 41418 | Bacilysin biosynthetic gene cluster (85% of genes show similarity) | - |
| Cluster 4 | [T3pks](http://antismash.secondarymetabolites.org/help#t3pks) | 41109 | - | - |
| Cluster 5 | [Nrps](http://antismash.secondarymetabolites.org/help#nrps)-[Transatpks](http://antismash.secondarymetabolites.org/help#transatpks) | 93304 | Bacillaene biosynthetic gene cluster (100% of genes show similarity) | 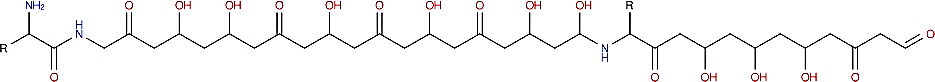 |
| Cluster 6 | [Bacteriocin](http://antismash.secondarymetabolites.org/help#bacteriocin)-[Nrps](http://antismash.secondarymetabolites.org/help#nrps)-[Transatpks](http://antismash.secondarymetabolites.org/help#transatpks) | 136302 | Fengycin biosynthetic gene cluster (100% of genes show similarity) | 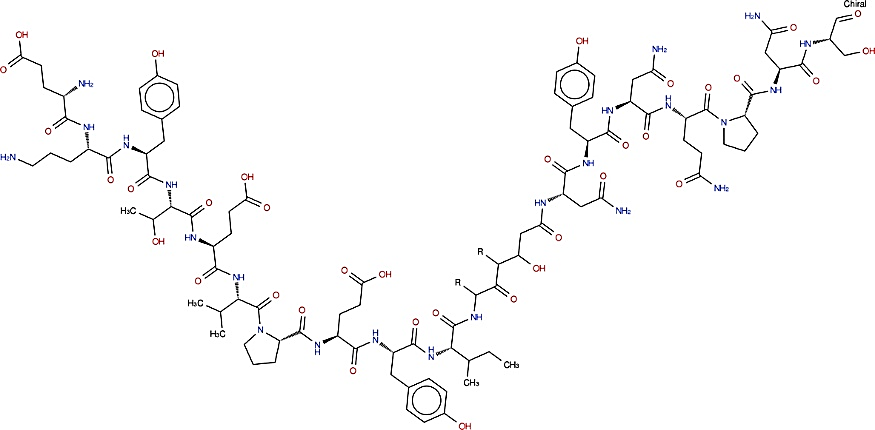 |
| Cluster 7 | [Transatpks](http://antismash.secondarymetabolites.org/help#transatpks) | 46457 | Difficidin biosynthetic gene cluster (53% of genes show similarity) | 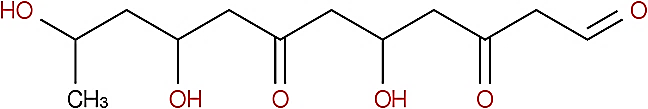 |
| Cluster 8 | [Lantipeptide](http://antismash.secondarymetabolites.org/help#lantipeptide) | 27143 | Haloduracin alpha / haloduracin beta biosynthetic gene cluster (40% of genes show similarity) | - |
| Cluster 9 | [Ladderane](http://antismash.secondarymetabolites.org/help#ladderane) | 41217 | - | - |
| Cluster 10 | [Transatpks](http://antismash.secondarymetabolites.org/help#transatpks) | 28654 | Difficidin biosynthetic gene cluster (46% of genes show similarity) | 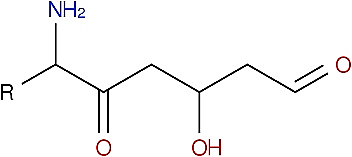 |
| Cluster 11 | [Transatpks](http://antismash.secondarymetabolites.org/help#transatpks) | 24138 | Difficidin biosynthetic gene cluster (26% of genes show similarity) | 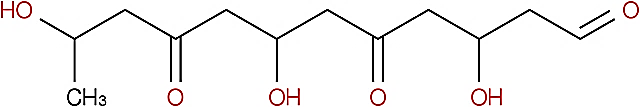 |

Table S1. Continued.

| Strain clusters | Type | Length (bp) | Most similar known clusters | Predicted core clusters |
| --- | --- | --- | --- | --- |
| **XK-4-1** | | | | |
| Cluster 12 | [Otherks](http://antismash.secondarymetabolites.org/help#otherks) | 41244 | - | - |
| Cluster 13 | [Terpene](http://antismash.secondarymetabolites.org/help#terpene) | 20740 | - | - |
| Cluster 14 | [Nrps](http://antismash.secondarymetabolites.org/help#nrps) | 9538 | Surfactin biosynthetic gene cluster (8% of genes show similarity) | 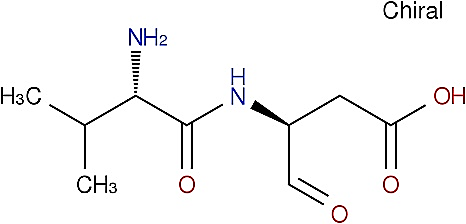 |
| Cluster 15 | [Nrps](http://antismash.secondarymetabolites.org/help#nrps) | 25727 | Surfactin biosynthetic gene cluster (47% of genes show similarity) | - |
| Cluster 16 | [Terpene](http://antismash.secondarymetabolites.org/help#terpene) | 21883 | - | - |
| Cluster 17 | [Nrps](http://antismash.secondarymetabolites.org/help#nrps) | 27684 | Surfactin biosynthetic gene cluster (47% of genes show similarity) | 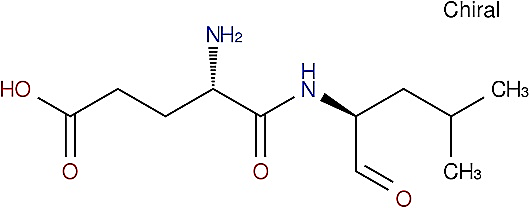 |
| Cluster 18 | [Transatpks](http://antismash.secondarymetabolites.org/help#transatpks) | 85884 | Macrolactin biosynthetic gene cluster (100% of genes show similarity) | 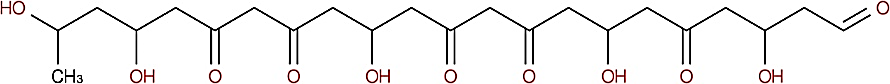 |
| Cluster 19 | [Nrps](http://antismash.secondarymetabolites.org/help#nrps)-[Bacteriocin](http://antismash.secondarymetabolites.org/help#bacteriocin) | 66792 | Bacillibactin biosynthetic gene cluster (100% of genes show similarity) |  |

Table S1. Continued.

| Strain clusters | Type | Length (bp) | Most similar known clusters | Predicted core clusters |
| --- | --- | --- | --- | --- |
| **LL3** | | | | |
| Cluster 1 | [Microcin](http://antismash.secondarymetabolites.org/help#microcin) | 20148 | - | - |
| Cluster 2 | [Microcin](http://antismash.secondarymetabolites.org/help#microcin) | 20148 | - | - |
| Cluster 3 | [Microcin](http://antismash.secondarymetabolites.org/help#microcin) | 20148 | - | - |
| Cluster 4 | [Microcin](http://antismash.secondarymetabolites.org/help#microcin) | 20148 | - | - |
| Cluster 5 | [Nrps](http://antismash.secondarymetabolites.org/help#nrps) | 65410 | Surfactin biosynthetic gene cluster (82% of genes show similarity) |  |
| Cluster 6 | [Microcin](http://antismash.secondarymetabolites.org/help#microcin) | 20148 | - | - |
| Cluster 7 | [Lantipeptide](http://antismash.secondarymetabolites.org/help#lantipeptide) | 22597 | - | - |
| Cluster 8 | [Microcin](http://antismash.secondarymetabolites.org/help#microcin) | 20148 | - | - |
| Cluster 9 | [Otherks](http://antismash.secondarymetabolites.org/help#otherks) | 41244 | - | - |
| Cluster 10 | [Terpene](http://antismash.secondarymetabolites.org/help#terpene) | 20743 | - | - |
| Cluster 11 | [Nrps](http://antismash.secondarymetabolites.org/help#nrps)-[Transatpks](http://antismash.secondarymetabolites.org/help#transatpks) | 102704 | Bacillaene biosynthetic gene cluster (100% of genes show similarity) |  |
| Cluster 12 | [Nrps](http://antismash.secondarymetabolites.org/help#nrps)-[Transatpks](http://antismash.secondarymetabolites.org/help#transatpks) | 110420 | Fengycin biosynthetic gene cluster (93% of genes show similarity) |  |
| Cluster 13 | Terpene | 21883 | - | - |

Table S1. Continued.

| Strain clusters | Type | Length (bp) | Most similar known clusters | Predicted core clusters |
| --- | --- | --- | --- | --- |
| **LL3** | | | | |
| Cluster 14 | T3pks | 41109 | - | - |
| Cluster 15 | [Microcin](http://antismash.secondarymetabolites.org/help#microcin) | 20148 | - | - |
| Cluster 16 | [Nrps](http://antismash.secondarymetabolites.org/help#nrps)-[Bacteriocin](http://antismash.secondarymetabolites.org/help#bacteriocin) | 66775 | Bacillibactin biosynthetic gene cluster (100% of genes show similarity) |  |
| Cluster 17 | Other | 41454 | Bacilysin biosynthetic gene cluster (100% of genes show similarity) | - |

Table S1. Continued.

| Strain clusters | Type | Length (bp) | Most similar known clusters | Predicted core clusters |
| --- | --- | --- | --- | --- |
| **TF28** | | | | |
| Cluster 1 | [Transatpks](http://antismash.secondarymetabolites.org/help#transatpks) | 20721 | Difficidin biosynthetic gene cluster (46% of genes show similarity) |  |
| Cluster 2 | [Transatpks](http://antismash.secondarymetabolites.org/help#transatpks) | 23107 | Difficidin biosynthetic gene cluster (26% of genes show similarity) |  |
| Cluster 3 | [Transatpks](http://antismash.secondarymetabolites.org/help#transatpks) | 41921 | Difficidin biosynthetic gene cluster (46% of genes show similarity) |  |
| Cluster 4 | [T3pks](http://antismash.secondarymetabolites.org/help#t3pks) | 41109 | - | - |
| Cluster 5 | [Terpene](http://antismash.secondarymetabolites.org/help#terpene) | 29898 | - | - |
| Cluster 6 | [Nrps](http://antismash.secondarymetabolites.org/help#nrps) | 2038 | - | - |
| Cluster 7 | [Nrps](http://antismash.secondarymetabolites.org/help#nrps) | 13004 | Fengycin biosynthetic gene cluster (20% of genes show similarity) |  |
| Cluster 8 | [Nrps](http://antismash.secondarymetabolites.org/help#nrps) | 9538 | Fengycin biosynthetic gene cluster (13% of genes show similarity) |  |

Table S1. Continued.

| Strain clusters | Type | Length (bp) | Most similar known clusters | Predicted core clusters |
| --- | --- | --- | --- | --- |
| **TF28** | | | | |
| Cluster 9 | [Nrps](http://antismash.secondarymetabolites.org/help#nrps) | 9903 | Plipastatin biosynthetic gene cluster (38% of genes show similarity) |  |
| Cluster 10 | Nrps-Transatpks | 36683 | Mycosubtilin biosynthetic gene cluster (80% of genes show similarity) |  |
| Cluster 11 | [Nrps](http://antismash.secondarymetabolites.org/help#nrps) | 4523 | - | - |
| Cluster 12 | Nrps-Bacteriocin | 48352 | Bacillomycin biosynthetic gene cluster (30% of genes show similarity) |  |
| Cluster 13 | [Transatpks](http://antismash.secondarymetabolites.org/help#transatpks)-[Nrps](http://antismash.secondarymetabolites.org/help#nrps) | 31539 | Bacillaene biosynthetic gene cluster (28% of genes show similarity) |  |
| Cluster 14 | [Transatpks](http://antismash.secondarymetabolites.org/help#transatpks) | 21489 | Bacillaene biosynthetic gene cluster (21% of genes show similarity) |  |

Table S1. Continued.

| Strain clusters | Type | Length (bp) | Most similar known clusters | Predicted core clusters |
| --- | --- | --- | --- | --- |
| **TF28** | | | | |
| Cluster 15 | [Nrps](http://antismash.secondarymetabolites.org/help#nrps)-[Transatpks](http://antismash.secondarymetabolites.org/help#transatpks) | 29357 | Bacillaene biosynthetic gene cluster (64% of genes show similarity) |  |
| Cluster 16 | [Transatpks](http://antismash.secondarymetabolites.org/help#transatpks) | 41370 | Macrolactin biosynthetic gene cluster (50% of genes show similarity) |  |
| Cluster 17 | [Terpene](http://antismash.secondarymetabolites.org/help#terpene) | 17417 | - | - |
| Cluster 18 | Otherks | 41244 | Butirosin biosynthetic gene cluster (7% of genes show similarity) | - |
| Cluster 19 | Nrps | 43742 | Surfactin biosynthetic gene cluster (47% of genes show similarity) | - |
| Cluster 20 | [Nrps](http://antismash.secondarymetabolites.org/help#nrps) | 9492 | Surfactin biosynthetic gene cluster (8% of genes show similarity) |  |
| Cluster 21 | [Nrps](http://antismash.secondarymetabolites.org/help#nrps) | 27586 | Surfactin biosynthetic gene cluster (43% of genes show similarity) |  |
| Cluster 22 | [Nrps](http://antismash.secondarymetabolites.org/help#nrps)-[Transatpks](http://antismash.secondarymetabolites.org/help#transatpks) | 46690 | - | - |
| Cluster 23 | [Nrps](http://antismash.secondarymetabolites.org/help#nrps) | 6583 | - | - |
| Cluster 24 | [Other](http://antismash.secondarymetabolites.org/help#other) | 24721 | Bacilysin biosynthetic gene cluster (85% of genes show similarity) | - |

Table S1. Continued.

| Strain clusters | Type | Length (bp) | Most similar known clusters | Predicted core clusters |
| --- | --- | --- | --- | --- |
| **TF28** | | | | |
| Cluster 25 | [Nrps](http://antismash.secondarymetabolites.org/help#nrps) | 23249 | - | - |
| Cluster 26 | [Nrps](http://antismash.secondarymetabolites.org/help#nrps) | 6732 | - | - |
| Cluster 27 | Nrps | 6397 | - | - |
| Cluster 28 | [Bacteriocin](http://antismash.secondarymetabolites.org/help#bacteriocin)-[Nrps](http://antismash.secondarymetabolites.org/help#nrps) | 49438 | Bacillibactin biosynthetic gene cluster (92% of genes show similarity) |  |

Table S1. Continued.

| Strain clusters | Type | Length (bp) | Most similar known clusters | Predicted core clusters |
| --- | --- | --- | --- | --- |
| **RHNK22** | | | | |
| Cluster 1 | [Transatpks](http://antismash.secondarymetabolites.org/help#transatpks) | 85875 | Macrolactin biosynthetic gene cluster (100% of genes show similarity) |  |
| Cluster 2 | [Nrps](http://antismash.secondarymetabolites.org/help#nrps) | 28133 | Surfactin biosynthetic gene cluster (43% of genes show similarity) |  |
| Cluster 3 | [Nrps](http://antismash.secondarymetabolites.org/help#nrps)-[Transatpks](http://antismash.secondarymetabolites.org/help#transatpks) | 46658 | - |  |
| Cluster 4 | [Microcin](http://antismash.secondarymetabolites.org/help#microcin) | 1599 | - | - |
| Cluster 5 | Terpene | 20740 | - | - |
| Cluster 6 | [Otherks](http://antismash.secondarymetabolites.org/help#otherks) | 41244 | Butirosin biosynthetic gene cluster (7% of genes show similarity) | - |
| Cluster 7 | [Lantipeptide](http://antismash.secondarymetabolites.org/help#lantipeptide)-[Nrps](http://antismash.secondarymetabolites.org/help#nrps) | 28858 | Locillomycin biosynthetic gene cluster (35% of genes show similarity) | - |
| Cluster 8 | [Nrps](http://antismash.secondarymetabolites.org/help#nrps) | 9560 | Surfactin biosynthetic gene cluster (8% of genes show similarity) |  |
| Cluster 9 | [Nrps](http://antismash.secondarymetabolites.org/help#nrps)-[Bacteriocin](http://antismash.secondarymetabolites.org/help#bacteriocin) | 66792 | Bacillibactin biosynthetic gene cluster (100% of genes show similarity) |  |

Table S1. Continued.

| Strain clusters | Type | Length (bp) | Most similar known clusters | Predicted core clusters |
| --- | --- | --- | --- | --- |
| **RHNK22** | | | | |
| Cluster 10 | [Lantipeptide](http://antismash.secondarymetabolites.org/help#lantipeptide) | 26661 | - | - |
| Cluster 11 | [Other](http://antismash.secondarymetabolites.org/help#other) | 41418 | Bacilysin biosynthetic gene cluster (100% of genes show similarity) | - |
| Cluster 12 | [Nrps](http://antismash.secondarymetabolites.org/help#nrps) | 10078 | Fengycin biosynthetic gene cluster (13% of genes show similarity) |  |
| Cluster 13 | [Nrps](http://antismash.secondarymetabolites.org/help#nrps) | 13441 | Fengycin biosynthetic gene cluster (20% of genes show similarity) |  |
| Cluster 14 | [Other](http://antismash.secondarymetabolites.org/help#other) | 1357 | - | - |
| Cluster 15 | [T3pks](http://antismash.secondarymetabolites.org/help#t3pks) | 41109 | - | - |
| Cluster 16 | [Nrps](http://antismash.secondarymetabolites.org/help#nrps) | 6935 | - |  |

Table S1. Continued.

| Strain clusters | Type | Length (bp) | Most similar known clusters | Predicted core clusters |
| --- | --- | --- | --- | --- |
| **RHNK22** | | | | |
| Cluster 17 | [Transatpks](http://antismash.secondarymetabolites.org/help#transatpks) | 100301 | Difficidin biosynthetic gene cluster (93% of genes show similarity) |  |
| Cluster 18 | [Nrps](http://antismash.secondarymetabolites.org/help#nrps) | 25300 | - | - |
| Cluster 19 | [Nrps](http://antismash.secondarymetabolites.org/help#nrps)-[Transatpks](http://antismash.secondarymetabolites.org/help#transatpks) | 102830 | Bacillaene biosynthetic gene cluster (100% of genes show similarity) |  |
| Cluster 20 | [Nrps](http://antismash.secondarymetabolites.org/help#nrps)-[Transatpks](http://antismash.secondarymetabolites.org/help#transatpks) | 113191 | Fengycin biosynthetic gene cluster (100% of genes show similarity) |  |
| Cluster 21 | [Terpene](http://antismash.secondarymetabolites.org/help#terpene) | 21883 | - | - |

Table S1. Continued.

| Strain clusters | Type | Length (bp) | Most similar known clusters | Predicted core clusters |
| --- | --- | --- | --- | --- |
| **Bs006** | | | | |
| Cluster 1 | [Nrps](http://antismash.secondarymetabolites.org/help#nrps) | 7710 | - |  |
| Cluster 2 | [Transatpks](http://antismash.secondarymetabolites.org/help#transatpks) | 53060 | Difficidin biosynthetic gene cluster (60% of genes show similarity) |  |
| Cluster 3 | [T3pks](http://antismash.secondarymetabolites.org/help#t3pks) | 26587 | - | - |
| Cluster 4 | [Transatpks](http://antismash.secondarymetabolites.org/help#transatpks) | 45796 | Difficidin biosynthetic gene cluster (53% of genes show similarity) |  |
| Cluster 5 | [Terpene](http://antismash.secondarymetabolites.org/help#terpene) | 29900 | - | - |
| Cluster 6 | [Nrps](http://antismash.secondarymetabolites.org/help#nrps) | 21611 | Plipastatin biosynthetic gene cluster (30% of genes show similarity) | - |
| Cluster 7 | [Nrps](http://antismash.secondarymetabolites.org/help#nrps) | 13003 | Fengycin biosynthetic gene cluster (20% of genes show similarity) |  |

Table S1. Continued.

| Strain clusters | Type | Length (bp) | Most similar known clusters | Predicted core clusters |
| --- | --- | --- | --- | --- |
| **Bs006** | | | | |
| Cluster 8 | [Nrps](http://antismash.secondarymetabolites.org/help#nrps) | 9537 | Fengycin biosynthetic gene cluster (13% of genes show similarity) |  |
| Cluster 9 | [Nrps](http://antismash.secondarymetabolites.org/help#nrps)-[Transatpks](http://antismash.secondarymetabolites.org/help#transatpks) | 87618 | Fengycin biosynthetic gene cluster (80% of genes show similarity) |  |
| Cluster 10 | [Transatpks](http://antismash.secondarymetabolites.org/help#transatpks) | 85892 | Macrolactin biosynthetic gene cluster (100% of genes show similarity) |  |
| Cluster 11 | [Nrps](http://antismash.secondarymetabolites.org/help#nrps)-[Transatpks](http://antismash.secondarymetabolites.org/help#transatpks) | 102671 | Bacillaene biosynthetic gene cluster (100% of genes show similarity) |  |
| Cluster 12 | [Otherks](http://antismash.secondarymetabolites.org/help#otherks) | 41244 | - | - |
| Cluster 13 | [Terpene](http://antismash.secondarymetabolites.org/help#terpene) | 20740 | - | - |
| Cluster 14 | Nrps | 25779 | Surfactin biosynthetic gene cluster (39% of genes show similarity) | - |
| Cluster 15 | [Nrps](http://antismash.secondarymetabolites.org/help#nrps) | 9722 | Surfactin biosynthetic gene cluster (8% of genes show similarity) |  |

Table S1. Continued.

| Strain clusters | Type | Length (bp) | Most similar known clusters | Predicted core clusters |
| --- | --- | --- | --- | --- |
| **Bs006** | | | | |
| Cluster 16 | [Nrps](http://antismash.secondarymetabolites.org/help#nrps) | 27816 | Surfactin biosynthetic gene cluster (43% of genes show similarity) |  |
| Cluster 17 | [Nrps](http://antismash.secondarymetabolites.org/help#nrps)-[Transatpks](http://antismash.secondarymetabolites.org/help#transatpks) | 46695 | - |  |
| Cluster 18 | [Other](http://antismash.secondarymetabolites.org/help#other) | 41418 | Bacilysin biosynthetic gene cluster (85% of genes show similarity) | - |
| Cluster 19 | [Nrps](http://antismash.secondarymetabolites.org/help#nrps) | 31688 | - |  |
| Cluster 20 | [Nrps](http://antismash.secondarymetabolites.org/help#nrps) | 6397 | - |  |
| Cluster 21 | [Nrps](http://antismash.secondarymetabolites.org/help#nrps) | 2525 | - | - |
| Cluster 22 | [Bacteriocin](http://antismash.secondarymetabolites.org/help#bacteriocin)-[Nrps](http://antismash.secondarymetabolites.org/help#nrps) | 51789 | Bacillibactin biosynthetic gene cluster (92% of genes show similarity) |  |

Table S1. Continued.

| Strain clusters | Type | Length (bp) | Most similar known clusters | Predicted core clusters |
| --- | --- | --- | --- | --- |
| **Bs006** | | | | |
| Cluster 23 | [Lantipeptide](http://antismash.secondarymetabolites.org/help#lantipeptide) | 6098 | Ericin A biosynthetic gene cluster (50% of genes show similarity) | - |
| Cluster 24 | [Lantipeptide](http://antismash.secondarymetabolites.org/help#lantipeptide) | 5391 | Ericin A biosynthetic gene cluster (31% of genes show similarity) | - |

Table S1. Continued.

| Strain clusters | Type | Length (bp) | Most similar known clusters | Predicted core clusters |
| --- | --- | --- | --- | --- |
| **TA208** | | | | |
| Cluster 1 | [Microcin](http://antismash.secondarymetabolites.org/help#microcin) | 37079 | - | - |
| Cluster 2 | [Microcin](http://antismash.secondarymetabolites.org/help#microcin) | 20148 | - | - |
| Cluster 3 | [Microcin](http://antismash.secondarymetabolites.org/help#microcin) | 20148 | - | - |
| Cluster 4 | Nrps | 65410 | - |  |
| Cluster 5 | [Microcin](http://antismash.secondarymetabolites.org/help#microcin) | 20148 | - | - |
| Cluster 6 | [Lantipeptide](http://antismash.secondarymetabolites.org/help#lantipeptide) | 22597 | - | - |
| Cluster 7 | [Microcin](http://antismash.secondarymetabolites.org/help#microcin) | 20148 | - | - |
| Cluster 8 | [Otherks](http://antismash.secondarymetabolites.org/help#otherks) | 41244 | - | - |
| Cluster 9 | [Terpene](http://antismash.secondarymetabolites.org/help#terpene) | 20743 | - | - |
| Cluster 10 | [T3pks](http://antismash.secondarymetabolites.org/help#t3pks) | 41109 | - | - |
| Cluster 11 | [Terpene](http://antismash.secondarymetabolites.org/help#terpene) | 21883 | - | - |
| Cluster 12 | [Nrps](http://antismash.secondarymetabolites.org/help#nrps)-[Transatpks](http://antismash.secondarymetabolites.org/help#transatpks) | 110421 | Fengycin biosynthetic gene cluster (93% of genes show similarity) |  |
| Cluster 13 | Transatpks-Nrps | 102704 | Bacillaene biosynthetic gene cluster (100% of genes show similarity) |  |

Table S1. Continued.

| Strain clusters | Type | Length (bp) | Most similar known clusters | Predicted core clusters |
| --- | --- | --- | --- | --- |
| **TA208** | | | | |
| Cluster 14 | [Microcin](http://antismash.secondarymetabolites.org/help#microcin) | 20148 | - | - |
| Cluster 15 | [Nrps](http://antismash.secondarymetabolites.org/help#nrps)-[Bacteriocin](http://antismash.secondarymetabolites.org/help#bacteriocin) | 66773 | Bacillibactin biosynthetic gene cluster (100% of genes show similarity) |  |
| Cluster 16 | [Other](http://antismash.secondarymetabolites.org/help#other) | 41454 | Bacilysin biosynthetic gene cluster (100% of genes show similarity) | - |

Table S1. Continued.

| Strain clusters | Type | Length (bp) | Most similar known clusters | Predicted core clusters |
| --- | --- | --- | --- | --- |
| **XH7** | | | | |
| Cluster 1 | [Microcin](http://antismash.secondarymetabolites.org/help#microcin) | 37080 | - | - |
| Cluster 2 | [Microcin](http://antismash.secondarymetabolites.org/help#microcin) | 20148 | - | - |
| Cluster 3 | [Microcin](http://antismash.secondarymetabolites.org/help#microcin) | 26058 | - | - |
| Cluster 4 | [Nrps](http://antismash.secondarymetabolites.org/help#nrps) | 65410 | Surfactin biosynthetic gene cluster (82% of genes show similarity) | - |
| Cluster 5 | Microcin | 20148 | - | - |
| Cluster 6 | [Lantipeptide](http://antismash.secondarymetabolites.org/help#lantipeptide) | 22597 | - | - |
| Cluster 7 | Microcin | 20148 | - | - |
| Cluster 8 | [Otherks](http://antismash.secondarymetabolites.org/help#otherks) | 41244 | - | - |
| Cluster 9 | [Terpene](http://antismash.secondarymetabolites.org/help#terpene) | 20743 | - | - |
| Cluster 10 | [T3pks](http://antismash.secondarymetabolites.org/help#t3pks) | 41109 | - | - |
| Cluster 11 | [Terpene](http://antismash.secondarymetabolites.org/help#terpene) | 21883 | - | - |
| Cluster 12 | [Nrps](http://antismash.secondarymetabolites.org/help#nrps)-[Transatpks](http://antismash.secondarymetabolites.org/help#transatpks) | 110421 | Fengycin biosynthetic gene cluster (93% of genes show similarity) |  |
| Cluster 13 | [Transatpks](http://antismash.secondarymetabolites.org/help#transatpks)-[Nrps](http://antismash.secondarymetabolites.org/help#nrps) | 102704 | Bacillaene biosynthetic gene cluster (100% of genes show similarity) |  |
| Cluster 14 | [Microcin](http://antismash.secondarymetabolites.org/help#microcin) | 20148 | - | - |
| Cluster 15 | [Nrps](http://antismash.secondarymetabolites.org/help#nrps)-[Bacteriocin](http://antismash.secondarymetabolites.org/help#bacteriocin) | 66773 | Bacillibactin biosynthetic gene cluster (100% of genes show similarity) |  |

Table S1. Continued.

| Strain clusters | Type | Length (bp) | Most similar known clusters | Predicted core clusters |
| --- | --- | --- | --- | --- |
| **XH7** | | | | |
| Cluster 16 | [Other](http://antismash.secondarymetabolites.org/help#other) | 41454 | Bacilysin biosynthetic gene cluster (100% of genes show similarity) | - |

Table S1. Continued.

| Strain clusters | Type | Length (bp) | Most similar known clusters | Predicted core clusters |
| --- | --- | --- | --- | --- |
| **JRS8** | | | | |
| Cluster 1 | [Otherks](http://antismash.secondarymetabolites.org/help#otherks) | 32504 | - | - |
| Cluster 2 | [Other](http://antismash.secondarymetabolites.org/help#other) | 41418 | Bacilysin biosynthetic gene cluster (100% of genes show similarity) | - |
| Cluster 3 | [Nrps](http://antismash.secondarymetabolites.org/help#nrps)-[Bacteriocin](http://antismash.secondarymetabolites.org/help#bacteriocin) | 66791 | Bacillibactin biosynthetic gene cluster (100% of genes show similarity) |  |
| Cluster 4 | [Transatpks](http://antismash.secondarymetabolites.org/help#transatpks) | 28181 | Difficidin biosynthetic gene cluster (46% of genes show similarity) |  |
| Cluster 5 | [Terpene](http://antismash.secondarymetabolites.org/help#terpene) | 20740 | - | - |
| Cluster 6 | [Nrps](http://antismash.secondarymetabolites.org/help#nrps)-[Transatpks](http://antismash.secondarymetabolites.org/help#transatpks) | 23873 | Bacillaene biosynthetic gene cluster (64% of genes show similarity) |  |
| Cluster 7 | [Nrps](http://antismash.secondarymetabolites.org/help#nrps) | 9281 | Surfactin biosynthetic gene cluster (8% of genes show similarity) |  |
| Cluster 8 | [Nrps](http://antismash.secondarymetabolites.org/help#nrps) | 25291 | Surfactin biosynthetic gene cluster (39% of genes show similarity) | - |
| Cluster 9 | [Nrps](http://antismash.secondarymetabolites.org/help#nrps)-[Transatpks](http://antismash.secondarymetabolites.org/help#transatpks) | 46647 | - |  |

Table S1. Continued.

| Strain clusters | Type | Length (bp) | Most similar known clusters | Predicted core clusters |
| --- | --- | --- | --- | --- |
| **JRS8** | | | | |
| Cluster 10 | [Nrps](http://antismash.secondarymetabolites.org/help#nrps) | 27863 | Surfactin biosynthetic gene cluster (47% of genes show similarity) |  |
| Cluster 11 | [Nrps](http://antismash.secondarymetabolites.org/help#nrps) | 37131 | Bacillomycin biosynthetic gene cluster (40% of genes show similarity) |  |
| Cluster 12 | [Transatpks](http://antismash.secondarymetabolites.org/help#transatpks) | 85884 | Macrolactin biosynthetic gene cluster (100% of genes show similarity) |  |
| Cluster 13 | [Nrps](http://antismash.secondarymetabolites.org/help#nrps)-[Lantipeptide](http://antismash.secondarymetabolites.org/help#lantipeptide) | 28314 | Locillomycin biosynthetic gene cluster (28% of genes show similarity) |  |
| Cluster 14 | [Transatpks](http://antismash.secondarymetabolites.org/help#transatpks) | 22788 | Difficidin biosynthetic gene cluster (26% of genes show similarity) |  |
| Cluster 15 | [T3pks](http://antismash.secondarymetabolites.org/help#t3pks) | 26337 | - | - |
| Cluster 16 | [Nrps](http://antismash.secondarymetabolites.org/help#nrps) | 3061 | - | - |
| Cluster 17 | [Transatpks](http://antismash.secondarymetabolites.org/help#transatpks) | 35016 | Difficidin biosynthetic gene cluster (53% of genes show similarity) |  |
| Cluster 18 | Terpene | 19126 | - | - |

Table S1. Continued.

| Strain clusters | Type | Length (bp) | Most similar known clusters | Predicted core clusters |
| --- | --- | --- | --- | --- |
| **JRS8** | | | | |
| Cluster 19 | T[ransatpks](http://antismash.secondarymetabolites.org/help#transatpks)-[Nrps](http://antismash.secondarymetabolites.org/help#nrps) | 63018 | Bacillaene biosynthetic gene cluster (35% of genes show similarity) |  |
| Cluster 20 | [Nrps](http://antismash.secondarymetabolites.org/help#nrps) | 21570 | Plipastatin biosynthetic gene cluster (30% of genes show similarity) | - |
| Cluster 21 | [Nrps](http://antismash.secondarymetabolites.org/help#nrps) | 6617 | - |  |
| Cluster 22 | [Nrps](http://antismash.secondarymetabolites.org/help#nrps)-[Transatpks](http://antismash.secondarymetabolites.org/help#transatpks) | 50455 | Fengycin biosynthetic gene cluster (73% of genes show similarity) |  |
| Cluster 23 | [Nrps](http://antismash.secondarymetabolites.org/help#nrps) | 12951 | Fengycin biosynthetic gene cluster (20% of genes show similarity) |  |

Table S1. Continued.

| Strain clusters | Type | Length (bp) | Most similar known clusters | Predicted core clusters |
| --- | --- | --- | --- | --- |
| **JRS8** | | | | |
| Cluster 24 | [Nrps](http://antismash.secondarymetabolites.org/help#nrps) | 9470 | Fengycin biosynthetic gene cluster (13% of genes show similarity) |  |
| Cluster 25 | Transatpks | 2852 | - | - |

Table S1. Continued.

| Strain clusters | Type | Length (bp) | Most similar known clusters | Predicted core clusters |
| --- | --- | --- | --- | --- |
| **629** | | | | |
| Cluster 1 | [Microcin](http://antismash.secondarymetabolites.org/help#microcin) | 20148 | - | - |
| Cluster 2 | [Microcin](http://antismash.secondarymetabolites.org/help#microcin) | 20148 | - | - |
| Cluster 3 | [Microcin](http://antismash.secondarymetabolites.org/help#microcin) -[Lantipeptide](http://antismash.secondarymetabolites.org/help#lantipeptide) | 38414 | - | - |
| Cluster 4 | [Microcin](http://antismash.secondarymetabolites.org/help#microcin) | 26088 | - | - |
| Cluster 5 | Nrps | 65408 | Surfactin biosynthetic gene cluster (91% of genes show similarity) |  |
| Cluster 6 | [Microcin](http://antismash.secondarymetabolites.org/help#microcin) | 20148 | - | - |
| Cluster 7 | [Microcin](http://antismash.secondarymetabolites.org/help#microcin) | 20148 | - | - |
| Cluster 8 | [Otherks](http://antismash.secondarymetabolites.org/help#otherks) | 41244 | Butirosin biosynthetic gene cluster (7% of genes show similarity) | - |
| Cluster 9 | [Terpene](http://antismash.secondarymetabolites.org/help#terpene) | 20740 | - | - |
| Cluster 10 | [Transatpks](http://antismash.secondarymetabolites.org/help#transatpks) | 85900 | Macrolactin biosynthetic gene cluster (100% of genes show similarity) |  |
| Cluster 11 | [Nrps](http://antismash.secondarymetabolites.org/help#nrps)-[Transatpks](http://antismash.secondarymetabolites.org/help#transatpks) | 85922 | Bacillaene biosynthetic gene cluster (92% of genes show similarity) |  |
| Cluster 12 | Nrps-Transatpks | 137831 | Fengycin biosynthetic gene cluster (93% of genes show similarity) |  |

Table S1. Continued.

| Strain clusters | Type | Length (bp) | Most similar known clusters | Predicted core clusters |
| --- | --- | --- | --- | --- |
| **629** | | | | |
| Cluster 13 | [Terpene](http://antismash.secondarymetabolites.org/help#terpene) | 21883 | - | - |
| Cluster 14 | [T3pks](http://antismash.secondarymetabolites.org/help#t3pks) | 41274 | - | - |
| Cluster 15 | [Transatpks](http://antismash.secondarymetabolites.org/help#transatpks) | 94235 | Difficidin biosynthetic gene cluster (100% of genes show similarity) |  |
| Cluster 16 | [Microcin](http://antismash.secondarymetabolites.org/help#microcin) | 25541 | - | - |
| Cluster 17 | Nrps-Bacteriocin | 66787 | Bacillibactin biosynthetic gene cluster (100% of genes show similarity) |  |
| Cluster 18 | [Other](http://antismash.secondarymetabolites.org/help#other) | 41418 | Bacilysin biosynthetic gene cluster (100% of genes show similarity) | - |

Table S1. Continued.

| Strain clusters | Type | Length (bp) | Most similar known clusters | Predicted core clusters |
| --- | --- | --- | --- | --- |
| **M49** | | | | |
| Cluster 1 | [Otherks](http://antismash.secondarymetabolites.org/help#otherks) | 41244 | - | - |
| Cluster 2 | [Terpene](http://antismash.secondarymetabolites.org/help#terpene) | 20740 | - | - |
| Cluster 3 | [Microcin](http://antismash.secondarymetabolites.org/help#microcin) | 1624 | - | - |
| Cluster 4 | [Lantipeptide](http://antismash.secondarymetabolites.org/help#lantipeptide) | 28889 | - | - |
| Cluster 5 | [Terpene](http://antismash.secondarymetabolites.org/help#terpene) | 21883 | - | - |
| Cluster 6 | [T3pks](http://antismash.secondarymetabolites.org/help#t3pks) | 41109 | - | - |
| Cluster 7 | [Transatpks](http://antismash.secondarymetabolites.org/help#transatpks) | 45758 | Difficidin biosynthetic gene cluster (53% of genes show similarity) |  |
| Cluster 8 | [Nrps](http://antismash.secondarymetabolites.org/help#nrps) | 9123 | Surfactin biosynthetic gene cluster (8% of genes show similarity) |  |
| Cluster 9 | [Nrps](http://antismash.secondarymetabolites.org/help#nrps) | 25294 | Surfactin biosynthetic gene cluster (39% of genes show similarity) | - |
| Cluster 10 | [Other](http://antismash.secondarymetabolites.org/help#other) | 41418 | Bacilysin biosynthetic gene cluster (85% of genes show similarity) | - |
| Cluster 11 | [Transatpks](http://antismash.secondarymetabolites.org/help#transatpks) | 22789 | Difficidin biosynthetic gene cluster (26% of genes show similarity) |  |
| Cluster 12 | [Nrps](http://antismash.secondarymetabolites.org/help#nrps) | 13783 | Fengycin biosynthetic gene cluster (20% of genes show similarity) |  |

Table S1. Continued.

| Strain clusters | Type | Length (bp) | Most similar known clusters | Predicted core clusters |
| --- | --- | --- | --- | --- |
| **M49** | | | | |
| Cluster 13 | [Nrps](http://antismash.secondarymetabolites.org/help#nrps) | 1651 | - | - |
| Cluster 14 | [Nrps](http://antismash.secondarymetabolites.org/help#nrps) | 22443 | Locillomycin biosynthetic gene cluster (35% of genes show similarity) | - |
| Cluster 15 | Other | 1694 | - | - |
| Cluster 16 | [Nrps](http://antismash.secondarymetabolites.org/help#nrps) | 9364 | Fengycin biosynthetic gene cluster (13% of genes show similarity) |  |
| Cluster 17 | [Nrps](http://antismash.secondarymetabolites.org/help#nrps) | 6556 | - |  |
| Cluster 18 | [Bacteriocin](http://antismash.secondarymetabolites.org/help#bacteriocin)-[Nrps](http://antismash.secondarymetabolites.org/help#nrps) | 50488 | Bacillibactin biosynthetic gene cluster (92% of genes show similarity) |  |
| Cluster 19 | [Transatpks](http://antismash.secondarymetabolites.org/help#transatpks) | 28137 | Difficidin biosynthetic gene cluster (46% of genes show similarity) |  |
| Cluster 20 | [Transatpks](http://antismash.secondarymetabolites.org/help#transatpks) | 85884 | Macrolactin biosynthetic gene cluster (100% of genes show similarity) |  |

Table S1. Continued.

| Strain clusters | Type | Length (bp) | Most similar known clusters | Predicted core clusters |
| --- | --- | --- | --- | --- |
| **M49** | | | | |
| Cluster 21 | [Nrps](http://antismash.secondarymetabolites.org/help#nrps)-[Transatpks](http://antismash.secondarymetabolites.org/help#transatpks) | 102701 | Bacillaene biosynthetic gene cluster (100% of genes show similarity) |  |
| Cluster 22 | [Nrps](http://antismash.secondarymetabolites.org/help#nrps)-[Transatpks](http://antismash.secondarymetabolites.org/help#transatpks) | 87621 | Fengycin biosynthetic gene cluster (80% of genes show similarity) |  |
| Cluster 23 | [Nrps](http://antismash.secondarymetabolites.org/help#nrps)-[Transatpks](http://antismash.secondarymetabolites.org/help#transatpks) | 46603 | - |  |
| Cluster 24 | [Nrps](http://antismash.secondarymetabolites.org/help#nrps) | 27702 | Surfactin biosynthetic gene cluster (47% of genes show similarity) |  |

Table S1. Continued.

| Strain clusters | Type | Length (bp) | Most similar known clusters | Predicted core clusters |
| --- | --- | --- | --- | --- |
| **X1** | | | | |
| Cluster 1 | [Nrps](http://antismash.secondarymetabolites.org/help#nrps) | 2356 | - | - |
| Cluster 2 | [Microcin](http://antismash.secondarymetabolites.org/help#microcin) | 4582 | - | - |
| Cluster 3 | [Microcin](http://antismash.secondarymetabolites.org/help#microcin) | 4466 | - | - |
| Cluster 4 | [Nrps](http://antismash.secondarymetabolites.org/help#nrps) | 1944 | - | - |
| Cluster 5 | [Transatpks](http://antismash.secondarymetabolites.org/help#transatpks) | 25798 | Difficidin biosynthetic gene cluster (26% of genes show similarity) |  |
| Cluster 6 | [Nrps](http://antismash.secondarymetabolites.org/help#nrps) | 12004 | Surfactin biosynthetic gene cluster (8% of genes show similarity) |  |
| Cluster 7 | [Nrps](http://antismash.secondarymetabolites.org/help#nrps) | 14294 | Fengycin biosynthetic gene cluster (26% of genes show similarity) |  |
| Cluster 8 | [Nrps](http://antismash.secondarymetabolites.org/help#nrps) | 11099 | Fengycin biosynthetic gene cluster (20% of genes show similarity) |  |

Table S1. Continued.

| Strain clusters | Type | Length (bp) | Most similar known clusters | Predicted core clusters |
| --- | --- | --- | --- | --- |
| **X1** | | | | |
| Cluster 9 | [Nrps](http://antismash.secondarymetabolites.org/help#nrps) | 29214 | Surfactin biosynthetic gene cluster (52% of genes show similarity) |  |
| Cluster 10 | [Nrps](http://antismash.secondarymetabolites.org/help#nrps) | 26663 | Surfactin biosynthetic gene cluster (39% of genes show similarity) |  |
| Cluster 11 | [Transatpks](http://antismash.secondarymetabolites.org/help#transatpks) | 46880 | Difficidin biosynthetic gene cluster (46% of genes show similarity) | - |
| Cluster 12 | [T3pks](http://antismash.secondarymetabolites.org/help#t3pks) | 41109 | - | - |
| Cluster 13 | [Terpene](http://antismash.secondarymetabolites.org/help#terpene) | 21883 | - | - |
| Cluster 14 | [Nrps](http://antismash.secondarymetabolites.org/help#nrps) | 22383 | Plipastatin biosynthetic gene cluster (30% of genes show similarity) | - |
| Cluster 15 | [Transatpks](http://antismash.secondarymetabolites.org/help#transatpks) | 30033 | Difficidin biosynthetic gene cluster (46% of genes show similarity) |  |
| Cluster 16 | Otherks | 41244 | - | - |
| Cluster 17 | [Terpene](http://antismash.secondarymetabolites.org/help#terpene) | 20740 | - | - |
| Cluster 18 | [Lantipeptide](http://antismash.secondarymetabolites.org/help#lantipeptide) | 28888 | - | - |

Table S1. Continued.

| Strain clusters | Type | Length (bp) | Most similar known clusters | Predicted core clusters |
| --- | --- | --- | --- | --- |
| **X1** | | | | |
| Cluster 19 | [Transatpks](http://antismash.secondarymetabolites.org/help#transatpks) | 85905 | Macrolactin biosynthetic gene cluster (100% of genes show similarity) |  |
| Cluster 20 | [Nrps](http://antismash.secondarymetabolites.org/help#nrps)-[Transatpks](http://antismash.secondarymetabolites.org/help#transatpks) | 102674 | Bacillaene biosynthetic gene cluster (100% of genes show similarity) |  |
| Cluster 21 | [Nrps](http://antismash.secondarymetabolites.org/help#nrps)-[Transatpks](http://antismash.secondarymetabolites.org/help#transatpks) | 87870 | Fengycin biosynthetic gene cluster (80% of genes show similarity) |  |
| Cluster 22 | [Other](http://antismash.secondarymetabolites.org/help#other) | 41418 | Bacilysin biosynthetic gene cluster (85% of genes show similarity) | - |
| Cluster 23 | [Bacteriocin](http://antismash.secondarymetabolites.org/help#bacteriocin)-[Nrps](http://antismash.secondarymetabolites.org/help#nrps) | 51791 | Bacillibactin biosynthetic gene cluster (92% of genes show similarity) |  |

Table S1. Continued.

| Strain clusters | Type | Length (bp) | Most similar known clusters | Predicted core clusters |
| --- | --- | --- | --- | --- |
| **JRS5** | | | | |
| Cluster 1 | [Microcin](http://antismash.secondarymetabolites.org/help#microcin) | 1361 | - | - |
| Cluster 2 | [Nrps](http://antismash.secondarymetabolites.org/help#nrps)-[Bacteriocin](http://antismash.secondarymetabolites.org/help#bacteriocin) | 47595 | Bacillibactin biosynthetic gene cluster (100% of genes show similarity) |  |
| Cluster 3 | Lantipeptide | 44806 | Mersacidin biosynthetic gene cluster (90% of genes show similarity) | - |
| Cluster 4 | [Transatpks](http://antismash.secondarymetabolites.org/help#transatpks) | 22108 | - | - |
| Cluster 5 | [Other](http://antismash.secondarymetabolites.org/help#other) | 41418 | Bacilysin biosynthetic gene cluster (85% of genes show similarity) | - |
| Cluster 6 | [Nrps](http://antismash.secondarymetabolites.org/help#nrps)-[Transatpks](http://antismash.secondarymetabolites.org/help#transatpks) | 44032 | Bacillaene biosynthetic gene cluster (78% of genes show similarity) |  |
| Cluster 7 | [Terpene](http://antismash.secondarymetabolites.org/help#terpene) | 20740 | - | - |
| Cluster 8 | [T3pks](http://antismash.secondarymetabolites.org/help#t3pks) | 37150 | - | - |
| Cluster 9 | [Nrps](http://antismash.secondarymetabolites.org/help#nrps) | 22460 | Locillomycin biosynthetic gene cluster (35% of genes show similarity) | - |
| Cluster 10 | [Phosphonate](http://antismash.secondarymetabolites.org/help#phosphonate) | 40902 | Pactamycin biosynthetic gene cluster (3% of genes show similarity) | - |
| Cluster 11 | [Nrps](http://antismash.secondarymetabolites.org/help#nrps) | 25495 | Surfactin biosynthetic gene cluster (39% of genes show similarity) | - |
| Cluster 12 | [Nrps](http://antismash.secondarymetabolites.org/help#nrps)-[Transatpks](http://antismash.secondarymetabolites.org/help#transatpks) | 33841 | - |  |
| Cluster 13 | [Transatpks](http://antismash.secondarymetabolites.org/help#transatpks) | 68478 | Macrolactin biosynthetic gene cluster (100% of genes show similarity) |  |

Table S1. Continued.

| Strain clusters | Type | Length (bp) | Most similar known clusters | Predicted core clusters |
| --- | --- | --- | --- | --- |
| **JRS5** | | | | |
| Cluster 14 | [Nrps](http://antismash.secondarymetabolites.org/help#nrps) | 27663 | Surfactin biosynthetic gene cluster (47% of genes show similarity) |  |
| Cluster 15 | [Otherks](http://antismash.secondarymetabolites.org/help#otherks) | 36332 | Butirosin biosynthetic gene cluster (7% of genes show similarity) | - |
| Cluster 16 | [Nrps](http://antismash.secondarymetabolites.org/help#nrps)-[Transatpks](http://antismash.secondarymetabolites.org/help#transatpks) | 87674 | Fengycin biosynthetic gene cluster (73% of genes show similarity) |  |
| Cluster 17 | [Transatpks](http://antismash.secondarymetabolites.org/help#transatpks) | 22868 | Difficidin biosynthetic gene cluster (26% of genes show similarity) |  |
| Cluster 18 | Nrps | 9285 | Surfactin biosynthetic gene cluster (8% of genes show similarity) |  |

Table S1. Continued.

| Strain clusters | Type | Length (bp) | Most similar known clusters | Predicted core clusters |
| --- | --- | --- | --- | --- |
| **JRS5** | | | | |
| Cluster 19 | [Nrps](http://antismash.secondarymetabolites.org/help#nrps) | 3088 | - | - |
| Cluster 20 | [Transatpks](http://antismash.secondarymetabolites.org/help#transatpks) | 45658 | Difficidin biosynthetic gene cluster (46% of genes show similarity) |  |
| Cluster 21 | [Transatpks](http://antismash.secondarymetabolites.org/help#transatpks) | 5708 | - | - |
| Cluster 22 | [Terpene](http://antismash.secondarymetabolites.org/help#terpene) | 14768 | - | - |
| Cluster 23 | [Transatpks](http://antismash.secondarymetabolites.org/help#transatpks)-[Nrps](http://antismash.secondarymetabolites.org/help#nrps) | 50924 | Bacillaene biosynthetic gene cluster (35% of genes show similarity) |  |
| Cluster 24 | [Nrps](http://antismash.secondarymetabolites.org/help#nrps) | 20753 | Plipastatin biosynthetic gene cluster (23% of genes show similarity) | - |
| Cluster 25 | [Nrps](http://antismash.secondarymetabolites.org/help#nrps) | 6645 | - |  |
| Cluster 26 | [Nrps](http://antismash.secondarymetabolites.org/help#nrps) | 13050 | Fengycin biosynthetic gene cluster (20% of genes show similarity) |  |

Table S1. Continued.

| Strain clusters | Type | Length (bp) | Most similar known clusters | Predicted core clusters |
| --- | --- | --- | --- | --- |
| **JRS5** | | | | |
| Cluster 27 | [Nrps](http://antismash.secondarymetabolites.org/help#nrps) | 9306 | Fengycin biosynthetic gene cluster (13% of genes show similarity) |  |

Table S1. Continued.

| Strain clusters | Type | Length (bp) | Most similar known clusters | Predicted core clusters |
| --- | --- | --- | --- | --- |
| **EGD-AQ14** | | | | |
| Cluster 1 | [Nrps](http://antismash.secondarymetabolites.org/help#nrps)-[Transatpks](http://antismash.secondarymetabolites.org/help#transatpks) | 87762 | Fengycin biosynthetic gene cluster (80% of genes show similarity) |  |
| Cluster 2 | [Transatpks](http://antismash.secondarymetabolites.org/help#transatpks)-[Nrps](http://antismash.secondarymetabolites.org/help#nrps) | 102692 | Bacillaene biosynthetic gene cluster (92% of genes show similarity) |  |
| Cluster 3 | [Transatpks](http://antismash.secondarymetabolites.org/help#transatpks) | 85902 | Macrolactin biosynthetic gene cluster (90% of genes show similarity) |  |
| Cluster 4 | Terpene | 21883 | - | - |
| Cluster 5 | [Nrps](http://antismash.secondarymetabolites.org/help#nrps) | 40909 | - |  |
| Cluster 6 | Nrps | 27617 | Surfactin biosynthetic gene cluster (47% of genes show similarity) |  |

Table S1. Continued.

| Strain clusters | Type | Length (bp) | Most similar known clusters | Predicted core clusters |
| --- | --- | --- | --- | --- |
| **EGD-AQ14** | | | | |
| Cluster 7 | [T3pks](http://antismash.secondarymetabolites.org/help#t3pks) | 41109 | - | - |
| Cluster 8 | [Transatpks](http://antismash.secondarymetabolites.org/help#transatpks) | 100447 | Difficidin biosynthetic gene cluster (100% of genes show similarity) |  |
| Cluster 9 | [Nrps](http://antismash.secondarymetabolites.org/help#nrps) | 12807 | Fengycin biosynthetic gene cluster (33% of genes show similarity) |  |
| Cluster 10 | [Nrps](http://antismash.secondarymetabolites.org/help#nrps) | 10585 | Fengycin biosynthetic gene cluster (20% of genes show similarity) |  |
| Cluster 11 | [Nrps](http://antismash.secondarymetabolites.org/help#nrps) | 9279 | Surfactin biosynthetic gene cluster (8% of genes show similarity) |  |
| Cluster 12 | [Other](http://antismash.secondarymetabolites.org/help#other) | 41418 | Bacilysin biosynthetic gene cluster (85% of genes show similarity) | - |
| Cluster 13 | [Nrps](http://antismash.secondarymetabolites.org/help#nrps) | 2153 | - | - |
| Cluster 14 | [Nrps](http://antismash.secondarymetabolites.org/help#nrps) | 2152 | - | - |
| Cluster 15 | [Microcin](http://antismash.secondarymetabolites.org/help#microcin) | 1713 | - | - |
| Cluster 16 | [Otherks](http://antismash.secondarymetabolites.org/help#otherks) | 41244 | - | - |
| Cluster 17 | Terpene | 20740 | - | - |

Table S1. Continued.

| Strain clusters | Type | Length (bp) | Most similar known clusters | Predicted core clusters |
| --- | --- | --- | --- | --- |
| **EGD-AQ14** | | | | |
| Cluster 18 | [Nrps](http://antismash.secondarymetabolites.org/help#nrps) | 25535 | Surfactin biosynthetic gene cluster (39% of genes show similarity) | - |
| Cluster 19 | [Phosphonate](http://antismash.secondarymetabolites.org/help#phosphonate) | 40902 | Pactamycin biosynthetic gene cluster (3% of genes show similarity) | - |
| Cluster 20 | L[antipeptide](http://antismash.secondarymetabolites.org/help#lantipeptide) | 23779 | - | - |
| Cluster 21 | [Nrps](http://antismash.secondarymetabolites.org/help#nrps) | 23507 | - | - |
| Cluster 22 | [Nrps](http://antismash.secondarymetabolites.org/help#nrps)-[Bacteriocin](http://antismash.secondarymetabolites.org/help#bacteriocin) | 51539 | Bacillibactin biosynthetic gene cluster (100% of genes show similarity) |  |

Table S1. Continued.

| Strain clusters | Type | Length (bp) | Most similar known clusters | Predicted core clusters |
| --- | --- | --- | --- | --- |
| **LPL-K103** | | | | |
| Cluster 1 | [Microcin](http://antismash.secondarymetabolites.org/help#microcin) | 20148 | - | - |
| Cluster 2 | [Nrps](http://antismash.secondarymetabolites.org/help#nrps) | 17322 | Surfactin biosynthetic gene cluster (47% of genes show similarity) |  |
| Cluster 3 | [Nrps](http://antismash.secondarymetabolites.org/help#nrps) | 10184 | Surfactin biosynthetic gene cluster (8% of genes show similarity) |  |
| Cluster 4 | [Nrps](http://antismash.secondarymetabolites.org/help#nrps) | 25553 | Surfactin biosynthetic gene cluster (47% of genes show similarity) | - |
| Cluster 5 | [Microcin](http://antismash.secondarymetabolites.org/help#microcin) | 20148 | - | - |
| Cluster 6 | [Otherks](http://antismash.secondarymetabolites.org/help#otherks) | 41244 | Butirosin biosynthetic gene cluster (7% of genes show similarity) | - |
| Cluster 7 | [Terpene](http://antismash.secondarymetabolites.org/help#terpene) | 20740 | - | - |
| Cluster 8 | Transatpks | 85901 | Macrolactin biosynthetic gene cluster (100% of genes show similarity) |  |
| Cluster 9 | [Nrps](http://antismash.secondarymetabolites.org/help#nrps)-[Transatpks](http://antismash.secondarymetabolites.org/help#transatpks) | 102695 | Bacillaene biosynthetic gene cluster (100% of genes show similarity) |  |

Table S1. Continued.

| Strain clusters | Type | Length (bp) | Most similar known clusters | Predicted core clusters |
| --- | --- | --- | --- | --- |
| **LPL-K103** | | | | |
| Cluster 10 | [Nrps](http://antismash.secondarymetabolites.org/help#nrps)-[Transatpks](http://antismash.secondarymetabolites.org/help#transatpks) | 87760 | Fengycin biosynthetic gene cluster (80% of genes show similarity) |  |
| Cluster 11 | [Nrps](http://antismash.secondarymetabolites.org/help#nrps) | 9820 | Fengycin biosynthetic gene cluster (13% of genes show similarity) |  |
| Cluster 12 | [Nrps](http://antismash.secondarymetabolites.org/help#nrps) | 13217 | Fengycin biosynthetic gene cluster (20% of genes show similarity) |  |

Table S1. Continued.

| Strain clusters | Type | Length (bp) | Most similar known clusters | Predicted core clusters |
| --- | --- | --- | --- | --- |
| **LPL-K103** | | | | |
| Cluster 13 | [Nrps](http://antismash.secondarymetabolites.org/help#nrps) | 21732 | Plipastatin biosynthetic gene cluster (23% of genes show similarity) | - |
| Cluster 14 | [Terpene](http://antismash.secondarymetabolites.org/help#terpene) | 21883 | - | - |
| Cluster 15 | [T3pks](http://antismash.secondarymetabolites.org/help#t3pks) | 41109 | - | - |
| Cluster 16 | [Transatpks](http://antismash.secondarymetabolites.org/help#transatpks) | 100444 | Difficidin biosynthetic gene cluster (100% of genes show similarity) |  |
| Cluster 17 | [Nrps](http://antismash.secondarymetabolites.org/help#nrps) | 2766 | - | - |
| Cluster 18 | [Nrps](http://antismash.secondarymetabolites.org/help#nrps)-[Bacteriocin](http://antismash.secondarymetabolites.org/help#bacteriocin) | 66793 | Bacillibactin biosynthetic gene cluster (100% of genes show similarity) |  |
| Cluster 19 | [Other](http://antismash.secondarymetabolites.org/help#other) | 41418 | Bacilysin biosynthetic gene cluster (100% of genes show similarity) | - |

Table S1. Continued.

| Strain clusters | Type | Length (bp) | Most similar known clusters | Predicted core clusters |
| --- | --- | --- | --- | --- |
| **B4140** | | | | |
| Cluster 1 | [Nrps](http://antismash.secondarymetabolites.org/help#nrps)-[Transatpks](http://antismash.secondarymetabolites.org/help#transatpks) | 88248 | Fengycin biosynthetic gene cluster (86% of genes show similarity) |  |
| Cluster 2 | [Transatpks](http://antismash.secondarymetabolites.org/help#transatpks)-[Nrps](http://antismash.secondarymetabolites.org/help#nrps) | 102698 | Bacillaene biosynthetic gene cluster (92% of genes show similarity) |  |
| Cluster 3 | [Bacteriocin](http://antismash.secondarymetabolites.org/help#bacteriocin)-[Nrps](http://antismash.secondarymetabolites.org/help#nrps) | 51792 | Bacillibactin biosynthetic gene cluster (92% of genes show similarity) |  |
| Cluster 4 | [Microcin](http://antismash.secondarymetabolites.org/help#microcin) | 1572 | - | - |
| Cluster 5 | [Nrps](http://antismash.secondarymetabolites.org/help#nrps) | 9350 | Fengycin biosynthetic gene cluster (13% of genes show similarity) |  |
| Cluster 6 | [Terpene](http://antismash.secondarymetabolites.org/help#terpene) | 21883 | - | - |
| Cluster 7 | [Transatpks](http://antismash.secondarymetabolites.org/help#transatpks) | 85887 | Macrolactin biosynthetic gene cluster (100% of genes show similarity) |  |

Table S1. Continued.

| Strain clusters | Type | Length (bp) | Most similar known clusters | Predicted core clusters |
| --- | --- | --- | --- | --- |
| **B4140** | | | | |
| Cluster 8 | [Other](http://antismash.secondarymetabolites.org/help#other) | 41418 | Bacilysin biosynthetic gene cluster (85% of genes show similarity) | - |
| Cluster 9 | [Nrps](http://antismash.secondarymetabolites.org/help#nrps) | 22196 | Plipastatin biosynthetic gene cluster (46% of genes show similarity) | - |
| Cluster 10 | [Nrps](http://antismash.secondarymetabolites.org/help#nrps) | 1072 | - | - |
| Cluster 11 | [Nrps](http://antismash.secondarymetabolites.org/help#nrps) | 14139 | Fengycin biosynthetic gene cluster (26% of genes show similarity) |  |
| Cluster 12 | [Nrps](http://antismash.secondarymetabolites.org/help#nrps) | 10816 | Surfactin biosynthetic gene cluster (8% of genes show similarity) |  |
| Cluster 13 | [Nrps](http://antismash.secondarymetabolites.org/help#nrps) | 54641 | Surfactin biosynthetic gene cluster (91% of genes show similarity) |  |

Table S1. Continued.

| Strain clusters | Type | Length (bp) | Most similar known clusters | Predicted core clusters |
| --- | --- | --- | --- | --- |
| **B4140** | | | | |
| Cluster 14 | [Otherks](http://antismash.secondarymetabolites.org/help#otherks) | 41244 | - | - |
| Cluster 15 | [Terpene](http://antismash.secondarymetabolites.org/help#terpene) | 20740 | - | - |
| Cluster 16 | [Nrps](http://antismash.secondarymetabolites.org/help#nrps)-[Transatpks](http://antismash.secondarymetabolites.org/help#transatpks) | 113031 | Calyculin biosynthetic gene cluster (16% of genes show similarity) |  |
| Cluster 17 | [Transatpks](http://antismash.secondarymetabolites.org/help#transatpks) | 100120 | Difficidin biosynthetic gene cluster (93% of genes show similarity) |  |
| Cluster 18 | [T3pks](http://antismash.secondarymetabolites.org/help#t3pks) | 41109 | - | - |

Table S1. Continued.

| Strain clusters | Type | Length (bp) | Most similar known clusters | Predicted core clusters |
| --- | --- | --- | --- | --- |
| **HB-26** | | | | |
| Cluster 1 | [Transatpks](http://antismash.secondarymetabolites.org/help#transatpks) | 28303 | Difficidin biosynthetic gene cluster (46% of genes show similarity) |  |
| Cluster 2 | [Other](http://antismash.secondarymetabolites.org/help#other) | 41418 | Bacilysin biosynthetic gene cluster (85% of genes show similarity) | - |
| Cluster 3 | [Otherks](http://antismash.secondarymetabolites.org/help#otherks) | 41244 | Butirosin biosynthetic gene cluster (7% of genes show similarity) | - |
| Cluster 4 | [Terpene](http://antismash.secondarymetabolites.org/help#terpene) | 20740 | - | - |
| Cluster 5 | [Transatpks](http://antismash.secondarymetabolites.org/help#transatpks) | 85893 | Macrolactin biosynthetic gene cluster (90% of genes show similarity) |  |
| Cluster 6 | [Nrps](http://antismash.secondarymetabolites.org/help#nrps) | 21644 | Plipastatin biosynthetic gene cluster (23% of genes show similarity) | - |
| Cluster 7 | [Terpene](http://antismash.secondarymetabolites.org/help#terpene) | 21883 | - | - |
| Cluster 8 | [T3pks](http://antismash.secondarymetabolites.org/help#t3pks) | 41109 | - | - |
| Cluster 9 | [Transatpks](http://antismash.secondarymetabolites.org/help#transatpks) | 45702 | Difficidin biosynthetic gene cluster (53% of genes show similarity) |  |
| Cluster 10 | [Nrps](http://antismash.secondarymetabolites.org/help#nrps)-[Transatpks](http://antismash.secondarymetabolites.org/help#transatpks) | 102704 | Bacillaene biosynthetic gene cluster (100% of genes show similarity) |  |
| Cluster 11 | [Nrps](http://antismash.secondarymetabolites.org/help#nrps)-[Transatpks](http://antismash.secondarymetabolites.org/help#transatpks) | 87835 | Fengycin biosynthetic gene cluster (80% of genes show similarity) |  |

Table S1. Continued.

| Strain clusters | Type | Length (bp) | Most similar known clusters | Predicted core clusters |
| --- | --- | --- | --- | --- |
| **HB-26** | | | | |
| Cluster 12 | [Bacteriocin](http://antismash.secondarymetabolites.org/help#bacteriocin)-[Nrps](http://antismash.secondarymetabolites.org/help#nrps) | 51793 | Bacillibactin biosynthetic gene cluster (92% of genes show similarity) |  |
| Cluster 13 | Nrps | 25561 | Surfactin biosynthetic gene cluster (47% of genes show similarity) | - |
| Cluster 14 | [Nrps](http://antismash.secondarymetabolites.org/help#nrps)-[Transatpks](http://antismash.secondarymetabolites.org/help#transatpks) | 70182 | Locillomycin biosynthetic gene cluster (35% of genes show similarity) |  |
| Cluster 15 | [Nrps](http://antismash.secondarymetabolites.org/help#nrps) | 27593 | Surfactin biosynthetic gene cluster (47% of genes show similarity) |  |
| Cluster 16 | [Nrps](http://antismash.secondarymetabolites.org/help#nrps) | 23168 | - | - |
| Cluster 17 | [Transatpks](http://antismash.secondarymetabolites.org/help#transatpks) | 24667 | Kalimantacin / batumin biosynthetic gene cluster (6% of genes show similarity) |  |
| Cluster 18 | [Nrps](http://antismash.secondarymetabolites.org/help#nrps) | 14929 | Fengycin biosynthetic gene cluster (26% of genes show similarity) |  |

Table S1. Continued.

| Strain clusters | Type | Length (bp) | Most similar known clusters | Predicted core clusters |
| --- | --- | --- | --- | --- |
| **HB-26** | | | | |
| Cluster 19 | [Nrps](http://antismash.secondarymetabolites.org/help#nrps) | 10854 | Surfactin biosynthetic gene cluster (8% of genes show similarity) |  |
| Cluster 20 | [Nrps](http://antismash.secondarymetabolites.org/help#nrps) | 10123 | Fengycin biosynthetic gene cluster (20% of genes show similarity) |  |
| Cluster 21 | [Nrps](http://antismash.secondarymetabolites.org/help#nrps) | 8226 | - |  |
| Cluster 22 | [Nrps](http://antismash.secondarymetabolites.org/help#nrps) | 6991 | - | - |
| Cluster 23 | [Nrps](http://antismash.secondarymetabolites.org/help#nrps) | 6505 | - |  |
| Cluster 24 | [Microcin](http://antismash.secondarymetabolites.org/help#microcin) | 4832 | - | - |
| Cluster 25 | [Nrps](http://antismash.secondarymetabolites.org/help#nrps) | 2559 | - | - |

Table S1. Continued.

| Strain clusters | Type | Length (bp) | Most similar known clusters | Predicted core clusters |
| --- | --- | --- | --- | --- |
| **11B91** | | | | |
| Cluster 1 | [Nrps](http://antismash.secondarymetabolites.org/help#nrps) | 1075 | - | - |
| Cluster 2 | [Microcin](http://antismash.secondarymetabolites.org/help#microcin) | 5916 | - | - |
| Cluster 3 | [Transatpks](http://antismash.secondarymetabolites.org/help#transatpks) | 100445 | Difficidin biosynthetic gene cluster (93% of genes show similarity) |  |
| Cluster 4 | [T3pks](http://antismash.secondarymetabolites.org/help#t3pks) | 41109 | - | - |
| Cluster 5 | [Terpene](http://antismash.secondarymetabolites.org/help#terpene) | 21883 | - | - |
| Cluster 6 | [Nrps](http://antismash.secondarymetabolites.org/help#nrps)-[Transatpks](http://antismash.secondarymetabolites.org/help#transatpks) | 138382 | Fengycin biosynthetic gene cluster (100% of genes show similarity) |  |
| Cluster 7 | [Transatpks](http://antismash.secondarymetabolites.org/help#transatpks)-[Nrps](http://antismash.secondarymetabolites.org/help#nrps) | 102692 | Bacillaene biosynthetic gene cluster (92% of genes show similarity) |  |
| Cluster 8 | [Transatpks](http://antismash.secondarymetabolites.org/help#transatpks) | 85896 | Macrolactin biosynthetic gene cluster (90% of genes show similarity) | - |
| Cluster 9 | [Terpene](http://antismash.secondarymetabolites.org/help#terpene) | 20740 | - | - |
| Cluster 10 | [Otherks](http://antismash.secondarymetabolites.org/help#otherks) | 41244 | Butirosin biosynthetic gene cluster (7% of genes show similarity) | - |
| Cluster 11 | [Other](http://antismash.secondarymetabolites.org/help#other) | 41418 | Bacilysin biosynthetic gene cluster (85% of genes show similarity) | - |

Table S1. Continued.

| Strain clusters | Type | Length (bp) | Most similar known clusters | Predicted core clusters |
| --- | --- | --- | --- | --- |
| **11B91** | | | | |
| Cluster 12 | [Bacteriocin](http://antismash.secondarymetabolites.org/help#bacteriocin)-[Nrps](http://antismash.secondarymetabolites.org/help#nrps) | 51794 | Bacillibactin biosynthetic gene cluster (92% of genes show similarity) |  |
| Cluster 13 | [Nrps](http://antismash.secondarymetabolites.org/help#nrps)-[Transatpks](http://antismash.secondarymetabolites.org/help#transatpks) | 77757 | Rhizocticin biosynthetic gene cluster (19% of genes show similarity) |  |
| Cluster 14 | [Nrps](http://antismash.secondarymetabolites.org/help#nrps) | 65493 | Surfactin biosynthetic gene cluster (91% of genes show similarity) |  |

Table S1. Continued.

| Strain clusters | Type | Length (bp) | Most similar known clusters | Predicted core clusters |
| --- | --- | --- | --- | --- |
| **Jxnuwx-1** | | | | |
| Cluster 1 | [Nrps](http://antismash.secondarymetabolites.org/help#nrps)-[Transatpks](http://antismash.secondarymetabolites.org/help#transatpks) | 88243 | Fengycin biosynthetic gene cluster (86% of genes show similarity) |  |
| Cluster 2 | [Transatpks](http://antismash.secondarymetabolites.org/help#transatpks)-[Nrps](http://antismash.secondarymetabolites.org/help#nrps) | 102704 | Bacillaene biosynthetic gene cluster (92% of genes show similarity) |  |
| Cluster 3 | [Transatpks](http://antismash.secondarymetabolites.org/help#transatpks) | 85881 | Macrolactin biosynthetic gene cluster (90% of genes show similarity) |  |
| Cluster 4 | [Transatpks](http://antismash.secondarymetabolites.org/help#transatpks) | 70770 | Difficidin biosynthetic gene cluster (60% of genes show similarity) | - |
| Cluster 5 | [T3pks](http://antismash.secondarymetabolites.org/help#t3pks) | 41109 | - | - |
| Cluster 6 | [Terpene](http://antismash.secondarymetabolites.org/help#terpene) | 21883 | - | - |
| Cluster 7 | [Nrps](http://antismash.secondarymetabolites.org/help#nrps) | 22039 | Plipastatin biosynthetic gene cluster (30% of genes show similarity) | - |
| Cluster 8 | [Terpene](http://antismash.secondarymetabolites.org/help#terpene) | 20740 | - | - |
| Cluster 9 | [Nrps](http://antismash.secondarymetabolites.org/help#nrps) | 25370 | Surfactin biosynthetic gene cluster (39% of genes show similarity) | - |
| Cluster 10 | [Nrps](http://antismash.secondarymetabolites.org/help#nrps) | 22392 | Surfactin biosynthetic gene cluster (43% of genes show similarity) | - |
| Cluster 11 | [Thiopeptide](http://antismash.secondarymetabolites.org/help#thiopeptide)-[Lantipeptide](http://antismash.secondarymetabolites.org/help#lantipeptide) | 30901 | Kijanimicin biosynthetic gene cluster (4% of genes show similarity) |  |

Table S1. Continued.

| Strain clusters | Type | Length (bp) | Most similar known clusters | Predicted core clusters |
| --- | --- | --- | --- | --- |
| **Jxnuwx-1** | | | | |
| Cluster 12 | [Other](http://antismash.secondarymetabolites.org/help#other) | 21523 | Bacillibactin biosynthetic gene cluster (30% of genes show similarity) | - |
| Cluster 13 | [Other](http://antismash.secondarymetabolites.org/help#other) | 25016 | Bacilysin biosynthetic gene cluster (100% of genes show similarity) | - |
| Cluster 14 | Bacteriocin-Nrps | 30290 | Bacillibactin biosynthetic gene cluster (76% of genes show similarity) |  |
| Cluster 15 | [Transatpks](http://antismash.secondarymetabolites.org/help#transatpks) | 14565 | Difficidin biosynthetic gene cluster (46% of genes show similarity) |  |
| Cluster 16 | [Nrps](http://antismash.secondarymetabolites.org/help#nrps) | 13399 | Fengycin biosynthetic gene cluster (26% of genes show similarity) |  |
| Cluster 17 | [Nrps](http://antismash.secondarymetabolites.org/help#nrps) | 9535 | Surfactin biosynthetic gene cluster (8% of genes show similarity) |  |

Table S1. Continued.

| Strain clusters | Type | Length (bp) | Most similar known clusters | Predicted core clusters |
| --- | --- | --- | --- | --- |
| **Jxnuwx-1** | | | | |
| Cluster 18 | [Nrps](http://antismash.secondarymetabolites.org/help#nrps) | 9352 | Fengycin biosynthetic gene cluster (13% of genes show similarity) |  |
| Cluster 19 | [Nrps](http://antismash.secondarymetabolites.org/help#nrps) | 5312 | - | - |
| Cluster 20 | [Microcin](http://antismash.secondarymetabolites.org/help#microcin) | 1583 | - | - |

Table S1. Continued.

| Strain clusters | Type | Length (bp) | Most similar known clusters | Predicted core clusters |
| --- | --- | --- | --- | --- |
| **Lx-11** | | | | |
| Cluster 1 | [Other](http://antismash.secondarymetabolites.org/help#other) | 41418 | Bacilysin biosynthetic gene cluster (85% of genes show similarity) | - |
| Cluster 2 | [Bacteriocin](http://antismash.secondarymetabolites.org/help#bacteriocin)-[Nrps](http://antismash.secondarymetabolites.org/help#nrps) | 48791 | Bacillibactin biosynthetic gene cluster (92% of genes show similarity) |  |
| Cluster 3 | [Transatpks](http://antismash.secondarymetabolites.org/help#transatpks) | 28804 | Difficidin biosynthetic gene cluster (46% of genes show similarity) |  |
| Cluster 4 | [Transatpks](http://antismash.secondarymetabolites.org/help#transatpks) | 23439 | Difficidin biosynthetic gene cluster (26% of genes show similarity) |  |
| Cluster 5 | [Transatpks](http://antismash.secondarymetabolites.org/help#transatpks) | 45749 | Difficidin biosynthetic gene cluster (46% of genes show similarity) | - |
| Cluster 6 | [T3pks](http://antismash.secondarymetabolites.org/help#t3pks) | 41109 | - | - |
| Cluster 7 | [Terpene](http://antismash.secondarymetabolites.org/help#terpene) | 21883 | - | - |
| Cluster 8 | [Nrps](http://antismash.secondarymetabolites.org/help#nrps) | 13087 | Fengycin biosynthetic gene cluster (20% of genes show similarity) |  |

Table S1. Continued.

| Strain clusters | Type | Length (bp) | Most similar known clusters | Predicted core clusters |
| --- | --- | --- | --- | --- |
| **Lx-11** | | | | |
| Cluster 9 | [Nrps](http://antismash.secondarymetabolites.org/help#nrps) | 10515 | Fengycin biosynthetic gene cluster (20% of genes show similarity) |  |
| Cluster 10 | [Nrps](http://antismash.secondarymetabolites.org/help#nrps)-[Transatpks](http://antismash.secondarymetabolites.org/help#transatpks) | 87792 | Fengycin biosynthetic gene cluster (80% of genes show similarity) |  |
| Cluster 11 | [Transatpks](http://antismash.secondarymetabolites.org/help#transatpks)-[Nrps](http://antismash.secondarymetabolites.org/help#nrps) | 102674 | Bacillaene biosynthetic gene cluster (92% of genes show similarity) |  |
| Cluster 12 | [Transatpks](http://antismash.secondarymetabolites.org/help#transatpks) | 85905 | Macrolactin biosynthetic gene cluster (90% of genes show similarity) | - |
| Cluster 13 | [Lantipeptide](http://antismash.secondarymetabolites.org/help#lantipeptide) | 28888 | - | - |
| Cluster 14 | [Terpene](http://antismash.secondarymetabolites.org/help#terpene) | 20740 | - | - |
| Cluster 15 | [Otherks](http://antismash.secondarymetabolites.org/help#otherks) | 41244 | Butirosin biosynthetic gene cluster (7% of genes show similarity) | - |
| Cluster 16 | [Nrps](http://antismash.secondarymetabolites.org/help#nrps) | 25425 | Surfactin biosynthetic gene cluster (39% of genes show similarity) | - |

Table S1. Continued.

| Strain clusters | Type | Length (bp) | Most similar known clusters | Predicted core clusters |
| --- | --- | --- | --- | --- |
| **Lx-11** | | | | |
| Cluster 17 | [Nrps](http://antismash.secondarymetabolites.org/help#nrps) | 9528 | Surfactin biosynthetic gene cluster (8% of genes show similarity) |  |
| Cluster 18 | [Nrps](http://antismash.secondarymetabolites.org/help#nrps) | 27976 | Surfactin biosynthetic gene cluster (43% of genes show similarity) |  |
| Cluster 19 | [Microcin](http://antismash.secondarymetabolites.org/help#microcin) | 1685 | - | - |

Table S1. Continued.

| Strain clusters | Type | Length (bp) | Most similar known clusters | Predicted core clusters |
| --- | --- | --- | --- | --- |
| **H57** | | | | |
| Cluster 1 | [Other](http://antismash.secondarymetabolites.org/help#other) | 41418 | Bacilysin biosynthetic gene cluster (85% of genes show similarity) | - |
| Cluster 2 | [Nrps](http://antismash.secondarymetabolites.org/help#nrps)-[Bacteriocin](http://antismash.secondarymetabolites.org/help#bacteriocin) | 66792 | Bacillibactin biosynthetic gene cluster (100% of genes show similarity) |  |
| Cluster 3 | [Transatpks](http://antismash.secondarymetabolites.org/help#transatpks) | 100450 | Difficidin biosynthetic gene cluster (93% of genes show similarity) |  |
| Cluster 4 | [T3pks](http://antismash.secondarymetabolites.org/help#t3pks) | 41166 | - | - |
| Cluster 5 | [Terpene](http://antismash.secondarymetabolites.org/help#terpene) | 21883 | - | - |
| Cluster 6 | [Nrps](http://antismash.secondarymetabolites.org/help#nrps) | 22079 | Plipastatin biosynthetic gene cluster (30% of genes show similarity) | - |
| Cluster 7 | [Nrps](http://antismash.secondarymetabolites.org/help#nrps) | 13899 | Fengycin biosynthetic gene cluster (26% of genes show similarity) |  |
| Cluster 8 | [Nrps](http://antismash.secondarymetabolites.org/help#nrps) | 10157 | Fengycin biosynthetic gene cluster (20% of genes show similarity) |  |

Table S1. Continued.

| Strain clusters | Type | Length (bp) | Most similar known clusters | Predicted core clusters |
| --- | --- | --- | --- | --- |
| **H57** | | | | |
| Cluster 9 | [Nrps](http://antismash.secondarymetabolites.org/help#nrps)-[Transatpks](http://antismash.secondarymetabolites.org/help#transatpks) | 88096 | Fengycin biosynthetic gene cluster (86% of genes show similarity) |  |
| Cluster 10 | [Transatpks](http://antismash.secondarymetabolites.org/help#transatpks)-[Nrps](http://antismash.secondarymetabolites.org/help#nrps) | 102683 | Bacillaene biosynthetic gene cluster (92% of genes show similarity) |  |
| Cluster 11 | [Transatpks](http://antismash.secondarymetabolites.org/help#transatpks) | 85896 | Macrolactin biosynthetic gene cluster (90% of genes show similarity) |  |
| Cluster 12 | [Terpene](http://antismash.secondarymetabolites.org/help#terpene) | 20740 | - | - |
| Cluster 13 | [Otherks](http://antismash.secondarymetabolites.org/help#otherks) | 41244 | - | - |
| Cluster 14 | [Thiopeptide](http://antismash.secondarymetabolites.org/help#thiopeptide)-[Lantipeptide](http://antismash.secondarymetabolites.org/help#lantipeptide) | 26819 | - | - |
| Cluster 15 | [Nrps](http://antismash.secondarymetabolites.org/help#nrps) | 65407 | Surfactin biosynthetic gene cluster (82% of genes show similarity) |  |

Table S1. Continued.

| Strain clusters | Type | Length (bp) | Most similar known clusters | Predicted core clusters |
| --- | --- | --- | --- | --- |
| **H57** | | | | |
| Cluster 16 | [Microcin](http://antismash.secondarymetabolites.org/help#microcin) | 10560 | - | - |

Table S1. Continued.

| Strain clusters | Type | Length (bp) | Most similar known clusters | Predicted core clusters |
| --- | --- | --- | --- | --- |
| **EBL11** | | | | |
| Cluster 1 | [Nrps](http://antismash.secondarymetabolites.org/help#nrps)-[Transatpks](http://antismash.secondarymetabolites.org/help#transatpks) | 87708 | Fengycin biosynthetic gene cluster (80% of genes show similarity) |  |
| Cluster 2 | [Transatpks](http://antismash.secondarymetabolites.org/help#transatpks)-[Nrps](http://antismash.secondarymetabolites.org/help#nrps) | 102686 | Bacillaene biosynthetic gene cluster (92% of genes show similarity) |  |
| Cluster 3 | [Terpene](http://antismash.secondarymetabolites.org/help#terpene) | 20740 | - | - |
| Cluster 4 | [Otherks](http://antismash.secondarymetabolites.org/help#otherks) | 41244 | Butirosin biosynthetic gene cluster (7% of genes show similarity) | - |
| Cluster 5 | Nrps | [48695](http://antismash.secondarymetabolites.org/help#nrps) | Plipastatin biosynthetic gene cluster (46% of genes show similarity) |  |
| Cluster 6 | [Terpene](http://antismash.secondarymetabolites.org/help#terpene) | 21883 | - | - |

Table S1. Continued.

| Strain clusters | Type | Length (bp) | Most similar known clusters | Predicted core clusters |
| --- | --- | --- | --- | --- |
| **EBL11** | | | | |
| Cluster 7 | [T3pks](http://antismash.secondarymetabolites.org/help#t3pks) | 41109 | - | - |
| Cluster 8 | [Transatpks](http://antismash.secondarymetabolites.org/help#transatpks) | 100463 | Difficidin biosynthetic gene cluster (100% of genes show similarity) |  |
| Cluster 9 | [Microcin](http://antismash.secondarymetabolites.org/help#microcin) | 14139 | - | - |
| Cluster 10 | [Nrps](http://antismash.secondarymetabolites.org/help#nrps) | 26065 | Surfactin biosynthetic gene cluster (47% of genes show similarity) | - |
| Cluster 11 | [Bacteriocin](http://antismash.secondarymetabolites.org/help#bacteriocin)-[Nrps](http://antismash.secondarymetabolites.org/help#nrps) | 51792 | Bacillibactin biosynthetic gene cluster (92% of genes show similarity) |  |
| Cluster 12 | [Microcin](http://antismash.secondarymetabolites.org/help#microcin) | 20148 | - | - |
| Cluster 13 | [Microcin](http://antismash.secondarymetabolites.org/help#microcin) | 26162 | - | - |
| Cluster 14 | [Lantipeptide](http://antismash.secondarymetabolites.org/help#lantipeptide) | 22615 | Locillomycin biosynthetic gene cluster (35% of genes show similarity) | - |
| Cluster 15 | [Nrps](http://antismash.secondarymetabolites.org/help#nrps) | 38528 | Surfactin biosynthetic gene cluster (52% of genes show similarity) |  |
| Cluster 16 | [Transatpks](http://antismash.secondarymetabolites.org/help#transatpks) | 73681 | Macrolactin biosynthetic gene cluster (100% of genes show similarity) |  |
| Cluster 17 | [Other](http://antismash.secondarymetabolites.org/help#other) | 41418 | Bacilysin biosynthetic gene cluster (100% of genes show similarity) | - |

Table S1. Continued.

| Strain clusters | Type | Length (bp) | Most similar known clusters | Predicted core clusters |
| --- | --- | --- | --- | --- |
| **EBL11** | | | | |
| Cluster 18 | [Nrps](http://antismash.secondarymetabolites.org/help#nrps) | 24630 | - | - |

Table S1. Continued.

| Strain clusters | Type | Length (bp) | Most similar known clusters | Predicted core clusters |
| --- | --- | --- | --- | --- |
| **JJC33M** | | | | |
| Cluster 1 | [T3pks](http://antismash.secondarymetabolites.org/help#t3pks) | 41109 | - | - |
| Cluster 2 | [Terpene](http://antismash.secondarymetabolites.org/help#terpene) | 21883 | - | - |
| Cluster 3 | [Nrps](http://antismash.secondarymetabolites.org/help#nrps)-[Transatpks](http://antismash.secondarymetabolites.org/help#transatpks) | 111326 | Fengycin biosynthetic gene cluster (93% of genes show similarity) |  |
| Cluster 4 | [Transatpks](http://antismash.secondarymetabolites.org/help#transatpks)-[Nrps](http://antismash.secondarymetabolites.org/help#nrps) | 102611 | Bacillaene biosynthetic gene cluster (92% of genes show similarity) |  |
| Cluster 5 | [Lantipeptide](http://antismash.secondarymetabolites.org/help#lantipeptide) | 41892 | - | - |
| Cluster 6 | [Nrps](http://antismash.secondarymetabolites.org/help#nrps) | 28450 | Surfactin biosynthetic gene cluster (47% of genes show similarity) |  |
| Cluster 7 | [Nrps](http://antismash.secondarymetabolites.org/help#nrps) | 25865 | Surfactin biosynthetic gene cluster (39% of genes show similarity) | - |
| Cluster 8 | [Otherks](http://antismash.secondarymetabolites.org/help#otherks) | 41244 | - | - |
| Cluster 9 | [Terpene](http://antismash.secondarymetabolites.org/help#terpene) | 13388 | - | - |

Table S1. Continued.

| Strain clusters | Type | Length (bp) | Most similar known clusters | Predicted core clusters |
| --- | --- | --- | --- | --- |
| **JJC33M** | | | | |
| Cluster 10 | [Bacteriocin](http://antismash.secondarymetabolites.org/help#bacteriocin)-[Nrps](http://antismash.secondarymetabolites.org/help#nrps) | 51782 | Bacillibactin biosynthetic gene cluster (92% of genes show similarity) |  |
| Cluster 11 | [Bacteriocin](http://antismash.secondarymetabolites.org/help#bacteriocin) | 10221 | - | - |
| Cluster 12 | [Transatpks](http://antismash.secondarymetabolites.org/help#transatpks) | 45539 | Etnangien biosynthetic gene cluster (12% of genes show similarity) |  |
| Cluster 13 | [Otherks](http://antismash.secondarymetabolites.org/help#otherks) | 22599 | Kalimantacin / batumin biosynthetic gene cluster (17% of genes show similarity) | - |
| Cluster 14 | [Transatpks](http://antismash.secondarymetabolites.org/help#transatpks) | 16299 | Bacillaene biosynthetic gene cluster (21% of genes show similarity) |  |
| Cluster 15 | [Nrps](http://antismash.secondarymetabolites.org/help#nrps) | 14225 | Plipastatin biosynthetic gene cluster (30% of genes show similarity) |  |
| Cluster 16 | [Nrps](http://antismash.secondarymetabolites.org/help#nrps) | 11143 | Fengycin biosynthetic gene cluster (20% of genes show similarity) |  |

Table S1. Continued.

| Strain clusters | Type | Length (bp) | Most similar known clusters | Predicted core clusters |
| --- | --- | --- | --- | --- |
| **JJC33M** | | | | |
| Cluster 17 | [Nrps](http://antismash.secondarymetabolites.org/help#nrps) | 9369 | Surfactin biosynthetic gene cluster (8% of genes show similarity) |  |
| Cluster 18 | [Microcin](http://antismash.secondarymetabolites.org/help#microcin) | 4590 | - | - |

Table S1. Continued.

| Strain clusters | Type | Length (bp) | Most similar known clusters | Predicted core clusters |
| --- | --- | --- | --- | --- |
| **DC-12** | | | | |
| Cluster 1 | [Otherks](http://antismash.secondarymetabolites.org/help#otherks) | 41244 | - | - |
| Cluster 2 | [Terpene](http://antismash.secondarymetabolites.org/help#terpene) | 20740 | - | - |
| Cluster 3 | [Lantipeptide](http://antismash.secondarymetabolites.org/help#lantipeptide) | 28889 | - | - |
| Cluster 4 | [Transatpks](http://antismash.secondarymetabolites.org/help#transatpks) | 85902 | Macrolactin biosynthetic gene cluster (100% of genes show similarity) |  |
| Cluster 5 | [Transatpks](http://antismash.secondarymetabolites.org/help#transatpks)-[Nrps](http://antismash.secondarymetabolites.org/help#nrps) | 82378 | Bacillaene biosynthetic gene cluster (100% of genes show similarity) |  |
| Cluster 6 | [Nrps](http://antismash.secondarymetabolites.org/help#nrps)-[Transatpks](http://antismash.secondarymetabolites.org/help#transatpks) | 87870 | Fengycin biosynthetic gene cluster (80% of genes show similarity) |  |
| Cluster 7 | [Nrps](http://antismash.secondarymetabolites.org/help#nrps) | 16981 | Fengycin biosynthetic gene cluster (20% of genes show similarity) |  |
| Cluster 8 | [Nrps](http://antismash.secondarymetabolites.org/help#nrps) | 6624 | - | - |
| Cluster 9 | [Nrps](http://antismash.secondarymetabolites.org/help#nrps) | 22444 | Plipastatin biosynthetic gene cluster (30% of genes show similarity) | - |

Table S1. Continued.

| Strain clusters | Type | Length (bp) | Most similar known clusters | Predicted core clusters |
| --- | --- | --- | --- | --- |
| **DC-12** | | | | |
| Cluster 10 | [Terpene](http://antismash.secondarymetabolites.org/help#terpene) | 21883 | - | - |
| Cluster 11 | [Transatpks](http://antismash.secondarymetabolites.org/help#transatpks) | 70591 | Difficidin biosynthetic gene cluster (86% of genes show similarity) |  |
| Cluster 12 | [Other](http://antismash.secondarymetabolites.org/help#other) | 41418 | Bacilysin biosynthetic gene cluster (85% of genes show similarity) | - |
| Cluster 13 | [Ladderane](http://antismash.secondarymetabolites.org/help#ladderane) | 29088 | - | - |
| Cluster 14 | [Nrps](http://antismash.secondarymetabolites.org/help#nrps) | 28075 | Surfactin biosynthetic gene cluster (47% of genes show similarity) |  |
| Cluster 15 | [Nrps](http://antismash.secondarymetabolites.org/help#nrps) | 9892 | Surfactin biosynthetic gene cluster (8% of genes show similarity) |  |
| Cluster 16 | [Nrps](http://antismash.secondarymetabolites.org/help#nrps) | 15667 | Surfactin biosynthetic gene cluster (39% of genes show similarity) | - |
| Cluster 17 | [Nrps](http://antismash.secondarymetabolites.org/help#nrps)-[Bacteriocin](http://antismash.secondarymetabolites.org/help#bacteriocin) | 66792 | Bacillibactin biosynthetic gene cluster (100% of genes show similarity) |  |
| Cluster 18 | [Microcin](http://antismash.secondarymetabolites.org/help#microcin) | 1595 | - | - |

Table S1. Continued.

| Strain clusters | Type | Length (bp) | Most similar known clusters | Predicted core clusters |
| --- | --- | --- | --- | --- |
| **CMW1** | | | | |
| Cluster 1 | [Nrps](http://antismash.secondarymetabolites.org/help#nrps)-[Transatpks](http://antismash.secondarymetabolites.org/help#transatpks) | 82729 | Fengycin biosynthetic gene cluster (93% of genes show similarity) |  |
| Cluster 2 | [Transatpks](http://antismash.secondarymetabolites.org/help#transatpks)-[Nrps](http://antismash.secondarymetabolites.org/help#nrps) | 102161 | Bacillaene biosynthetic gene cluster (92% of genes show similarity) |  |
| Cluster 3 | [Transatpks](http://antismash.secondarymetabolites.org/help#transatpks) | 85875 | Macrolactin biosynthetic gene cluster (90% of genes show similarity) | - |
| Cluster 4 | [Terpene](http://antismash.secondarymetabolites.org/help#terpene) | 20743 | - | - |
| Cluster 5 | [Otherks](http://antismash.secondarymetabolites.org/help#otherks) | 41244 | Butirosin biosynthetic gene cluster (7% of genes show similarity) | - |
| Cluster 6 | [Lantipeptide](http://antismash.secondarymetabolites.org/help#lantipeptide) | 22597 | - | - |
| Cluster 7 | [Ladderane](http://antismash.secondarymetabolites.org/help#ladderane) | 26618 | - | - |
| Cluster 8 | [Nrps](http://antismash.secondarymetabolites.org/help#nrps) | 65410 | Surfactin biosynthetic gene cluster (78% of genes show similarity) |  |

Table S1. Continued.

| Strain clusters | Type | Length (bp) | Most similar known clusters | Predicted core clusters |
| --- | --- | --- | --- | --- |
| **CMW1** | | | | |
| Cluster 9 | [Bacteriocin](http://antismash.secondarymetabolites.org/help#bacteriocin)-[Nrps](http://antismash.secondarymetabolites.org/help#nrps) | 51760 | Bacillibactin biosynthetic gene cluster (92% of genes show similarity) |  |
| Cluster 10 | [T3pks](http://antismash.secondarymetabolites.org/help#t3pks) | 41109 | - | - |
| Cluster 11 | [Terpene](http://antismash.secondarymetabolites.org/help#terpene) | 21883 | - | - |
| Cluster 12 | [Nrps](http://antismash.secondarymetabolites.org/help#nrps) | 21615 | Plipastatin biosynthetic gene cluster (30% of genes show similarity) | - |
| Cluster 13 | [Other](http://antismash.secondarymetabolites.org/help#other) | 41454 | Bacilysin biosynthetic gene cluster (100% of genes show similarity) | - |

Table S1. Continued.

| Strain clusters | Type | Length (bp) | Most similar known clusters | Predicted core clusters |
| --- | --- | --- | --- | --- |
| **B425** | | | | |
| Cluster 1 | [Nrps](http://antismash.secondarymetabolites.org/help#nrps)-[Transatpks](http://antismash.secondarymetabolites.org/help#transatpks) | 110407 | Fengycin biosynthetic gene cluster (93% of genes show similarity) |  |
| Cluster 2 | [Other](http://antismash.secondarymetabolites.org/help#other) | 41454 | Bacilysin biosynthetic gene cluster (85% of genes show similarity) | - |
| Cluster 3 | [Bacteriocin](http://antismash.secondarymetabolites.org/help#bacteriocin)-[Nrps](http://antismash.secondarymetabolites.org/help#nrps) | 51775 | Bacillibactin biosynthetic gene cluster (92% of genes show similarity) |  |
| Cluster 4 | [Lantipeptide](http://antismash.secondarymetabolites.org/help#lantipeptide) | 12918 | - | - |
| Cluster 5 | [Nrps](http://antismash.secondarymetabolites.org/help#nrps) | 9236 | Surfactin biosynthetic gene cluster (8% of genes show similarity) |  |
| Cluster 6 | [Nrps](http://antismash.secondarymetabolites.org/help#nrps) | 27387 | Surfactin biosynthetic gene cluster (43% of genes show similarity) |  |

Table S1. Continued.

| Strain clusters | Type | Length (bp) | Most similar known clusters | Predicted core clusters |
| --- | --- | --- | --- | --- |
| **B425** | | | | |
| Cluster 7 | [Nrps](http://antismash.secondarymetabolites.org/help#nrps) | 52076 | - |  |
| Cluster 8 | [T3pks](http://antismash.secondarymetabolites.org/help#t3pks) | 41109 | - | - |
| Cluster 9 | [Terpene](http://antismash.secondarymetabolites.org/help#terpene) | 21883 | - | - |
| Cluster 10 | [Nrps](http://antismash.secondarymetabolites.org/help#nrps)-[Transatpks](http://antismash.secondarymetabolites.org/help#transatpks) | 102689 | Bacillaene biosynthetic gene cluster (100% of genes show similarity) |  |
| Cluster 11 | [Nrps](http://antismash.secondarymetabolites.org/help#nrps) | 25284 | Surfactin biosynthetic gene cluster (39% of genes show similarity) | - |
| Cluster 12 | [Otherks](http://antismash.secondarymetabolites.org/help#otherks) | 41244 | - | - |
| Cluster 13 | [Terpene](http://antismash.secondarymetabolites.org/help#terpene) | 20743 | - | - |
| Cluster 14 | [Other](http://antismash.secondarymetabolites.org/help#other) | 1637 | - | - |

Table S1. Continued.

| Strain clusters | Type | Length (bp) | Most similar known clusters | Predicted core clusters |
| --- | --- | --- | --- | --- |
| **RD7-7** | | | | |
| Cluster 1 | [Microcin](http://antismash.secondarymetabolites.org/help#microcin) | 20148 | - | - |
| Cluster 2 | [Microcin](http://antismash.secondarymetabolites.org/help#microcin) | 20148 | - | - |
| Cluster 3 | [Microcin](http://antismash.secondarymetabolites.org/help#microcin) | 25975 | - | - |
| Cluster 4 | [Microcin](http://antismash.secondarymetabolites.org/help#microcin) | 26080 | - | - |
| Cluster 5 | Lantipeptide-Nrps-Transatpks | 83807 | Locillomycin biosynthetic gene cluster (35% of genes show similarity) |  |
| Cluster 6 | [Nrps](http://antismash.secondarymetabolites.org/help#nrps) | 65410 | Surfactin_biosynthetic_gene_cluster (82% of genes show similarity) |  |
| Cluster 7 | [Microcin](http://antismash.secondarymetabolites.org/help#microcin) | 20148 | - | - |
| Cluster 8 | [Microcin](http://antismash.secondarymetabolites.org/help#microcin) | 20148 | - | - |
| Cluster 9 | [Otherks](http://antismash.secondarymetabolites.org/help#otherks) | 41244 | - | - |
| Cluster 10 | [Terpene](http://antismash.secondarymetabolites.org/help#terpene) | 20743 | - | - |
| Cluster 11 | [Nrps](http://antismash.secondarymetabolites.org/help#nrps)-[Transatpks](http://antismash.secondarymetabolites.org/help#transatpks) | 102680 | Bacillaene biosynthetic gene cluster (100% of genes show similarity) |  |
| Cluster 12 | [Nrps](http://antismash.secondarymetabolites.org/help#nrps)-[Transatpks](http://antismash.secondarymetabolites.org/help#transatpks) | 76034 | Bacillomycin biosynthetic gene cluster (90% of genes show similarity) |  |

Table S1. Continued.

| Strain clusters | Type | Length (bp) | Most similar known clusters | Predicted core clusters |
| --- | --- | --- | --- | --- |
| **RD7-7** | | | | |
| Cluster 13 | [Terpene](http://antismash.secondarymetabolites.org/help#terpene) | 21883 | - | - |
| Cluster 14 | [T3pks](http://antismash.secondarymetabolites.org/help#t3pks) | 41154 | - | - |
| Cluster 15 | [Microcin](http://antismash.secondarymetabolites.org/help#microcin) | 20148 | - | - |
| Cluster 16 | [Nrps](http://antismash.secondarymetabolites.org/help#nrps) | 49731 | Bacillibactin biosynthetic gene cluster (92% of genes show similarity) |  |
| Cluster 17 | [Other](http://antismash.secondarymetabolites.org/help#other) | 41454 | Bacilysin biosynthetic gene cluster (100% of genes show similarity) | - |

Table S1. Continued.

| Strain clusters | Type | Length (bp) | Most similar known clusters | Predicted core clusters |
| --- | --- | --- | --- | --- |
| **S499** | | | | |
| Cluster 1 | Microcin | 20148 | - | - |
| Cluster 2 | Microcin | 20148 | - | - |
| Cluster 3 | Transatpks | 100438 | Difficidin biosynthetic gene cluster (93% of genes show similarity) |  |
| Cluster 4 | [T3pks](http://antismash.secondarymetabolites.org/help#t3pks) | 41109 | - | - |
| Cluster 5 | [Terpene](http://antismash.secondarymetabolites.org/help#terpene) | 21883 | - | - |
| Cluster 6 | [Nrps](http://antismash.secondarymetabolites.org/help#nrps)-[Transatpks](http://antismash.secondarymetabolites.org/help#transatpks) | 137829 | Fengycin biosynthetic gene cluster (100% of genes show similarity) |  |
| Cluster 7 | [Transatpks](http://antismash.secondarymetabolites.org/help#transatpks)-[Nrps](http://antismash.secondarymetabolites.org/help#nrps) | 102701 | Bacillaene biosynthetic gene cluster (92% of genes show similarity) |  |
| Cluster 8 | [Transatpks](http://antismash.secondarymetabolites.org/help#transatpks) | 85881 | Macrolactin biosynthetic gene cluster (90% of genes show similarity) | - |
| Cluster 9 | [Lantipeptide](http://antismash.secondarymetabolites.org/help#lantipeptide) | 28889 | - | - |
| Cluster 10 | [Terpene](http://antismash.secondarymetabolites.org/help#terpene) | 20740 | - | - |
| Cluster 11 | [Otherks](http://antismash.secondarymetabolites.org/help#otherks) | 41244 | Butirosin biosynthetic gene cluster (7% of genes show similarity) | - |
| Cluster 12 | [Microcin](http://antismash.secondarymetabolites.org/help#microcin) | 20148 | - | - |
| Cluster 13 | [Nrps](http://antismash.secondarymetabolites.org/help#nrps) | 65406 | Surfactin biosynthetic gene cluster (78% of genes show similarity) |  |

Table S1. Continued.

| Strain clusters | Type | Length (bp) | Most similar known clusters | Predicted core clusters |
| --- | --- | --- | --- | --- |
| **S499** | | | | |
| Cluster 14 | [Microcin](http://antismash.secondarymetabolites.org/help#microcin) | 20148 | - | - |
| Cluster 15 | [Microcin](http://antismash.secondarymetabolites.org/help#microcin) | 25975 | - | - |
| Cluster 16 | [Microcin](http://antismash.secondarymetabolites.org/help#microcin) | 20148 | - | - |
| Cluster 17 | [Microcin](http://antismash.secondarymetabolites.org/help#microcin) | 20148 | - | - |
| Cluster 18 | [Nrps](http://antismash.secondarymetabolites.org/help#nrps)-[Bacteriocin](http://antismash.secondarymetabolites.org/help#bacteriocin) | 66793 | Bacillibactin biosynthetic gene cluster (100% of genes show similarity) |  |
| Cluster 19 | [Other](http://antismash.secondarymetabolites.org/help#other) | 41418 | Bacilysin biosynthetic gene cluster (100% of genes show similarity) | - |

Table S1. Continued.

| Strain clusters | Type | Length (bp) | Most similar known clusters | Predicted core clusters |
| --- | --- | --- | --- | --- |
| **SRCM101266** | | | | |
| Cluster 1 | [Nrps](http://antismash.secondarymetabolites.org/help#nrps)-[Transatpks](http://antismash.secondarymetabolites.org/help#transatpks) | 110407 | Fengycin biosynthetic gene cluster (93% of genes show similarity) |  |
| Cluster 2 | [Nrps](http://antismash.secondarymetabolites.org/help#nrps)-[Bacteriocin](http://antismash.secondarymetabolites.org/help#bacteriocin) | 66736 | Bacillibactin biosynthetic gene cluster (100% of genes show similarity) |  |
| Cluster 3 | [Lantipeptide](http://antismash.secondarymetabolites.org/help#lantipeptide) | 22597 | - | - |
| Cluster 4 | [Other](http://antismash.secondarymetabolites.org/help#other) | 41454 | Bacilysin biosynthetic gene cluster (100% of genes show similarity) | - |
| Cluster 5 | [Nrps](http://antismash.secondarymetabolites.org/help#nrps) | 9848 | Surfactin biosynthetic gene cluster (8% of genes show similarity) |  |
| Cluster 6 | [Microcin](http://antismash.secondarymetabolites.org/help#microcin) | 1465 | - | - |
| Cluster 7 | [Other](http://antismash.secondarymetabolites.org/help#other) | 1067 | - | - |
| Cluster 8 | [Transatpks](http://antismash.secondarymetabolites.org/help#transatpks)-[Nrps](http://antismash.secondarymetabolites.org/help#nrps) | 102704 | Bacillaene biosynthetic gene cluster (92% of genes show similarity) |  |
| Cluster 9 | [Terpene](http://antismash.secondarymetabolites.org/help#terpene) | 20743 | - | - |
| Cluster 10 | [Otherks](http://antismash.secondarymetabolites.org/help#otherks) | 41244 | Butirosin biosynthetic gene cluster (7% of genes show similarity) | - |
| Cluster 11 | [Nrps](http://antismash.secondarymetabolites.org/help#nrps) | 25431 | Surfactin biosynthetic gene cluster (39% of genes show similarity) | - |

Table S1. Continued.

| Strain clusters | Type | Length (bp) | Most similar known clusters | Predicted core clusters |
| --- | --- | --- | --- | --- |
| **SRCM101266** | | | | |
| Cluster 12 | [Nrps](http://antismash.secondarymetabolites.org/help#nrps) | 51633 | - | - |
| Cluster 13 | [Terpene](http://antismash.secondarymetabolites.org/help#terpene) | 21883 | - | - |
| Cluster 14 | [T3pks](http://antismash.secondarymetabolites.org/help#t3pks) | 41109 | - | - |
| Cluster 15 | [Nrps](http://antismash.secondarymetabolites.org/help#nrps) | 28234 | Surfactin biosynthetic gene cluster (47% of genes show similarity) |  |

Table S1. Continued.

| Strain clusters | Type | Length (bp) | Most similar known clusters | Predicted core clusters |
| --- | --- | --- | --- | --- |
| **SRCM101294** | | | | |
| Cluster 1 | [Terpene](http://antismash.secondarymetabolites.org/help#terpene) | 21883 | - | - |
| Cluster 2 | [Nrps](http://antismash.secondarymetabolites.org/help#nrps)-[Transatpks](http://antismash.secondarymetabolites.org/help#transatpks) | 88316 | Fengycin biosynthetic gene cluster (93% of genes show similarity) |  |
| Cluster 3 | [Nrps](http://antismash.secondarymetabolites.org/help#nrps)-[Transatpks](http://antismash.secondarymetabolites.org/help#transatpks) | 102707 | Bacillaene biosynthetic gene cluster (92% of genes show similarity) |  |
| Cluster 4 | [Transatpks](http://antismash.secondarymetabolites.org/help#transatpks) | 85875 | Macrolactin biosynthetic gene cluster (90% of genes show similarity) |  |
| Cluster 5 | [Microcin](http://antismash.secondarymetabolites.org/help#microcin) | 1575 | - | - |
| Cluster 6 | [Nrps](http://antismash.secondarymetabolites.org/help#nrps) | 10498 | Lichenysin biosynthetic gene cluster (14% of genes show similarity) |  |
| Cluster 7 | [Terpene](http://antismash.secondarymetabolites.org/help#terpene) | 20743 | - | - |
| Cluster 8 | [Otherks](http://antismash.secondarymetabolites.org/help#otherks) | 41244 | Butirosin biosynthetic gene cluster (7% of genes show similarity) | - |
| Cluster 9 | [Nrps](http://antismash.secondarymetabolites.org/help#nrps) | 25183 | Surfactin biosynthetic gene cluster (39% of genes show similarity) | - |
| Cluster 10 | [Nrps](http://antismash.secondarymetabolites.org/help#nrps) | 49704 | Bacillibactin biosynthetic gene cluster (92% of genes show similarity) |  |

Table S1. Continued.

| Strain clusters | Type | Length (bp) | Most similar known clusters | Predicted core clusters |
| --- | --- | --- | --- | --- |
| **SRCM101294** | | | | |
| Cluster 11 | [Other](http://antismash.secondarymetabolites.org/help#other) | 41454 | Bacilysin biosynthetic gene cluster (100% of genes show similarity) | - |
| Cluster 12 | [Transatpks](http://antismash.secondarymetabolites.org/help#transatpks) | 23889 | - | - |
| Cluster 13 | [T3pks](http://antismash.secondarymetabolites.org/help#t3pks) | 41109 | - | - |
| Cluster 14 | [Nrps](http://antismash.secondarymetabolites.org/help#nrps) | 29293 | Surfactin biosynthetic gene cluster (47% of genes show similarity) |  |
| Cluster 15 | Lantipeptide | 22612 | Locillomycin biosynthetic gene cluster (28% of genes show similarity) | - |
